# Millimeter-Wave and High-Resolution Infrared Spectroscopy of the Ground and 14 Vibrationally Excited States Lying Below 1300 cm^–1^ of Pyrazole

**DOI:** 10.1021/acs.jpca.5c06339

**Published:** 2025-10-29

**Authors:** Brian J. Esselman, Maria A. Zdanovskaia, Jeff G. Crouse, Doyeon Kim, Brant E. Billinghurst, Dennis W. Tokaryk, R. Claude Woods, Robert J. McMahon

**Affiliations:** 1 Department of Chemistry, 5228 University of Wisconsin−Madison, 1101 University Avenue, Madison, Wisconsin 53706-1322, United States; 2 Department of Physics, 3427 University of New Brunswick, P.O. Box 4400, Fredericton, New Brunswick E3B 5A3, Canada; 3 Canadian Light Source Inc., University of Saskatchewan, Saskatoon, SK S7N 2 V3, Canada

## Abstract

The gas-phase rotational spectrum from 85 to 750 GHz and high-resolution infrared (IR) spectrum (Canadian Light Source) of 1*H*-pyrazole have been analyzed for the ground and vibrationally excited states lying below 1300 cm^–1^. The analysis benefits from the simultaneous analysis of rotational and high-resolution IR transitions that cover the same approximate ranges of *J* and *K*. In total, over 4400 transitions for the ground state have been measured, assigned, and least-squares fit to complete sextic centrifugally distorted-rotor Hamiltonian models. The presented ground-state rotational spectrum provides the foundation for astronomical searches across most of the frequency range covered by modern radiotelescopes. Additionally, the rotational and high-resolution infrared transitions of the 11 lowest-energy fundamental and three lowest-energy combination states have been measured, assigned, and least-squares fit. The four lowest-energy fundamental states (ν_21_, ν_20_, ν_19_, and ν_18_) are sufficiently separated in energy that they can all be well-treated by single-state Hamiltonians across the entire measured spectrum. The next four lowest-energy fundamental states (ν_17_, ν_16_, ν_15_, and ν_14_) form a Coriolis-coupled tetrad of states that are fit to a four-state model with six Coriolis interactions. The remaining vibrationally excited states investigated in this work (ν_13_, ν_12_, ν_11_, ν_21_ + ν_20_, ν_21_ + ν_19_, and ν_21_ + ν_18_) are treated by effective Hamiltonians, owing to their complex anharmonic- and Coriolis-coupling interactions. The experimental spectroscopic constants and vibrational energies are compared to their computed values (CCSD­(T)/cc-pCVTZ).

## Graphical abstract

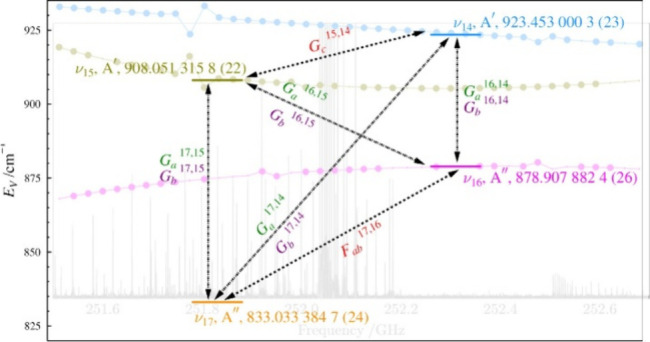

## Introduction

1*H*-Pyrazole (*c*-C_3_H_4_N_2_, *C*_s_) is a five-membered aromatic ring containing two adjacent sp^2^-hybridized nitrogen atoms, Figure 1. One of the nitrogen atoms is pyrrole-like with an attached hydrogen atom and lone pair conjugated to the aromatic π system. The other nitrogen atom is pyridine-like with a lone pair perpendicular to the aromatic π system. Pyrazole is one of two regioisomeric diazoles, the other being imidazole where the two nitrogen atoms are not adjacent. Both of these diazoles are important fundamental organic species and molecules that represent intriguing targets for interstellar detection by radioastronomy.^1,2^ Imidazole^3–5^ and other azoles^6–9^ have recently been investigated by high-resolution infrared and rotational spectroscopy. The current work updates the spectroscopy of pyrazole in a similar fashion.

**Figure 1:**
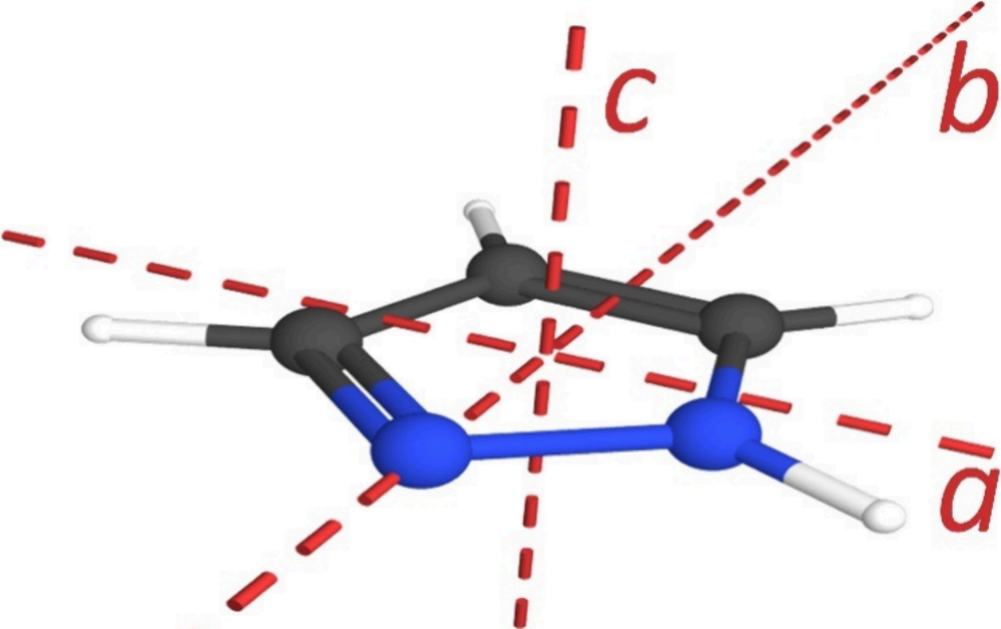
1*H*-Pyrazole (*c*-C_3_H_4_N_2_, *C*_s_, μ_*a*_ = 1.640 (13) D, μ_*b*_ = 1.488 (21) D, κ = 0.915)^10^ with principal axes.

The rotational spectrum of pyrazole was first investigated by Kirchoff in 1967. That work reported the spectrum from 8 to 35 GHz and measured the dipole moment (μ_*a*_ = 1.640 (13) D, μ_*b*_ = 1.488 (21) D) and rotational constants.^10^ Blackman *et al*. investigated the microwave spectrum of the normal and deuterium-substituted isotopologues and provided the nuclear quadrupole coupling constants for both nitrogen nuclei.^11,12^ In 1974, the complete substitution (*r*_*s*_) structure was obtained by two independent works using rotational constants from 12 isotopologues^13^ and 10 isotopologues,^14^ respectively. The nuclear quadrupole coupling constants were later refined during investigation of the rotational Zeeman effect.^15^ Wlodarczak *et al*. remeasured the microwave spectrum and extended the observed rotational spectrum up to 205 GHz, which enabled determination of a complete set of quartic centrifugal distortion constants.^16^ Using the available spectroscopic data, several semiexperimental equilibrium (*r*_*e*_^SE^) structures have been determined using DFT corrections for the vibration-rotation interaction and electron-mass corrections.^17–19^ The rotational spectra of the vibrationally excited states of pyrazole have not been previously reported.

The low-resolution infrared (IR) and Raman spectroscopy of pyrazole and its deuterium-substituted isotopologues has been investigated in the gas and condensed phases.^20–32^ The first assignments of the fundamental vibrational modes to their infrared frequencies was completed in 1967 by Zecchina *et al*.^20^ Fan and Boggs^26^ provided the first *ab initio* calculation of the vibrational force fields and supported the assignments made by previous authors.^20,21^ The solution-phase Raman spectroscopy and gas/condensed-phase IR spectroscopy were reported by Majoube,^28^ including a complete assignment of all vibrational modes. In a subsequent solid and liquid Raman study,^29^ some contradictory assignments of the vibrational modes were made, due in part to not observing the lowest-energy fundamental state. The earlier assignments^20,21,26,28^ were supported by two computational studies using DFT and coupled-cluster harmonic frequency calculations.^30,31^ No high-resolution infrared spectroscopy has been reported previously.

The current work presents a combined investigation of the rotational and high-resolution infrared spectroscopy of pyrazole, supported by DFT and coupled-cluster anharmonic frequency calculations. We provide rotational transitions for the ground vibrational state of pyrazole across nearly the entire range of modern radiotelescopes. We also provide precise and accurate vibrational frequencies and rotational constants for 14 of its 17 lowest-energy vibrationally excited states.

## Experimental Methods

1*H*-pyrazole was obtained commercially and used for spectroscopic analysis without further purification at the University of Wisconsin–Madison and the Canadian Light Source (CLS). Infrared spectra of pyrazole were collected at CLS, on the Far-Infrared Beamline, using a Bruker IFS 125 HR FTIR spectrometer equipped with a KBr beamsplitter and helium-cooled Ge:Cu detector. At room temperature, pyrazole has a relatively high vapor pressure of about 90 mTorr,^33^ so filling the 2-m-long White cell at the CLS to pressures suitable for spectroscopy (3, 9, and 18 mTorr) was straightforward. The cell’s mirrors were set to provide a total path length of 32 m. For data obtained through one filter with a bandpass of ∼350 to 750 cm^–1^, two spectra were collected: one at 3 mTorr pressure of pyrazole, for which 52 interferograms were obtained and averaged, and another at 9 mTorr, for which 162 scans were taken. With a second filter (400 to 1200 cm^–1^ bandpass), 104 scans were collected with a pressure of 9 mTorr, and 122 scans were obtained with a pressure of 18 mTorr. N_2_O calibration spectra were obtained in both regions. All spectra were taken with the full instrumental resolution of 0.000 96 cm^–1^, except for that of the background continuum, for which 100 scans were obtained at a resolution of 0.015 96 cm^–1^. Measurements of the centers of strong, well-resolved lines were assigned uncertainties of 0.000 10 to 0.000 13 cm^–1^, depending on whether the lines were instrument-limited or Doppler-limited, with weaker or blended lines assigned uncertainties of 1.25 to 2 times these values depending on the degree to which they were affected. Both Kisiel’s Assignment and Analysis of Broadband Spectra (AABS) software^34,35^ and Western’s PGOPHER^36^ were used to visualize, measure, and record transitions in the high-resolution infrared spectra.

At UW–Madison, broadband rotational spectra were obtained from 80 to 750 GHz using an instrument described previously, with nearly continuous coverage.^37–39^ Spectral segments were stitched together and analyzed using Kisiel’s AABS software.^34,35^ The complete spectrum from 80 to 750 GHz was obtained automatically over approximately 14 days, given the following experimental parameters: 0.6 MHz/s sweep rate, 10 ms time constant, and 50 kHz AM and 500 kHz FM modulation in a tone-burst design.^40^ Rotational frequencies measured in this work have an assumed uniform 50 kHz frequency measurement uncertainty. Spectroscopic data from both the high-resolution IR and rotational spectra were analyzed using SPFIT and SPCAT,^41^ as well as PIFORM, AC, PLANM, and other useful programs.^42^ We incorporated previously published transitions into the least-squares fits using the quoted experimental uncertainty.^10,11,16^ All output files from the least-squares fits are provided in the Supporting Information.

## Computational

An electronic structure calculation of pyrazole was carried out with Gaussian 16 ^43^ using the WebMO interface^44^ to obtain theoretical spectroscopic constants. The optimized geometry at the B3LYP/6-311+G­(2d,p) level was obtained using “verytight” convergence criteria and an “ultrafine” integration grid, and a subsequent anharmonic vibrational frequency calculation was carried out. Additional electronic structure calculations were carried out using a development version of CFOUR^45^ to obtain an optimized structure at the CCSD­(T)/cc-pCVTZ level of theory. The optimized geometry and the same level of theory were subsequently used for an anharmonic, second-order vibrational perturbation theory (VPT2) calculation, wherein cubic force constants are evaluated by numerical differentiation of analytic first derivatives at displaced points.^46–48^ These calculations provided fundamental frequencies, vibration–rotation interaction constants, Coriolis-coupling, and centrifugal distortion constants provided herein. Computational output files can be found in the Supporting Information.

## Results and Discussion

### Rotational Spectrum of Ground-State Pyrazole

Pyrazole is a near-oblate (κ = 0.915) asymmetric top of *C*_s_ symmetry with substantial *a*- and *b*-axis dipole moment components (μ_*a*_ = 1.640 (13) D, μ_*b*_ = 1.488 (21) D).^10^ As a result, its spectrum is dominated by *a*- and *b*-type R-branch transitions of comparable intensity. The *a*-type transitions reach a maximum intensity around 400 GHz, while the *b*-type transitions reach their maximum around 600 GHz (Figure 2). The frequency range covered in this experiment allows for the measurement of many *a*- and *b*-type transitions that share *J*/*K*_*a*_ quantum numbers. While these measurements provide redundant energy-level information to the data set, the different transition types allow for an excellent determination of rotational constants along all three principal axes. The rotational spectrum contains many transitions due to singly substituted heavy-atom isotopologues and vibrationally excited states. The rotational spectra of the [^13^C]- and [^15^N]-pyrazoles will be described in a subsequent work addressing the semiexperimental equilibrium structure.

**Figure 2:**
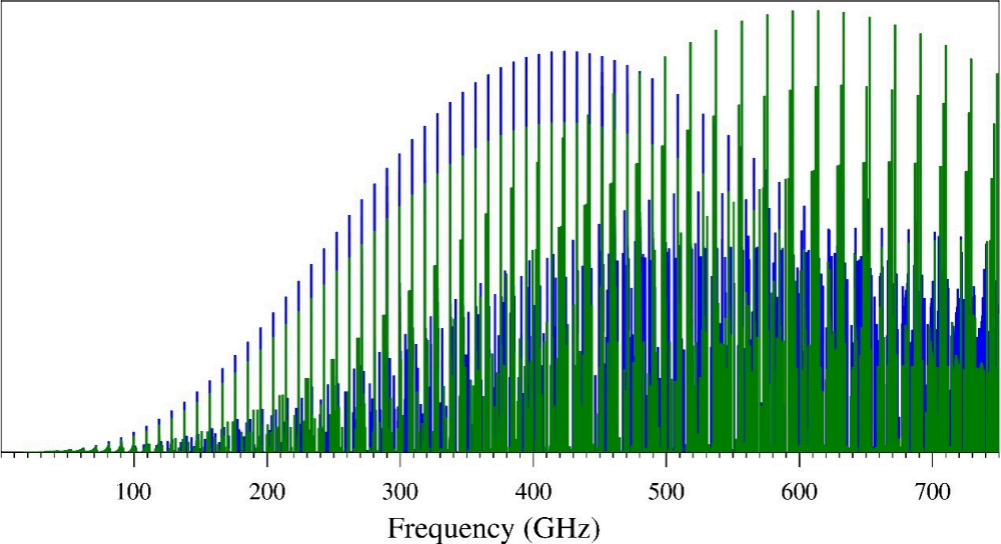
Predicted rotational spectrum of the ground vibrational state of pyrazole at 300 K with *a*-type transitions in blue and *b*-type transitions in green.

### Ground Vibrational State

The ground-state rotational spectrum of pyrazole displays the typical R-branch bands of an oblate asymmetric top (Figure 3). Each band starts from a degenerate set of two *a*-type (^*a*^R_0,1_) transitions and two *b*-type (^*b*^R_–1,1_ and ^*b*^R_1,1_) transitions with *K*_*a*_ = 0^+^/1^–^ (^+^ indicates transitions with *J* = *K*_*a*_ + *K*_*c*_ and ^–^ indicates transitions with *J* + 1 = *K*_*a*_ + *K*_*c*_). As *K*_*a*_ increases, the band transitions increase in frequency and eventually lose degeneracy forming well-resolved quartets. Each R-branch is accompanied by a lower-intensity Q-branch. The rotational spectrum of pyrazole has been analyzed previously using an A-reduced, partial sextic centrifugally distorted-rotor Hamiltonian in the I^*r*^ representation.^16^ To allow for convenient comparison to the previous work and support the subsequent vibrationally excited-state analysis, we provide the spectroscopic constants from a least-squares fit using the same Hamiltonian with a full set of sextic terms (Table 1). Additionally, we provide spectroscopic constants from an S-reduced, sextic centrifugally distorted-rotor Hamiltonian in the III^*r*^ representation. As an oblate species, the III representation might be most appropriate, however, a least-squares fit in that representation using the A reduction is unable to effectively model the high-*K*_*a*_, low-*K*_*c*_ transitions. Similar behavior was observed for other oblate asymmetric tops in that representation.^6,7,49^ Previously reported rotational transitions of pyrazole have been included in the data set^10,11,16^ for transitions that were not newly measured in this work. The transitions in the observed frequency range of this work do not include fully resolved nuclear-hyperfine transitions. Transitions where partially resolved splitting was observed were excluded from the least-squares data set, in a manner similar to Woldarczak *et al*.^16^ Presumably, the resolution was lower in the previous work, as many of the high-*K*_*a*_, low-*K*_*c*_ R- and Q-branch transitions that had partial splitting in our spectrum were reported as single-transition frequencies in that previous work. The data set distribution plot showing the complete range of *J* and *K* quantum numbers in the final data set is provided in Figure 4. Computed spectroscopic constants at the CCSD­(T)/cc-pCVTZ level are provided in Table 1 for comparison, and those calculated at the B3LYP/6-311+G­(2d,p) level of theory are provided in the Supporting Information (Table S1).

**Figure 3:**
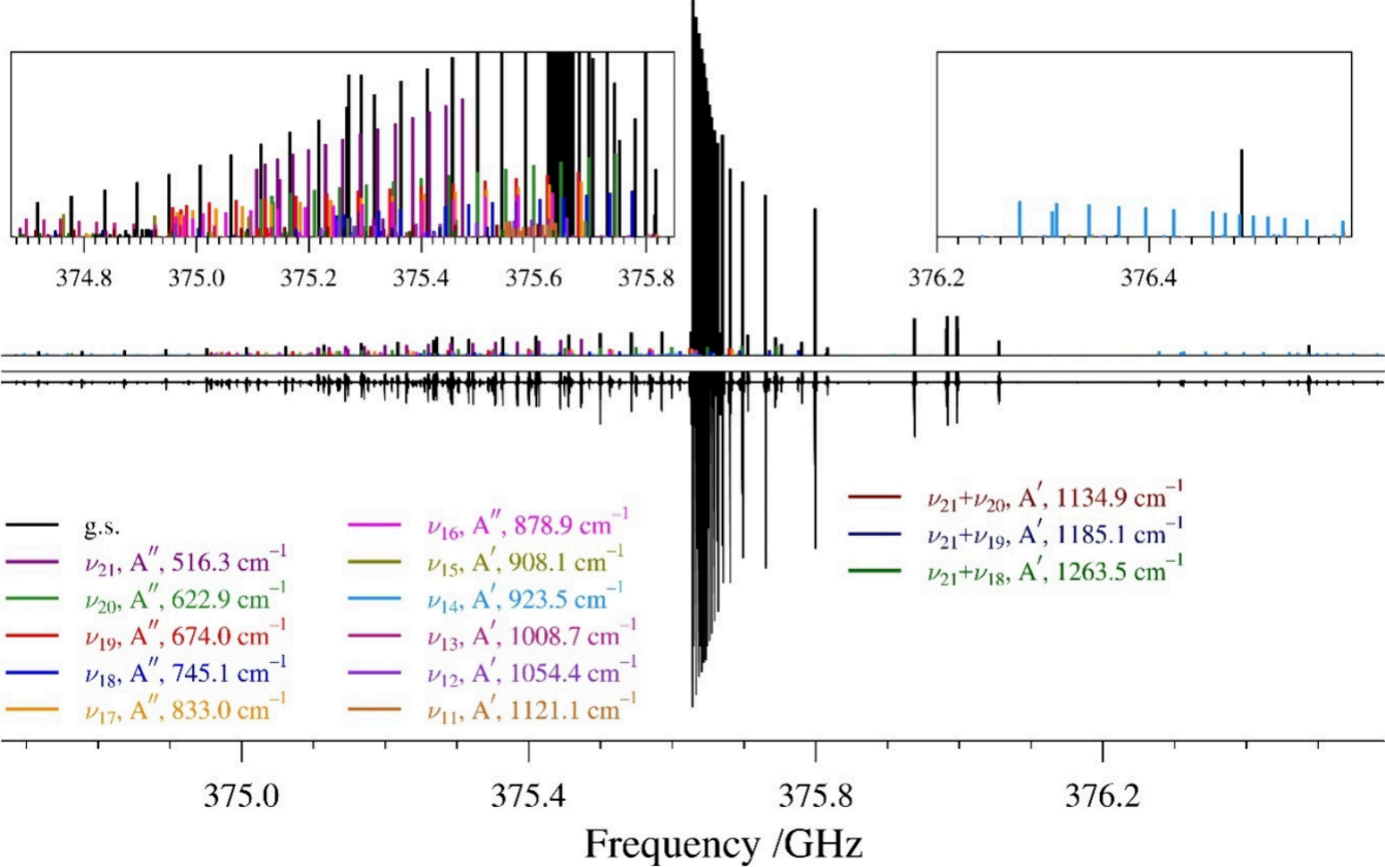
Experimental rotational spectrum (bottom) showing the *J*″ = 39 R-branch bands of pyrazole from 374.7 to 376.6 GHz and stick spectra (top) from experimental spectroscopic constants with the ground state (black), ν_21_ (purple), ν_20_ (green), ν_19_ (red), ν_18_ (blue), ν_17_ (gold), ν_16_ (pink), ν_15_ (olive), ν_14_ (light blue), ν_13_ (fuchsia), ν_12_ (violet), ν_11_ (orange), ν_21_ + ν_20_ (brown), ν_21_ + ν_19_ (navy), and ν_21_ + ν_18_ (dark green). Unassigned transitions are attributed to higher-energy vibrationally excited states and heavy-atom isotopologues. Insets are provided for improved visualization of low-intensity states.

**Table 1:** Spectroscopic Constants for the Ground Vibrational State of Pyrazole (A reduction, I^*r*^ representation and S reduction, III^*r*^ representation)

	CCSD(T)^a^	this work^b^	Wlodarczak et al.^16^		CCSD(T)^a^	this work^b^
*A*_0_^(A)^ (MHz)	9592	9618.773 480 (53)	9618.775 84 (84)	*A*_0_^(S)^ (MHz)	9592	9618.775 628 (40)
*B*_0_^(A)^ (MHz)	9357	9412.543 971 (48)	9412.543 81 (82)	*B*_0_^(S)^ (MHz)	9357	9412.541 470 (39)
*C*_0_^(A)^ (MHz)	4735	4755.849 164 (43)	4755.850 07 (98)	*C*_0_^(S)^ (MHz)	4735	4755.849 468 (43)
*Δ* *_J_* (kHz)	1.79	1.832 963 (25)	1.833 6 (16)	*D*_*J*_ (kHz)	3.22	3.276 232 (20)
*Δ_JK_* (kHz)	–0.550	–0.615 627 (93)	–0.609 8 (14)	*D*_*JK*_ (kHz)	–5.07	–5.164 096 (33)
*Δ_K_* (kHz)	1.82	1.873 249 (86)	1.868 8 (11)	*D*_*K*_ (kHz)	2.19	2.237 725 (20)
*δ* *_J_* (kHz)	0.722	0.741 551 (10)	0.741 19 (23)	*d*_1_ (kHz)	0.044 2	0.056 349 8 (53)
*δ_K_* (kHz)	1.26	1.276 153 (27)	1.276 80 (70)	*d*_2_ (kHz)	0.037 5	0.036 525 7 (19)
*Φ_J_* (Hz)	0.000 623	0.000 6584 (53)		*H*_*J*_ (Hz)	0.001 38	0.001 377 7 (30)
*Φ_JK_* (Hz)	0.000 660	0.000 266 (40)	0.002 93 (39)	*H*_*JK*_ (Hz)	–0.005 63	–0.005 731 3 (67)
*Φ_KJ_* (Hz)	–0.005 80	–0.005 582 (52)		*H*_*KJ*_ (Hz)	0.007 13	0.007 322 8 (67)
*Φ_K_* (Hz)	0.005 82	0.006 135 (44)		*H*_*K*_ (Hz)	–0.002 88	–0.002 978 6 (30)
*ϕ_J_* (Hz)	0.000 311	0.000 333 6 (25)		*h*_1_ (Hz)	0.000 005 5	[0.000 005 5]^c^
*ϕ* *_JK_* (Hz)	0.000 740	0.000 564 (14)		*h*_2_ (Hz)	–0.000 049 7	[−0.000 049 7]^c^
*ϕ_K_* (Hz)	0.002 35	0.002 394 (15)		*h*_3_ (Hz)	0.000 010 2	0.000 006 38 (89)
*N*_lines_ rot^d^		4456	121	*N*_lines_ rot^d^		4456
σ_fit_ (MHz)		0.037	0.050	σ_fit_ (MHz)		0.037
κ^e^		0.915 183	0.915 182	κ^e^		0.915 181
*Δ*_*i*_ (uÅ^2^)^f^		0.031 753 (1)	0.031 744 (23)	*Δ*_*i*_ (uÅ^2^)^f^		0.031 743 (1)

a Evaluated with the cc-pCVTZ basis set. Rotational constants (*B*_0_) are determined from their equilibrium (*B*_e_) values corrected for vibration-rotation interaction.

b Includes transitions from previous works.^10,11,16^

c Value held constant at the corresponding computed value.

d Number of independent transitions.

e κ = (2*B* – *A* – *C*)/(*A* – *C*) calculated from *B*_0_ values using PLANM.

f Inertial defect (*Δ*_*i*_ = *I*_*c*_ – *I*_*a*_ – *I*_*b*_) calculated from *B*_0_ values using PLANM.

**Figure 4:**
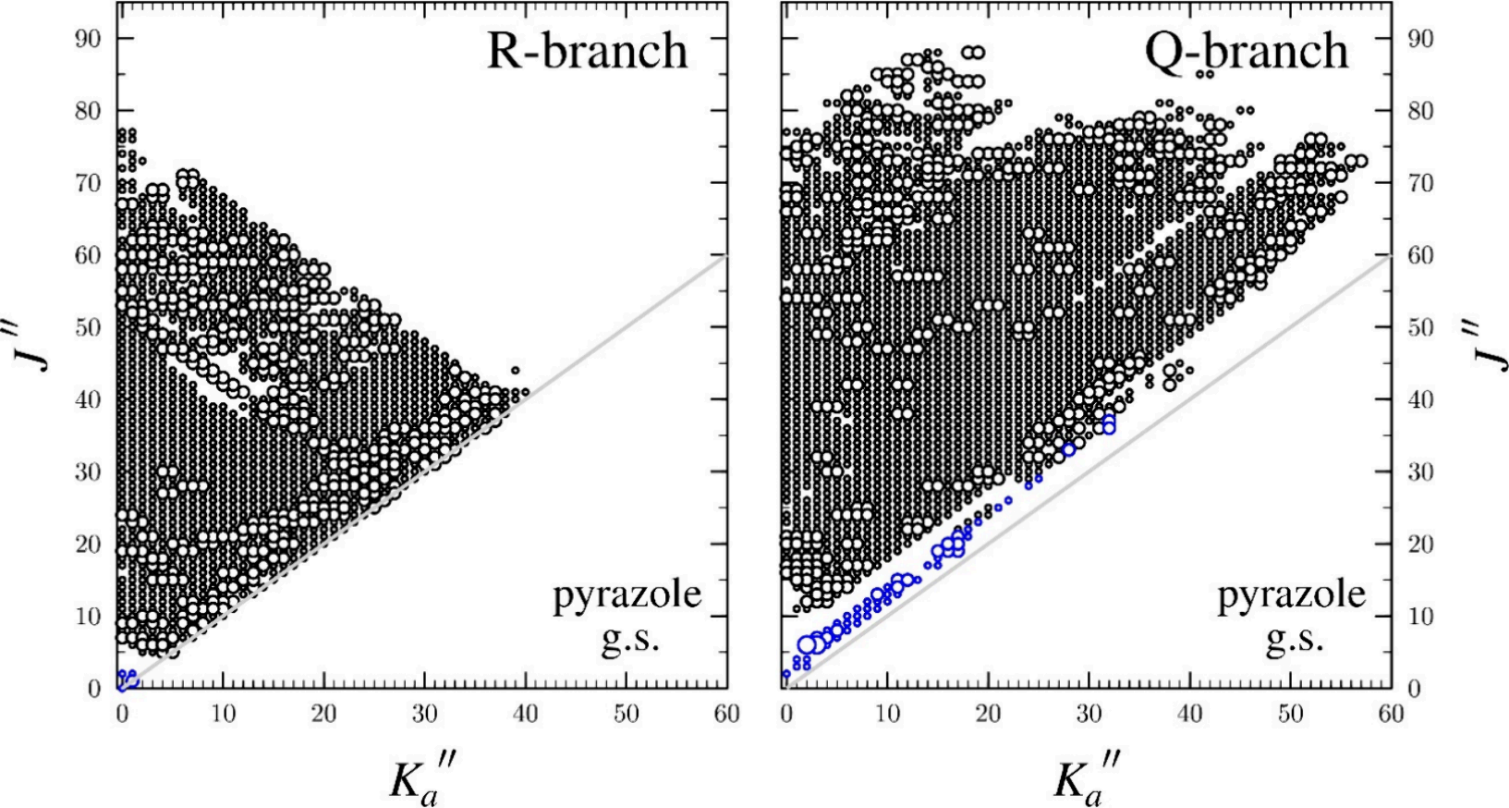
Data distribution plot for the least-squares fit of spectroscopic data for the vibrational ground state of pyrazole (A reduction, I^*r*^ representation). The size of the outlined circle is proportional to the value of |(*f*_obs._ – *f*_calc._)/δ*f*|, where δ*f* is the nominal frequency measurement uncertainty (50 kHz for newly measured transitions). None of the *obs*. – *calc.* values exceed 100 kHz. Blue circles represent data included from previous works, using the nominal frequency measurement uncertainty provided in these works.^10,11,16^

The previously reported rotational and centrifugal distortion constants^16^ are in the expected agreement with those from this work. The lone sextic constant (*Φ*_*JK*_) reported in that work is empirical, as it is larger by more than an order of magnitude than the value determined in this work and about five times larger than the computed value. The extended data set in this work allows for a complete determination of the A-reduction sextic centrifugal distortion constants in the I^*r*^ representation. In the S-reduction, *h*_2_ and *h*_3_ could not be determined and were held at their corresponding CCSD­(T) values. The computed rotational constants at the CCSD­(T) level are within 0.6% of the experimental values using either the A or S reduction (I^*r*^ and III^*r*^ representations, respectively). The absolute deviation of the computed from the experimental quartic centrifugal distortion constants is less than 3% for all constants except *d*_1_ (22%) and *D*_*JK*_ (11%). The sextic centrifugal distortion constants deviate by less than 7%, with the exceptions of *h*_3_ (60%), *Φ*_*JK*_ (160%) and *ϕ*_*JK*_ (32%). Efforts to hold these constants at their computed values in the least-squares fit increased the σ_fit_ value. The spectroscopic constants of the A-reduction ground-state fit are used in place of constants that cannot be determined in the least-squares fits of the vibrationally excited states.

### Rotational and IR Spectra of ν_21_, ν_20_, ν_19_, and ν_18_

The four lowest-energy vibrationally excited states of pyrazole (ν_21_, ν_20_, ν_19_, and ν_18_, Figure 5) are A″ vibrations (out-of-plane), which give rise to *c*-type infrared transitions. The infrared bands of each of these vibrational states have been measured in previous low-resolution gas-phase spectroscopy,^28,30^ with each separated by at least 50 cm^–1^ from its neighboring vibrational states. The lowest-energy state, ν_21_, is predominantly an N–H wag with small motions of the adjacent carbon and nitrogen atoms. The next fundamental state, ν_20_, is a ring-deformation mode with large opposite motions of the C–H and N–H bonds. Fundamental ν_19_ has a vibrational motion similar to ν_21_, but with larger motion of the hydrogenless nitrogen atom. The next fundamental state, ν_18_, displays large bends of both of the C–H bonds attached to the vinyl unit in the same direction. Rotational transitions and rotationally resolved infrared transitions for each of these states were fit using single-state, sextic-centrifugally distorted A-reduced Hamiltonians in the I^*r*^ representation. The A-reduced Hamiltonian was chosen for all of the vibrationally excited states to allow for straightfoward comparisons of the spectroscopic constants. Due to the energy separation, the least-squares fits of these single-state fits were similar to that of the ground vibrational state and the spectroscopic constants presented in Table 2 do not show indications of untreated couplings between the states. Data set distribution plots for all observed vibrational states are provided in Figures S1–S32.

**Figure 5:**
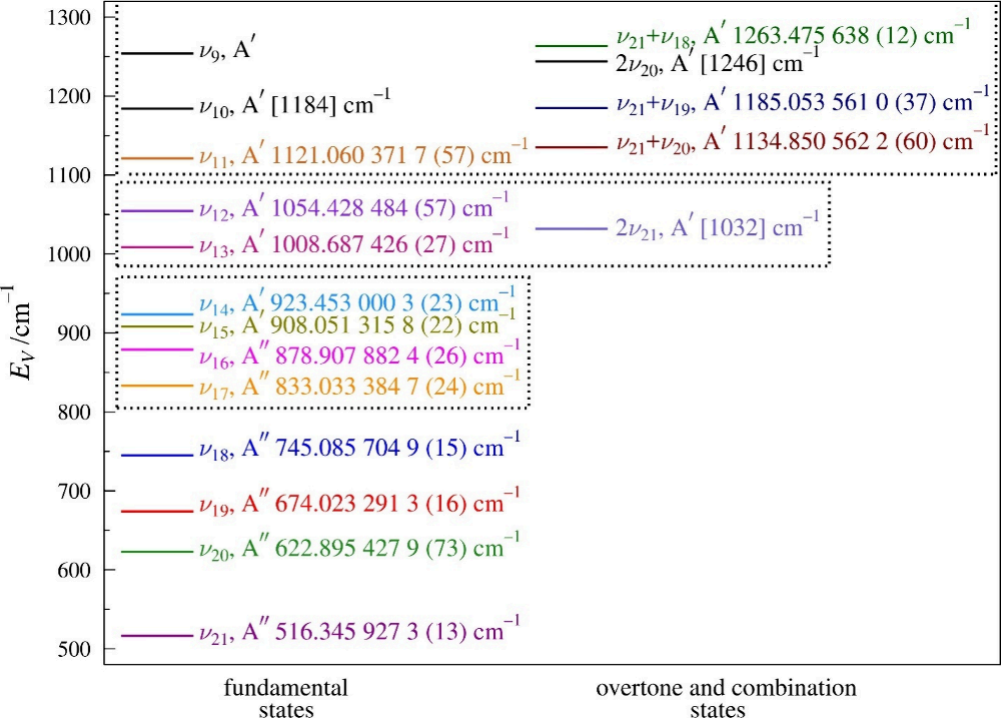
Vibrational energy levels determined in this work for pyrazole from 500 to 1300 cm^–1^. Boxes indicate groups of vibrationally excited states that exhibit Coriolis or anharmonic couplings between the enclosed states (*vide infra*). Vibrational energies of 2ν_21_, ν_10_, and 2ν_20_, in brackets, are tentative values based upon observed IR band locations or extrapolation.

**Table 2:**
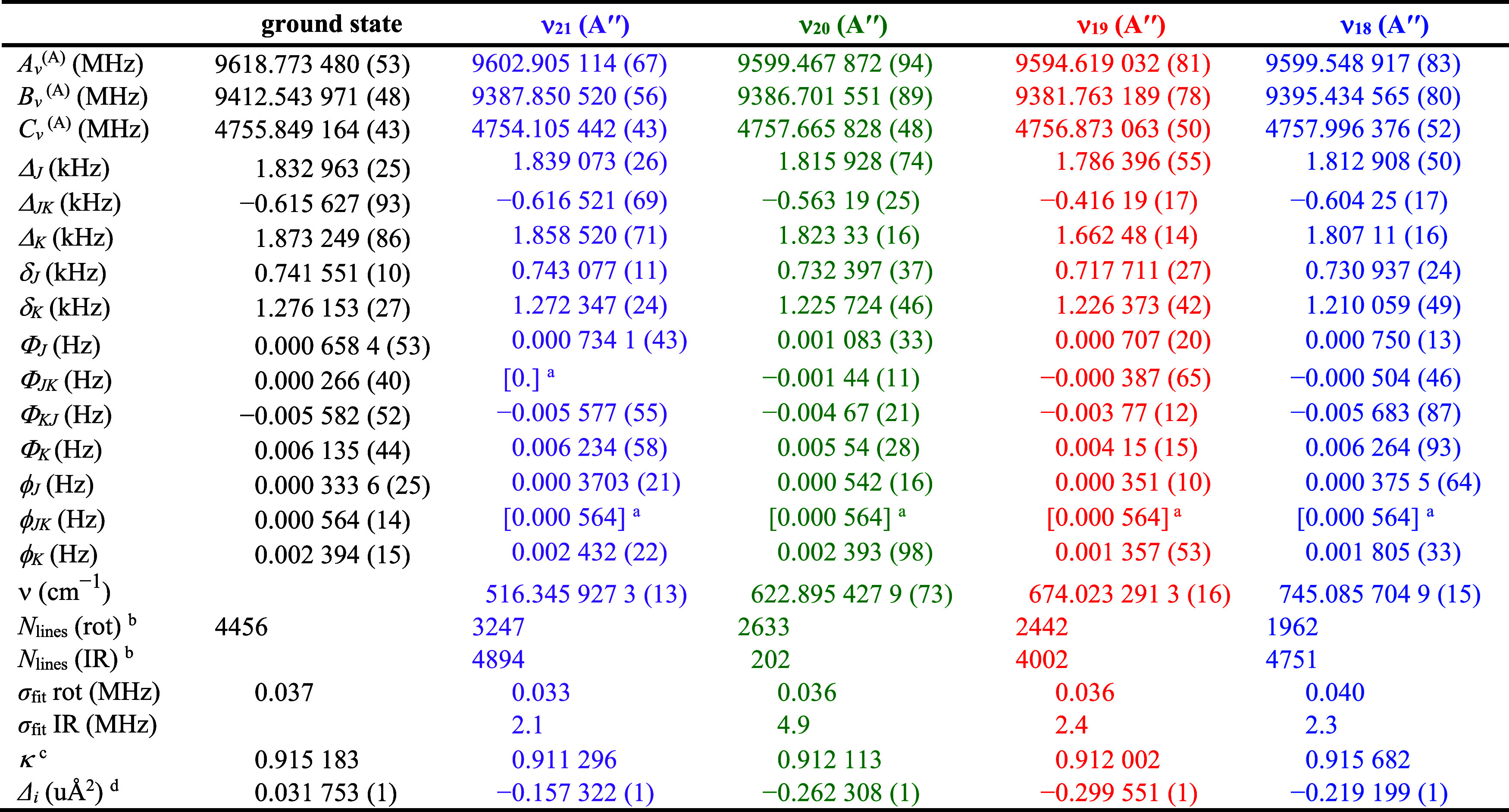
Spectroscopic Constants for the Ground, ν_21_, ν_20_, ν_19_, and ν_18_ Vibrational States of Pyrazole, A Reduction, I^*r*^ Representation

a Value held constant at zero (see discussion of constants and fitting) or the corresponding ground-state value.

b Number of independent transitions.

c κ = (2*B* – *A* – *C*)/(*A* – *C*).

d Inertial defect (*Δ*_*i*_ *= I*_*c*_ – *I*_*a*_ – *I*_*b*_).

The first fundamental state, ν_21_, has a very intense *c*-type IR spectrum, shown in Figure 6, and has rotational transitions approximately 8% as intense as that of the ground state (Figure 3). Initial predictions of its rotational and rotationally resolved IR spectra were made from the ground-state spectroscopic constants adjusted with the computed vibration-rotation interaction corrections. With its prominent P-, Q-, and R-branch IR bands and convenient spacing of the P- and R-branch subbands, the assignment of its infrared transitions was straightforward. In the rotational spectrum, the initial predictions allowed for easy assignment of the low-*K*_*a*_ series by use of Loomis–Wood plots. Due to the negative inertial defect of ν_21_, the transitions of its R-branch bands initially progress in the opposite direction to that of the ground state (to lower frequency with increasing *K*_*a*_). After initial transitions were measured and added to the data set, new predictions and measurements were made. Subsequent refinements of the spectroscopic constants and inclusion of newly assigned and measured transitions eventually included over 3240 rotational transitions and over 4890 high-resolution vibrational transitions. The spectroscopic constants of the ground state were held constant in simultaneous fitting of the rotational and IR transitions of ν_21_. The resulting least-squares fit had a σ_fit_ of 0.037 MHz for the rotational data (σ_fit_ rot) and a σ_fit_ for the IR data (σ_fit_ IR) of 2.1 MHz and produced the spectroscopic constants provided in Table 2. The two-orders of magnitude lower σ_fit_ value for the rotational transitions compared to the infrared transitions is due to the relative precision and accuracy of the measurements from each technique. While the rotational transitions are more precisely and accurately measured than the infrared transitions, these two techniques are highly complementary. Transitions from each technique provide similar information regarding the rotational constants and centrifugal distortion constants, with transitions having nearly the same range of *J* quantum numbers. The analysis of the high-resolution infrared spectrum, however, provides transitions with high-*K*_*a*_ and low-*K*_*a*_ quantum number values not represented in the rotational transitions of the excited state. The high-resolution infrared transitions provide precise information about the vibrational band origin (516.345 917 4 (16) cm^–1^), which can only be approximated by the transition intensities in the rotational spectrum. The spectroscopic constants of ν_21_ show the expected small deviations, with the exception of *Φ*_*JK*_, from their corresponding ground-state values. Although *Φ*_*JK*_ was attempted to be fitted, its value was very small, such that the uncertainty in its value was greater than the value itself. Holding its value constant at the ground-state value resulted in many excluded transitions and higher σ_fit_, so the constant was held constant at a value of zero. This may, however, not be an unreasonable estimate of the *Φ*_*JK*_ value for ν_21_ (*vide infra*). The results of an alternate least-squares fit of ν_21_ allowing *Φ*_*JK*_ to vary are provided in the Supporting Information and summarized in Table S2. The rotational constants and quartic centrifugal distortion constants all changed by less than 0.8%. Changes were larger in the sextic centrifugal distortion constants with *Φ*_*J*_, *ϕ*_*J*_, and *Φ*_*JK*_ deviating from the ground state by 16%, 265% and 15%, respectively. The origins of these deviations are not clear. The off-diagonal term *ϕ*_*JK*_ could not be determined and was held constant at its ground-state value. Holding this value constant has influenced the other constants, perhaps leading to these large deviations. Additionally, the data set may not be sufficient to determine physically meaningful values of these or other sextic centrifugal distortion constants. Efforts to hold any of the other sextic centrifugal distortion constants at their ground-state values led to larger σ_fit_ values in the least-squares fit, so these constants were allowed to vary in the least-squares fitting.

**Figure 6:**
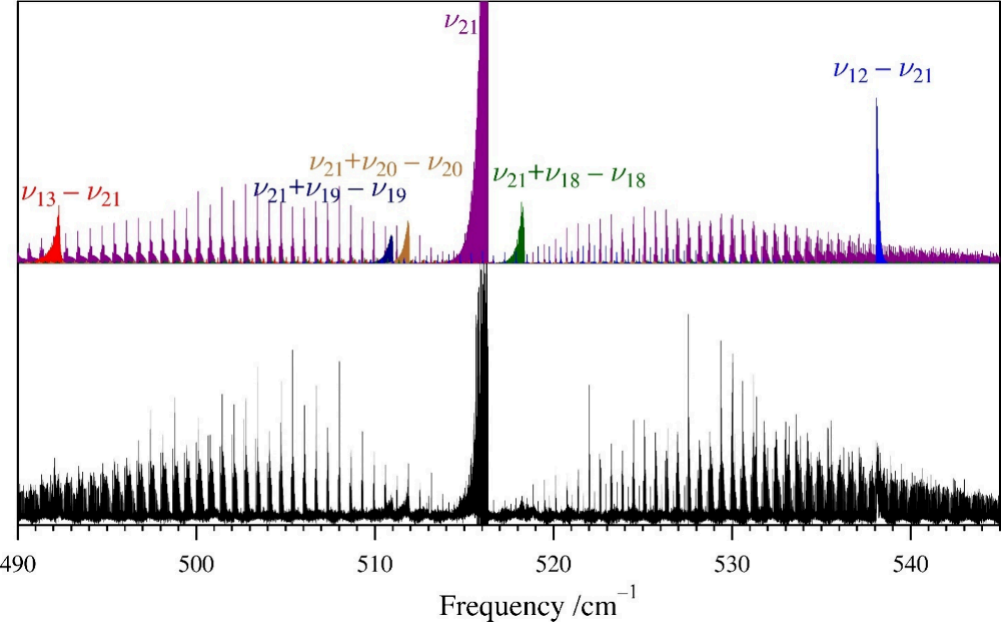
Pyrazole experimental high-resolution infrared spectrum (bottom) from 490 to 600 cm^–1^ at a pressure of 3 mTorr and predicted stick *c*-type spectrum of the ν_21_ band (purple, top) and associated bands from experimentally determined spectroscopic constants. Several additional vibrational bands involving ν_21_ are visible in the spectrum.

While the assignment of the infrared and rotational transitions to ν_21_ is straightforward, it is worth noting that it can be made independently based upon the computed or its previously measured vibrational energy. In the infrared spectrum, the ν_21_ band origin is unambiguous, and in the rotational spectra, its transitions are the most intense after those of the ground state. A least-squares fit of either data set results in approximately the same rotational and quartic centrifugal distortion constants. Additionally, the two data sets can be least-squares fit together at low error, providing additional support for the correct assignment. Finally, because ν_21_ is not impacted substantially by Coriolis coupling with other vibrational states, comparison of the computed vibration-rotation interaction constants (*B*_0_ – *B*_*v*_, Table 3) with the experimental ones confirms the assignment. The agreement between the CCSD­(T) vibration-rotation interaction constants and their corresponding experimental values is excellent, with differences of –0.43, 0.13, and 0.13 MHz for *A*_0_ – *A*_*v*_, *B*_0_ – *B*_*v*_, and *C*_0_ – *C*_*v*_, respectively. The agreement is slightly poorer for the B3LYP computed values, which deviate by as much as 1.90 MHz for *A*_0_ – *A*_*v*_. Despite these differences, either computational method would be sufficient to assign transitions of ν_21_ or make the initial spectral predictions.

**Table 3:**
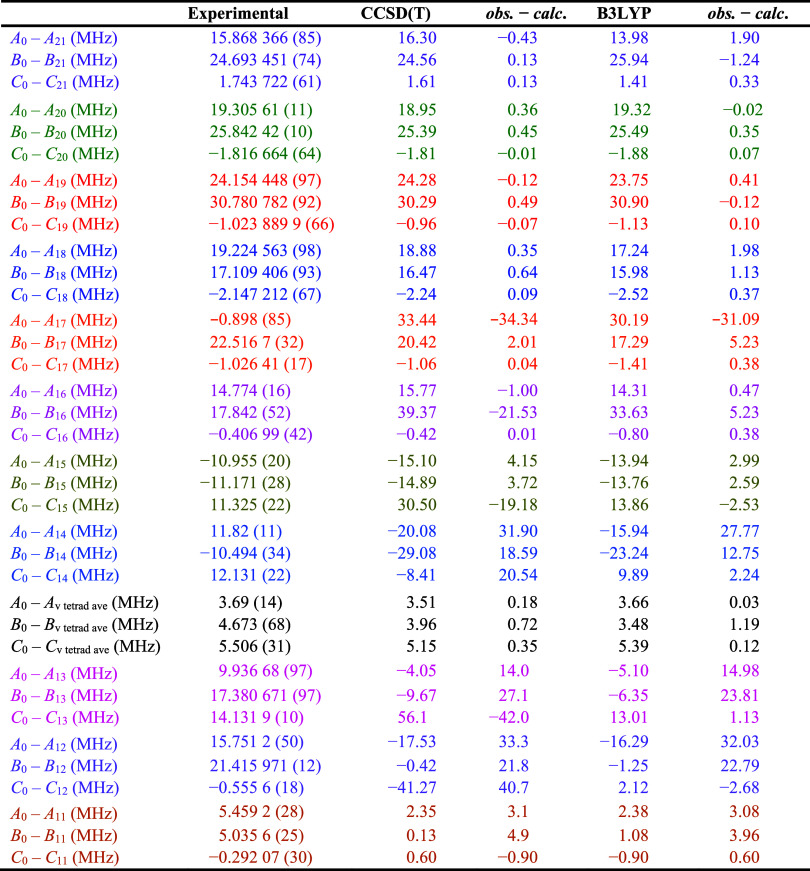
Experimental and Computed Vibration–Rotation Interaction Constants for Excited Vibrational States of Pyrazole

The next three fundamental states, ν_20_ (A″, 622.895 427 9 (73) cm^–1^), ν_19_ (A″, 674.023 291 3 (16) cm^–1^), and ν_18_ (A″, 745.085 704 9 (15) cm^–1^), were all predicted, assigned, and least-squares fit using a similar manner to ν_21_. Due to the exceptionally small IR intensity of ν_20_, however, its high-resolution IR transitions were not assigned until after the analysis of its rotational spectrum. Figure 7 shows the predicted and experimental IR spectra of all three of these states. At a scale convenient for ν_18_, the spectrum of ν_20_ is not discernible. The ν_20_ transitions are barely discernible from the P-branch transitions of ν_19_ and its hot band *ca*. 50 cm^–1^ higher in frequency. IR transitions of ν_20_ were measured using the highest-pressure scans of that frequency range (18 mTorr), and even then, only *ca*. 200 transitions can be measured due to low signal-to-noise ratio. A Loomis–Wood plot showing the assignment of the ν_20_ transitions is provided in Figure S33. Data distribution plots for the rotational and high-resolution infrared transitions for each of these states are available in the Supporting Information.

**Figure 7:**
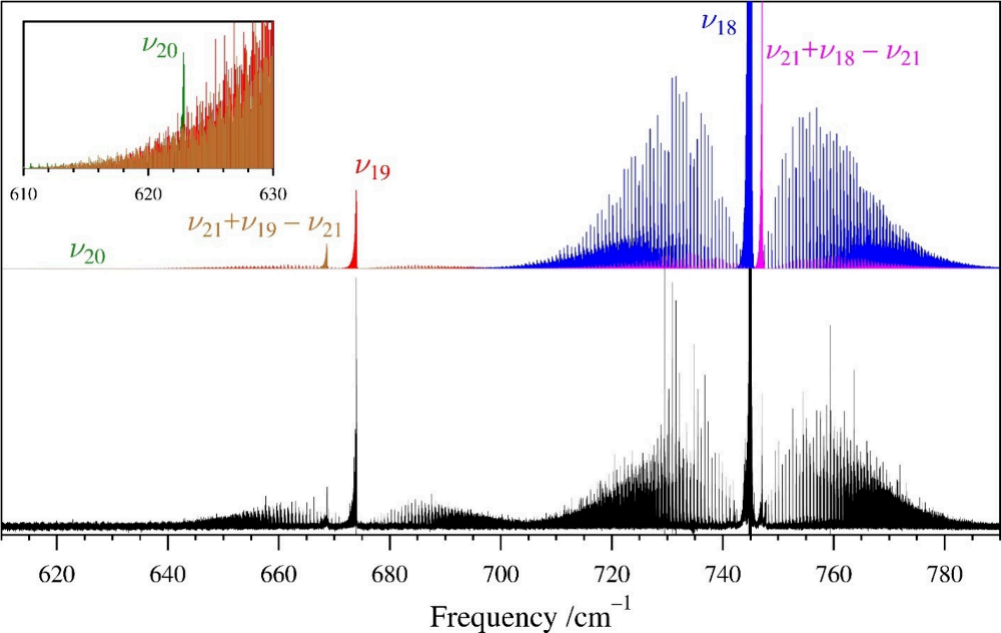
Pyrazole experimental high-resolution infrared spectrum (bottom) from 610 to 790 cm^–1^ at a pressure of 3 mTorr and predicted stick *c*-type spectra of ν_20_, ν_19_, and ν_18_ (green, red, and blue, respectively, top) from experimentally determined spectroscopic constants. Several additional vibrational bands involving ν_19_ and ν_18_ are visible in the spectrum. An inset is provided to enable identification of the ν_20_ band, as it is not detectable on a scale convenient for the ν_18_ band, especially as the ν_20_ band appears amidst ν_19_ and its associated hot band.

The final least-squares fit of these states resulted in the spectroscopic constants provided in Table 2, which all show the expected small changes in the rotational and quartic centrifugal distortion constants relative to the ground state. The rotational constants change by less than 0.33% and these shifts have very small *obs*. – *calc*. values when compared to the computed vibration-rotation interaction values in Table 3. The quartic centrifugal distortion constants show larger deviations from the ground state for all three of these vibrational states than did ν_21_. For example, the *Δ*_*JK*_ value for ν_19_ is 32% smaller than its corresponding ground-state value. Given the large numbers of transitions in each data set and the low σ_fit_ values, these changes are likely to be physically meaningful. The set of quartic spectroscopic constants in Table 2 provides an interesting set of experimental benchmarks for the ongoing efforts to provide accurate computational predictions of quartic centrifugal distortion constants of vibrationally excited states.^9^ The observed changes in the sextic centrifugal distortion constants relative to the ground state are also larger for these vibrational states than for ν_21_. Interestingly, while the value of *Φ*_*JK*_ could not be determined for ν_21_, it could be determined for all three of these higher-energy vibrationally excited states. In each case, the value has changed sign relative to the ground-state value. Given the A″ symmetry of all of these vibrational modes, it may be the case that the out-of-plane vibrational motions all decrease the *Φ*_*JK*_ value with respect to the ground state, placing its value near zero for ν_21_ and negative for ν_20_, ν_19_, and ν_18_. With the value of *ϕ*_*JK*_ held at its ground-state value for each of these vibrationally excited states, it is expected that each of the other sextic centrifugal distortion constants may be slightly influenced. Nevertheless, it is likely that all of the sextic centrifugal distortion constants presented in Table 2 have some physical meaning and could be used as benchmarks in future computational studies. Like for ν_21_, inspection of all of the spectroscopic constants, the σ_fit_ values, and Loomis–Wood plots of the transitions provide confidence that ν_20_, ν_19_, and ν_18_ are being reasonably treated by single-state distorted-rotor Hamiltonians.

### Rotational and IR Spectra of ν_17_, ν_16_, ν_15_, and ν_14_

The next lowest-energy vibrationally excited states of pyrazole (ν_17_, ν_16_, ν_15_, and ν_14_, Figure 5) form a Coriolis-coupled tetrad of two A″ fundamental states, ν_17_ (A″, 833.033 384 7 (24) cm^–1^) and ν_16_ (A″, 878.907 882 4 (26) cm^–1^), and the two lowest-energy A′ vibrational states, ν_15_ (A′, 908.051 315 8 (22) cm^–1^) and ν_14_ (A′, 923.453 000 3 (23) cm^–1^). The vibrational energies of these four fundamental states fall within a range of 90 cm^–1^, with no vibrational state isolated by more than 45 cm^–1^. As a result, transitions of each state display substantial perturbations due to coupling interactions of nearby rotational energy levels of the other states in this tetrad. Initial prediction, measurement, and least-squares fitting of ν_17_ proceeded in the same manner as described for the other A″ vibrational states in the rotational and high-resolution infrared spectra. After initial treatment of ν_17_ by a single-state, distorted rotor Hamiltonian, it became clear that a single-state model was inadequate to address the curvature of ν_17_ *K*_*a*_ series in the observed millimeter-wave spectrum. The 45 cm^–1^ separation of ν_17_ from ν_16_ was insufficient to prevent Coriolis interactions from perturbing the observed rotational frequencies. This was not initially expected, because an energy separation of 52 cm^–1^ is sufficient to allow ν_20_ and ν_19_ to be satisfactorily least-squares fit without addressing any interaction between these states. Due to the easily discernible IR bands for ν_16_, ν_15_, and ν_14_ shown in Figure 8, assignment of their rotationally resolved IR spectra was much more straightforward than their rotational spectra. These three states experience sufficiently intense Coriolis couplings that the initial predictions of their rotational spectra from their computed vibration-rotation interaction constants was problematic. Use of Loomis–Wood plots allowed all three states to be identified in the rotational spectrum, but did not allow their conclusive assignment based solely on the vibration-rotation interaction constants from the initial fits. Combined use of IR and rotational data allowed for many thousands of transitions in both data sets to be assigned and included in a four-state least-squares fit.

**Figure 8:**
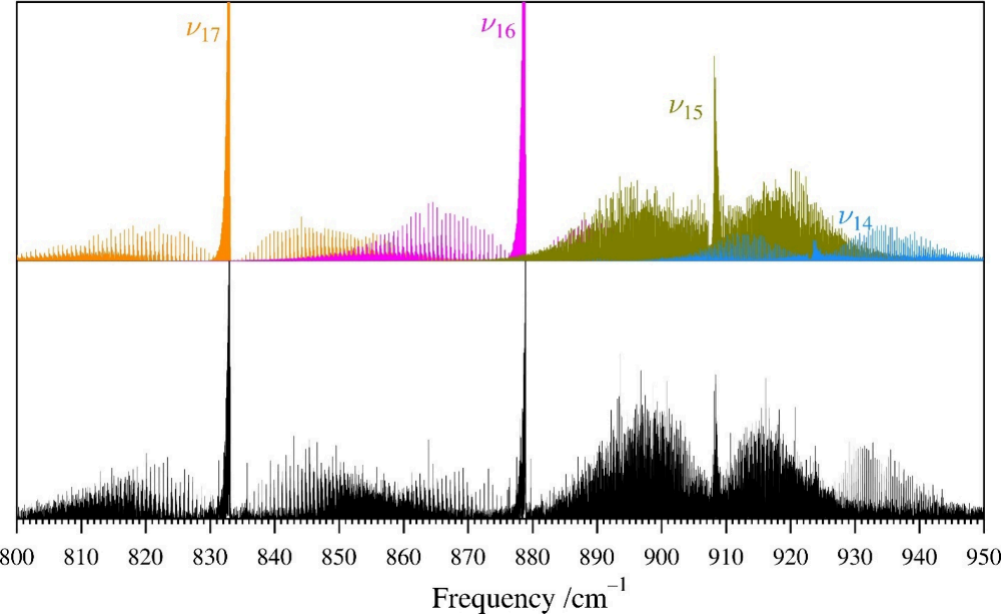
Pyrazole experimental high-resolution infrared spectrum (bottom) from 800 to 950 cm^–1^ at a pressure of 9 mTorr and predicted stick *c*-type spectra of ν_17_ and ν_16_ (orange and pink, respectively) and the *a*-type and *b*-type spectra of ν_15_ and ν_14_ (olive and light blue, respectively) on top from experimentally determined spectroscopic constants. Transitions from hot bands in this region were observable but were too weak and perturbed to be assigned in the current work.

Due to the interactions between these four fundamental states, a four-state, Coriolis-coupled, A-reduced Hamiltonian model was employed with sextic-level centrifugal distortion in the I^*r*^ representation. The four-state Coriolis-coupling model employed is displayed in Figure 9. All of the A′ states interact by both *a*- and *b*-axis Coriolis coupling with the A″ states. States with same symmetry (ν_14_ and ν_15_ or ν_17_ and ν_16_) interact by *c*-axis Coriolis coupling. Initial predictions of the Coriolis coupling coefficients (*G*_*x*_) were made from the computed Coriolis-ζ constants. While *c*-axis Coriolis-coupling is allowed between ν_17_ and ν_16_ as a result of their A″ symmetries, their both being out-of-plane vibrational motions confers a Coriolis-ζ value and concomitantly first-order *G*_*c*_ value of zero. This coupling can be addressed by a small *G*_*c*_ value created by higher-order contributions or by *F*_*ab*_.

**Figure 9:**
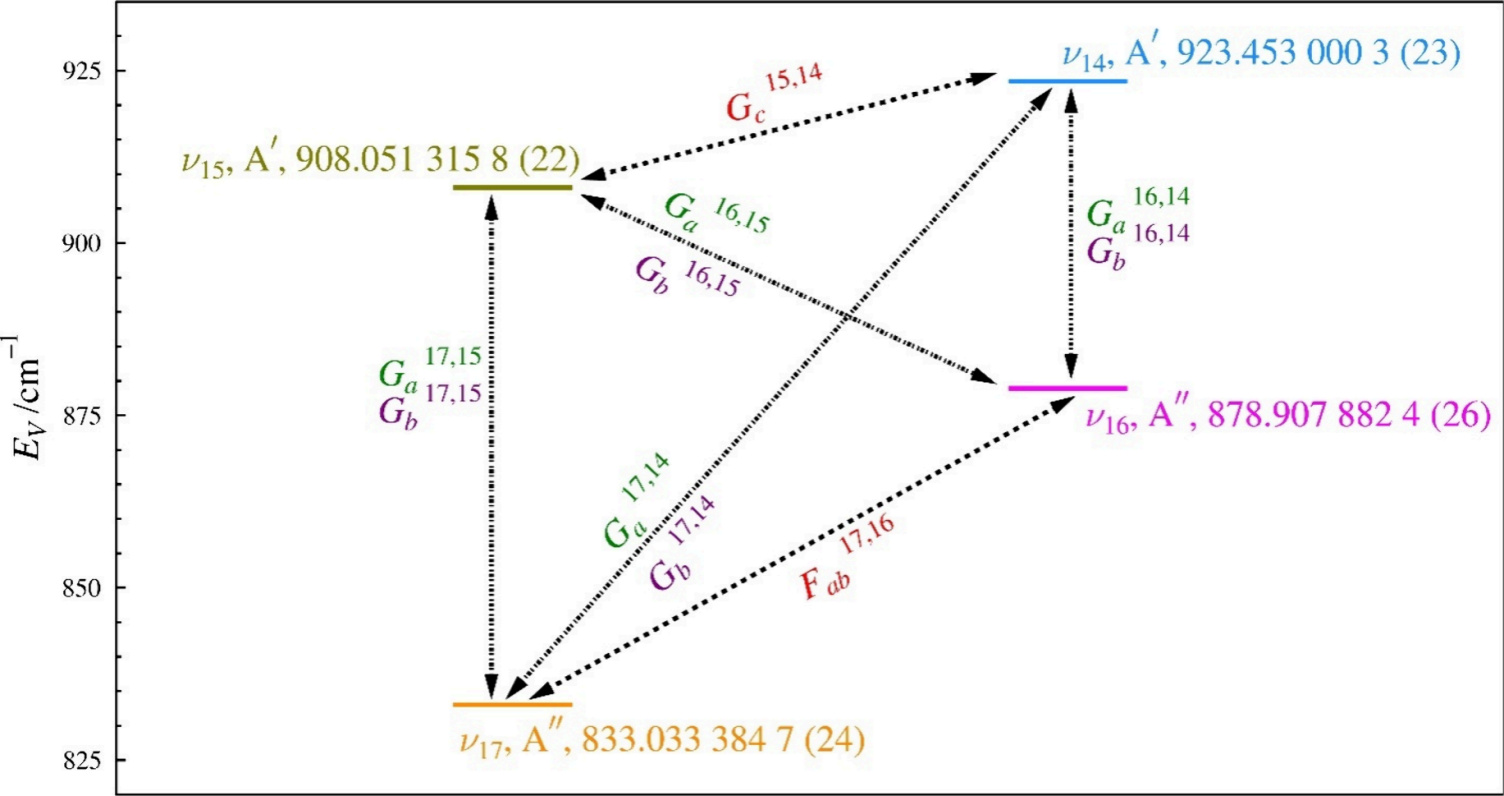
Coriolis-coupling interactions between ν_17_, ν_16_, ν_15_, and ν_14_. All numerical values are in cm^–1^.

The least-squares fitting of the tetrad of ν_17_, ν_16_, ν_15_, and ν_14_ proceeded in the expected manner of a Coriolis-coupled polyad. The high-resolution infrared data provided critical constraints on the vibrational band origin of each mode. Initial inclusion of the high-resolution infrared transitions and minimally perturbed rotational transitions allowed for a sufficient determination of the spectroscopic constants to predict additional rotational transitions. As additional transitions were added to the data set, additional centrifugal distortion constants and Coriolis-coupling terms were allowed to vary. Eventually, this iterative process allowed for assignment of highly perturbed resonant transitions of the type shown in Figure 10 between the three states closest in energy (ν_16_, ν_15_, and ν_14_). These *K*_*a*_ series display a *c*-type Coriolis resonance (Δ*K*_*a*_ = 1, Δ*K*_*c*_ = 2) between ν_14_ and ν_15_. The same *K*_*a*_ series of ν_14_ also displays an *a*-type Coriolis resonance (Δ*K*_*a*_ = 2, Δ*K*_*c*_ = 3) with ν_16_. A resonance progression plot of the even *K*_*a*_ series for ν_14_ is provided in Figure S34 showing the increasing intensity of the resonances with increasing *J*″ + 1 and *K*_*a*_. No displaced resonant transitions were identified involving ν_17_, though the Coriolis-coupling interactions did create the aforementioned global curvature of that state. Additionally, a total of 15 mixing-allowed, nominal-interstate rotational transitions were identified and included in the data set between ν_15_ and ν_14_. An example of the nominal interstate transitions and the relation to their corresponding in-state resonant transitions is provided in Figure S35.

**Figure 10:**
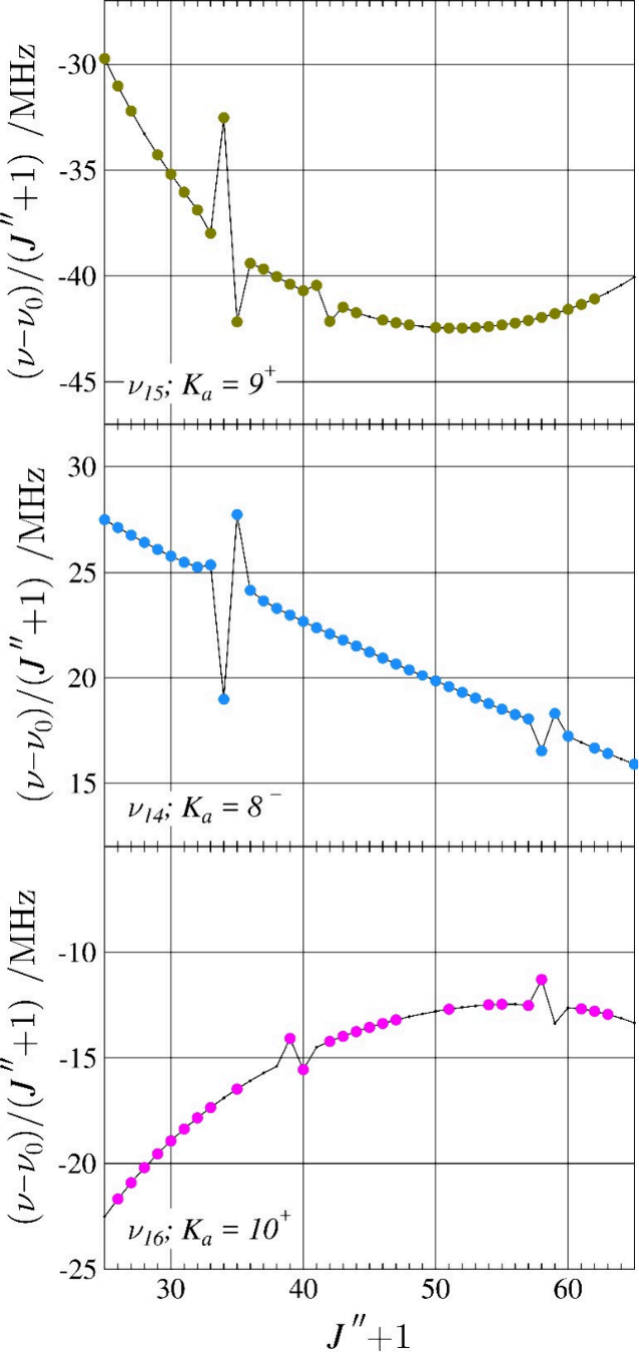
Resonance plots for pyrazole showing the *K*_*a*_ = 10^+^ series (pink) for ν_16_, *K*_*a*_ = 8^–^ series (light blue) for ν_14_, and the *K*_*a*_ = 9^+^ series (olive) for ν_15_. There are no transitions for which the *obs*. – *calc.* value exceeds 150 kHz or 3 times the nominal experimental uncertainty. The plotted values are frequency differences between excited-state transitions and their ground-state counterparts (ν – ν_0_), scaled by (*J*″ + 1) in order to make the plots more horizontal. Predictions from the final coupled fit are represented by a solid, black line.

The final spectroscopic constants from the least-squares fit of ν_17_, ν_16_, ν_15_, and ν_14_ are provided in Tables 4 and 5. The rotational constants and resultant vibration–rotation interaction constants of these states do not show close agreement with their corresponding computed values (Table 3). The discrepancy between these values is based upon both the computed values not being deperturbed^46^ and the impact of imperfectly treated Coriolis-coupling in the experimental values. The agreement between the average computed and experimental values of all four states is much closer to the expected level at both the CCSD­(T) and B3LYP levels of theory. The discrepancies are all less than 1 MHz for the CCSD­(T) values and just over 1 MHz for the B3LYP values. This closer agreement indicates that these vibrational states are being modeled reasonably, though not necessarily physically meaningfully, by both computational methods. The largest discrepancy is *A*_0_ – *A*_17_, which is consistent with large *a*-axis Coriolis coupling between ν_17_ and both ν_15_ and ν_14_. Similarly, ν_15_ and ν_14_ are involved in *c*-axis Coriolis coupling and exhibit large discrepancies between their experimental and computed vibration-rotation interaction constants. All of the quartic centrifugal distortion constants of the tetrad vibrational states, except for δ_*J*_, show larger deviations from the ground-state values than the four lower-energy vibrationally excited states treated as distorted rotors. These deviations suggest that the distortion constants have absorbed some coupling, and it is possible that the experimental rotational constants are not completely free of Coriolis coupling, either. As a result, the spectroscopic constants determined for the vibrationally excited states of the tetrad are likely effective to some extent, rather than completely physically meaningful.

**Table 4:**
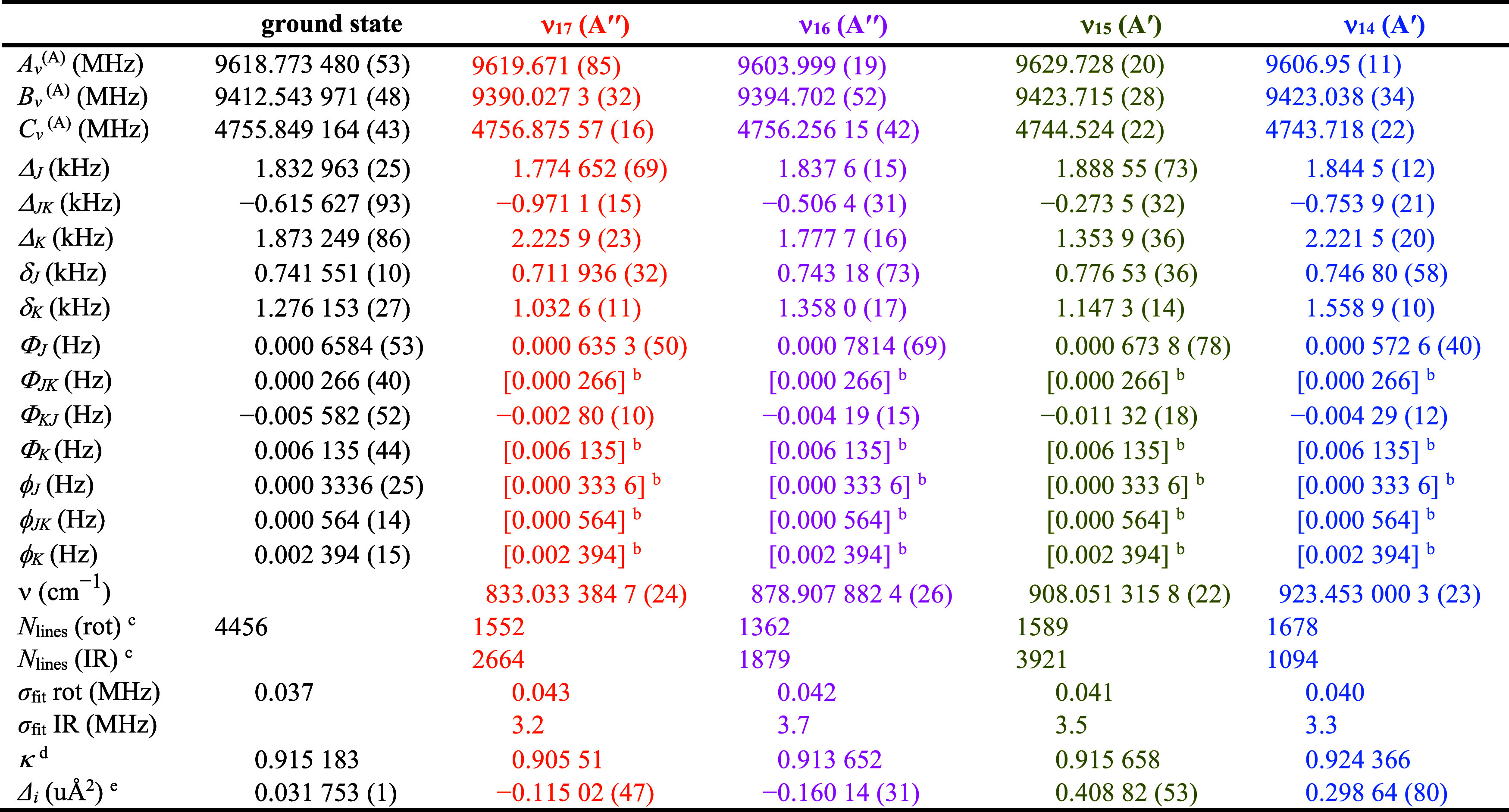
Spectroscopic Constants for the Ground, ν_17_, ν_16_, ν_15_, and ν_14_ Vibrational States of Pyrazole, A Reduction, I^*r*^ Representation^a^

a Computed and experimental Coriolis-coupling constants for these states are provided in Table 5.

b Value held constant at the corresponding ground-state value.

c Number of independent transitions.

d κ = (2*B* – *A* – *C*)/(*A* – *C*).

e Inertial defect (*Δ*_*i*_ *= I*_*c*_ – *I*_*a*_ – *I*_*b*_).

**Table 5:**
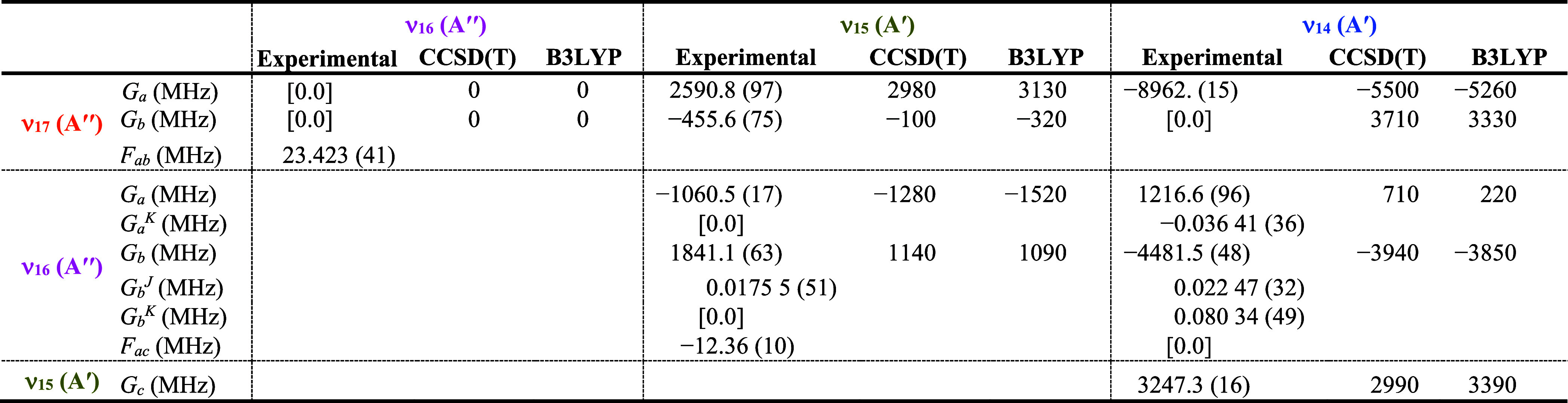
Coriolis-Coupling Terms for the ν_17_, ν_16_, ν_15_, and ν_14_ Vibrational States of Pyrazole, A Reduction, I^*r*^ Representation

Analysis of the Coriolis-coupling terms provided in Table 5 supports this assumption that the tetrad fit is to some extent effective, particularly with regard to the interaction of ν_17_ with ν_14_, where the agreement between the computed and experimental *G*_*a*_ values is quite poor and the value of *G*_*b*_ is held at zero despite a predicted value of over 3000 MHz. It is difficult to model the interaction of ν_17_ with the other vibrational states, due to the lack of observed resonances in the data set. This difficulty is particularly pronounced with ν_14_, which is nearly 230 cm^–1^ higher in energy than ν_17_. The agreement is slightly closer for all *G*_*x*_ constants involving ν_16_ between their computed and experimental values, where the discrepancy is up to 40% for the CCSD­(T) values and 92% for the B3LYP values. As expected, if the source of the discrepancy is poorly determined experimental values, the closest agreement between theory and experiment occurs for the *G*_*c*_ constant between ν_15_ and ν_14_, which have the strongest resonances and the most nominal interstate transitions included in the data set.

### Rotational and IR Spectra of ν_13_, 2ν_21_, and ν_12_

The next three vibrationally excited states in increasing energy (Figure 5) are two A′ fundamentals, ν_13_ (1008.687 426 (27) cm^–1^) and ν_12_ (1054.428 484 (57) cm^–1^), and the first overtone, 2ν_21_ (expected to be approximately 2 × ν_21_ or 1032 cm^–1^, A′). The rotational spectrum prediction of these two fundamental states from the ground-state rotational constants and the computed vibration-rotation interaction constants did not lead to successful detection and assignment. The initial nondetection was due to their low relative intensity, many of their most intense transitions appearing in a particularly dense region of the spectrum, and their coupling interactions with each other (*c*-axis Coriolis) and with 2ν_21_ (Coriolis and Fermi coupling). The prediction and assignment of the rotational spectrum of 2ν_21_ by extrapolation of the spectroscopic constants of the ground state and ν_21_ was similarly unsuccessful, due to those couplings. The assignment of the high-resolution IR transitions (Figure 11) for both fundamental states provided a set of spectroscopic constants that did allow their rotational spectra to be assigned. It became quickly apparent that the transitions of these two fundamental states were highly perturbed, displaying curving *K*_*a*_ series in Loomis–Wood plots of the rotational spectrum and clear local resonances in the rotational and IR spectra. Figure S36 shows a progression of resonances in the high-resolution infrared R-branches of ν_12_, which have been tentatively attributed to interactions with 2ν_21_. A few low-*K*_*a*_ series of rotational transitions have been tentatively assigned to 2ν_21_. Attempts to include these transitions in the least-squares fits were unsuccessful due to their Coriolis and Fermi interactions. Due to the ambiguity of these assignments, rotational transitions of 2ν_21_ are not presented in this work.

**Figure 11:**
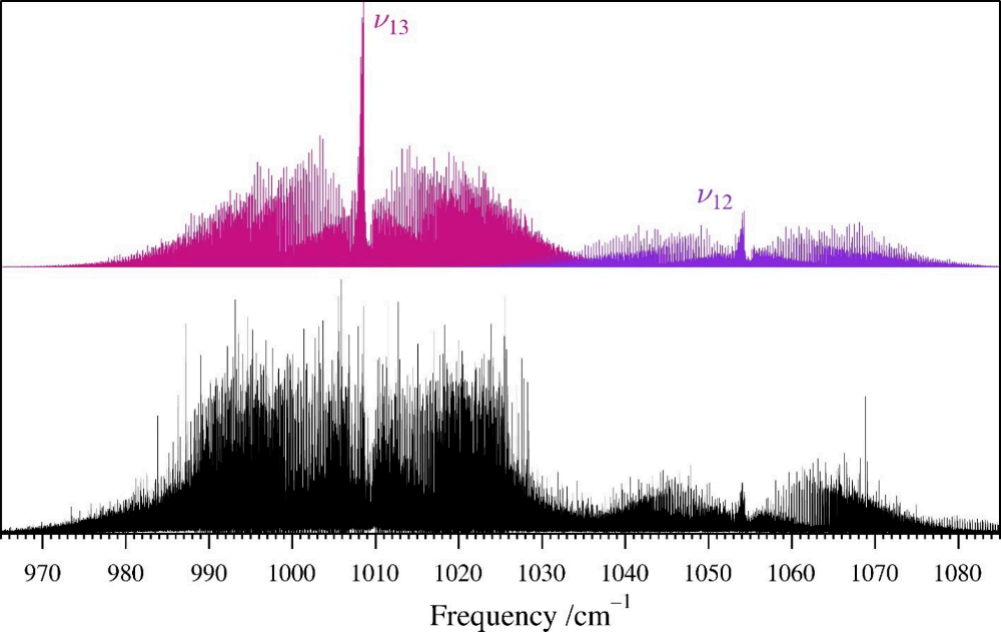
Pyrazole experimental high-resolution infrared spectrum (bottom) from 965 to 1085 cm^–1^ at a pressure of 18 mTorr and predicted stick *a*- and *b*-type spectra of ν_13_ and ν_12_ (light pink and light purple, respectively, top) from experimentally determined spectroscopic constants.

The available transitions of ν_13_ and ν_12_ have been fit to single-state, A-reduced Hamiltonians, in a similar fashion to the other vibrational states, albeit with relatively high error (σ_fit_ = 0.79 and 0.99 MHz, respectively) and inadequately modeled transitions. The data set included rotational transitions and IR transitions involving the fundamental and difference bands with ν_21_ in each case (Figure S37). Due to the unaddressed coupling, many transitions with confident assignments and *obs*. – *calc*. greater than three times the nominal measurement uncertainty were included in the least-squares fit, resulting in the large σ_fit_ values. To model the IR transitions, the constants of the ground state and ν_21_ were held at their previously well-determined values in the least-squares fit. The resulting spectroscopic constants obtained from the least-squares fits are provided in Table 6. These spectroscopic constants should be treated as highly effective with limited physical meaning. The least-squares fitting files are available in Supporting Information.

**Table 6:**
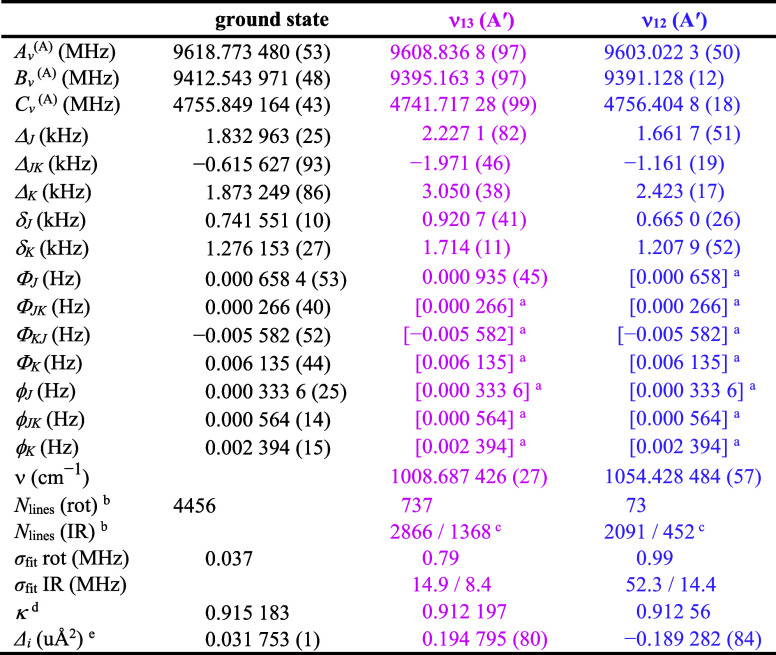
Spectroscopic Constants for the Ground, ν_13_, and ν_12_ Vibrational States of Pyrazole, A Reduction, I^*r*^ Representation

a Value held constant at the corresponding ground-state value.

b Number of independent transitions.

c The first value relates to transitions from the ground state; the second value relates to transitions from ν_21_.

d κ = (2*B* – *A* – *C*)/(*A* – *C*).

e Inertial defect (Δ_*i*_ *= I*_*c*_ – *I*_*a*_ – *I*_*b*_).

As further evidence of this intense coupling-induced mixing between these states, the 2ν_21_ – ν_21_ hot band could not be observed in the vicinity of ν_21_. Instead, two strong difference bands ν_13_ – ν_21_ and ν_12_ – ν_21_ are visible in Figure 6 at approximately 492 and 538 cm^–1^, respectively. Absent the Fermi coupling, 2ν_21_ – ν_21_ would be the most intense hot band in the vicinity of ν_21_ due to Boltzmann factors. In this case, however, ν_13_ and ν_12_ have taken on sufficient 2ν_21_ character from state-mixing that normally weak difference bands become quite strong (Figure S37). These transitions of ν_13_ – ν_21_ and ν_12_ – ν_21_ were included in the data set, providing redundant information regarding the spectroscopic constants and vibrational energies of these states.

The impact of the untreated Coriolis and Fermi coupling is clear in the vibration-rotation interaction constants of both ν_13_ and ν_12_ (Table 3). There is rather poor agreement between the computed and experimental constants, with neither set of constants likely free of perturbation. Additionally, the quartic centrifugal distortion constants (Table 6) have larger deviations from their ground-state counterparts for both states than the lower-energy fundamental states, another indication of the untreated coupling. Attempts to address the observed resonances in ν_13_ and ν_12_ by manually adjusting Coriolis and Fermi coupling terms while maintaining the assumed 2ν_21_ – ν_21_ band origin were not successful at predicting and assigning the IR spectrum of 2ν_21_ or producing more physically meaningful spectroscopic constants of ν_13_ and ν_12_. Likewise, our attempts to assign IR transitions to the remaining hot band were unsuccessful. The most intense remaining unassigned hot band in the vicinity of ν_21_ (Figure 6, 519 cm^–1^) is most likely not 2ν_21_ – ν_21_. By analysis of the unassigned hot band frequencies, this feature has been tentatively (though not conclusively) assigned to ν_21_ + ν_17_ – ν_21_. Attempts to locate unassigned *K*_*a*_ series of rotational transitions or unassigned P-branch or R-branch IR transitions using the predicted spectroscopic constants have been unsuccessful, preventing a multistate treatment of this coupled triad. The most valuable and reliable information obtained from the analyses of these vibrational states is the determination of their vibrational energies.

### Rotational and IR Spectra of ν_11_, ν_21_ + ν_20_, ν_10_, ν_21_ + ν_19_, and ν_21_ + ν_18_

The highest-energy fundamental state for which transitions have been observed (Figure 5), measured, and least-squares fit states is ν_11_ (A′, 1121.060 371 7 (57) cm^–1^). Similar to ν_13_ and ν_12_, the rotational transitions of ν_11_ could not be assigned using the ground-state spectroscopic constants and vibration-rotation interaction constants. Initial fitting of the IR transitions allowed the rotational transitions of ν_11_ to be assigned and included in the data set for least-squares fitting. While ν_10_ (A′) is lower in energy than combination states ν_21_ + ν_19_ and ν_21_ + ν_18_^,^ for which rotational transitions have been observed, its rotational transitions were too weak or too perturbed by coupling interactions to identify without very accurate predictions from fitting the IR data. The band origin of fundamental ν_10_ is tentatively assigned to a weak band with a Q-branch at 1184 cm^–1^ (Figure 12), which is about 25 cm^–1^ higher than its previous assignment from solid-phase Raman spectroscopy.^28^ The intensity of ν_10_ is insufficient in the available data to measure and assign any of its IR transitions, preventing further analysis of this state. The infrared and rotational transitions of ν_11_ and ν_10_ are perturbed by the near-energy A′ combination states ν_21_ + ν_20_ (1134.850 562 2 (60) cm^–1^), ν_21_ + ν_19_ (1185.053 561 0 (37) cm^–1^), and ν_21_ + ν_18_ (1263.475 638 (12) cm^–1^) by complex Fermi and Coriolis couplings. All of these combination states were first identified by their hot bands involving ν_21_, ν_20_, ν_19_, and ν_18_, using predictions made from extrapolations of the ground state and corresponding fundamental states. Figures 6 and 7 highlight all the hot-band IR transitions that were observable and included in the data set. The rotational transitions of all of these vibrationally excited states were assigned and measured once preliminary least-squares fits of the IR data of the hot bands were obtained. Due the low intensity of these rotational transitions and the resulting insufficient spectroscopic information on all states in this polyad, a multistate fit was not attempted. The milder interactions between these states, compared to those of ν_13_ and ν_12_, allowed effective fits to be obtained by excluding transitions with *obs*. – *calc*. > 5. The resulting spectroscopic constants obtained from the least-squares fits are provided in Table 7. The larger number of mildly perturbed transitions allowed these effective fits to produce spectroscopic constants that deviated only slightly from the ground state and are likely to be more physically meaningful than those of ν_13_ and ν_12_. The vibration-rotation interaction constants for ν_11_ displayed in Table 3 provide further support for this assertion. Even with the unaddressed couplings, the computed and experimental ν_11_ vibration-rotation interaction constants are in much closer agreement than those of ν_13_ and ν_12_. A comparison of the measured and extrapolated spectroscopic constants for ν_21_ + ν_20_, ν_21_ + ν_19_, and ν_21_ + ν_18_ (Table S3) provides confirmation that all of the quartic centrifugal distortion constants are of the expected order of magnitude and sign. Despite the reasonable spectroscopic parameters obtained for ν_11_, ν_21_ + ν_20_, ν_21_ + ν_19_, and ν_21_ + ν_18_, their constants should be considered effective.

**Table 7:**
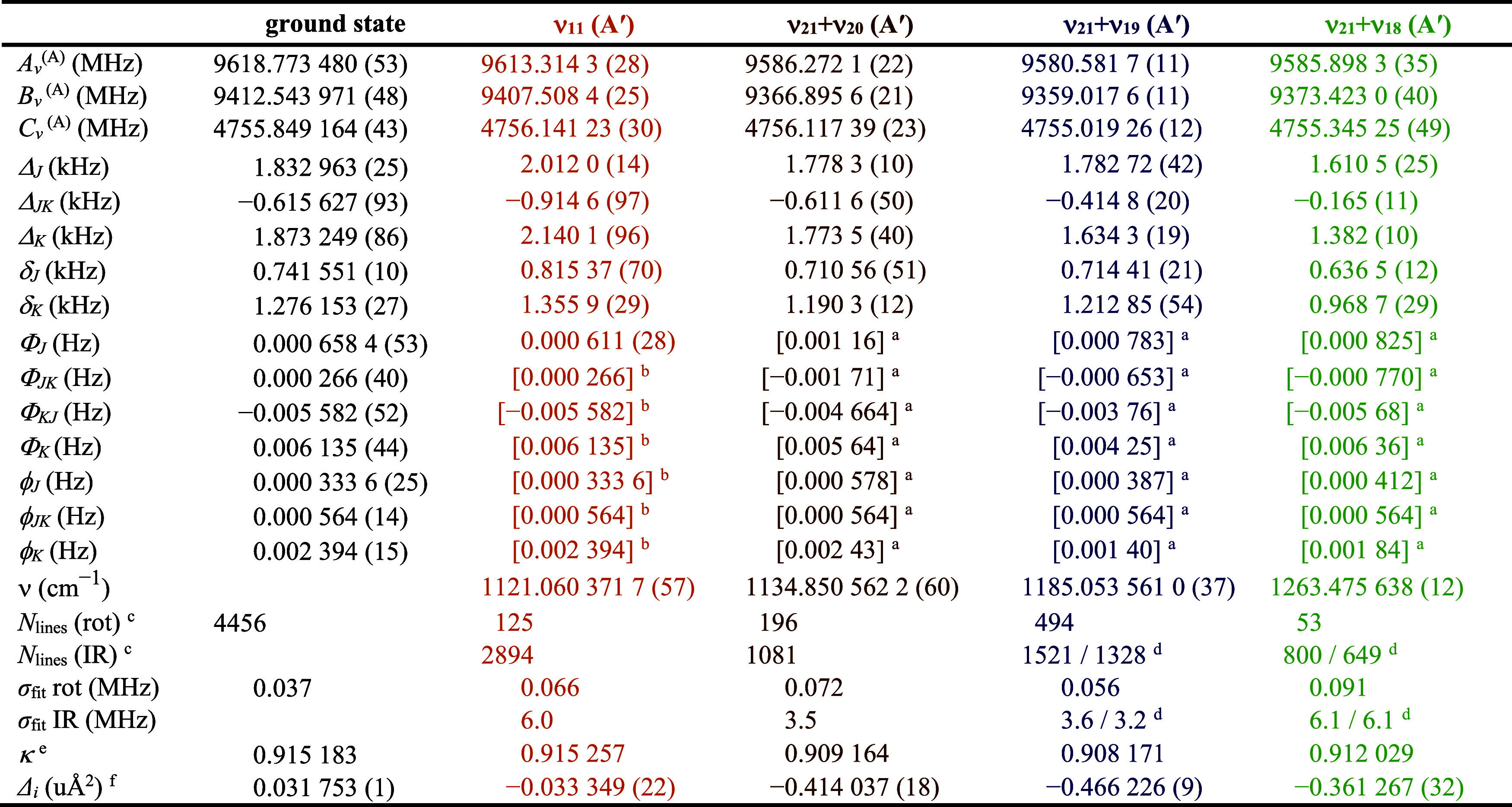
Spectroscopic Constants for the Ground, ν_11_, ν_21_ + ν_20_, ν_21_ + ν_19_, and ν_21_ + ν_18_ Vibrational States of Pyrazole, A Reduction, I^*r*^ Representation

a Value held constant at the extrapolated value.

b Value held constant at the corresponding ground-state value.

c Number of independent transitions.

d The first value relates to transitions between the combination state and ν_21_; the second value relates to transitions between the combination state and other fundamental state.

e κ = (2*B* – *A* – *C*)/(*A* – *C*).

f Inertial defect (*Δ*_*i*_ *= I*_*c*_ – *I*_*a*_ – *I*_*b*_).

**Figure 12:**
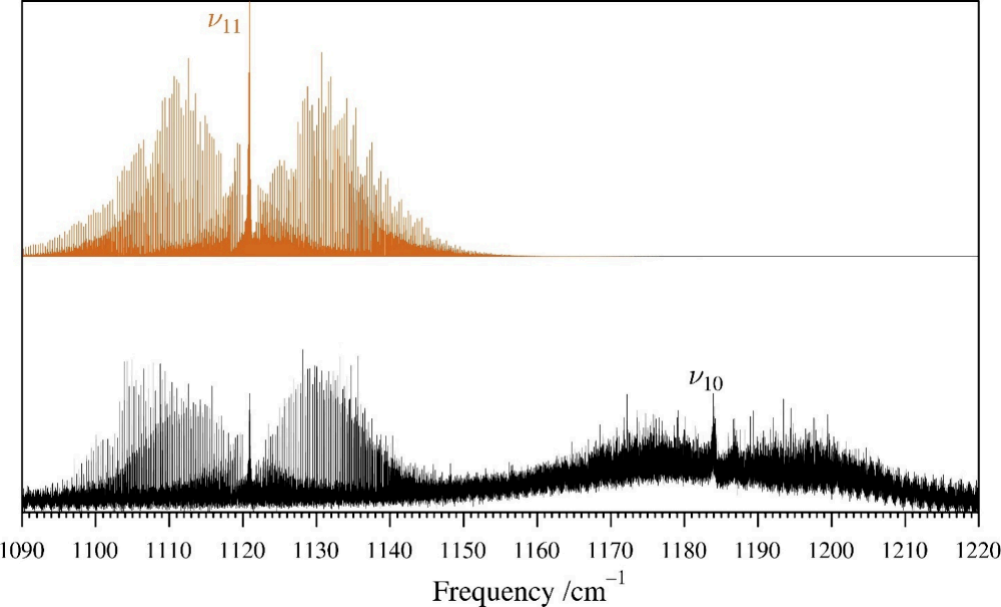
Pyrazole experimental high-resolution infrared spectrum (bottom) from 1090 to 1220 cm^–1^ at a pressure of 9 mTorr and predicted stick *a*- and *b*-type spectra of the ν_11_ band (light brown, top) from experimentally determined spectroscopic constants. The transitions of ν_10_ are not resolvable, measurable, and assignable at the pressure of data collection due to the signal-to-noise ratio.

### Comparison of Computed and Experimental Vibrational Frequencies

The analysis of the high-resolution IR bands in this work, summarized in Figure S38, allows the highly precise and accurate vibrational energies of 14 different states to be determined. Table 8 provides a direct comparison between the vibrational energies determined in this work with their corresponding low-resolution solid-phase,^28^ B3LYP, and CCSD­(T) values; predicted infrared intensities of the observed bands are provided in Table S4. Unsurprisingly, the CCSD­(T) fundamental frequencies agree better with the experimental values than the B3LYP values do for the fundamental states that are either adequately treated by single-state models or as part of the tetrad (except ν_21_). For pyrazole, either computational method predicts the frequencies to within 12 cm^–1^, which is sufficient to unambiguously assign the IR bands for these states. Interestingly, the CCSD­(T) *obs*. – *calc*. value (11.9 cm^–1^) for ν_21_, the out-of-plane N–H wag of pyrazole, is nearly identical to the CCSD­(T) *obs*. – *calc*. value for the analogous out-of-plane N–H wag of 1*H*-1,2,4-triazole (11.9 cm^–1^).^7^ In contrast, however, the CCSD­(T) *obs*. – *calc*. value for the out-of-plane N–H wags of 1*H*-1,2,3-triazole and 2*H*-1,2,3-triazole are 0.11 cm^–1^ and 0.19 cm^–1^, respectively.^9^ The structural similarity of pyrazole and 1*H*-1,2,4-triazole suggests the possibility of a systematic error in the calculation for this type of vibrational motion, where the N–H group is between sp^2^-hybridized carbon and nitrogen atoms. The precise origin of the anomalous N–H wag frequencies of pyrazole and 1*H*-1,2,4-triazole are unknown at this time. The remaining fundamental and combination frequencies (> 1000 cm^–1^) of pyrazole are for states involved in unaddressed anharmonic couplings in the anharmonic frequency calculations and are thus not expected to show the same level of agreement between theory and experiment.

**Table 8:**
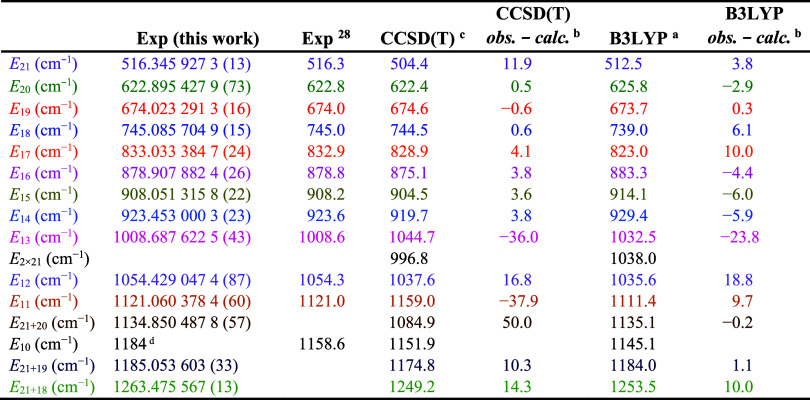
Gas-Phase Experimental and Computed Vibrational Energies of Pyrazole

a Evaluated with the 6-311G+(2d,p) basis set.

b Observed values are those determined in this work.

c Evaluated with the cc-pCVTZ basis set.

d Tentative assignment based upon visual estimation of the band center.

## Conclusions

We provide an analysis of the rotational spectrum of 1*H*-pyrazole up to 750 GHz, well beyond the previous 205 GHz, providing over 4400 transition frequencies and a complete set of spectroscopic constants up to the sextic level of centrifugal distortion for the ground vibrational state in the A reduction, I^*r*^ representation. The extensive coverage of *J* and *K* values in the transition data set and well-determined spectroscopic constants provide confidence that they could be used to accurately predict transition frequencies above 750 GHz. The high-resolution infrared spectrum of 14 vibrationally excited states lying below 1300 cm^–1^ of 1*H*-pyrazole are measured for the first time, which, combined with rotational transitions, provide a large number of well-determined spectroscopic constants and fundamental frequencies of 1*H*-pyrazole. The analysis of the vibrationally excited states would not have been as successful without the combination of rotational and high-resolution IR spectra. It would have been very difficult, for example, to assign the IR spectrum of ν_20_ without previously analyzing the rotational spectrum. Likewise, it would have been very difficult to analyze the rotational spectrum of ν_16_, ν_15_, and ν_14_ without spectroscopic constants determined from the high-resolution IR data.

The spectroscopic analysis of 1*H*-pyrazole could be improved substantially by assigning rotational transitions of 2ν_21_ or infrared transitions of 2ν_21_ – ν_21_. Without both the vibrational energy of 2ν_21_ from the IR data and the highly perturbed transitions in the rotational spectrum, it will be difficult to address the coupling observed in ν_12_ and ν_13_ and obtain satisfactory least-squares fits for any of these three states. Locating the rotational transitions of 2ν_21_ will likely require better computational predictions of the coupling interactions or fitting of the 2ν_21_ – ν_21_ IR band to provide better initial spectroscopic constants of 2ν_21_. As described previously, the state-mixing between 2ν_21_ and ν_13_ and ν_12_ allows the fundamentals to take on sufficient 2ν_21_ character to cause the difference bands (ν_13_ – ν_21_ and ν_12_ – ν_21_) to be much more intense than they would be absent the coupling interactions. This intensity borrowing diminishes the 2ν_21_ – ν_21_ band intensity and would likely require the reacquisition of the high-resolution IR data at a higher pressure to improve the sensitivity and enable determination of the 2ν_21_ band origin.

This work showcases the synergy of millimeter-wave rotational spectroscopy and high-resolution infrared spectroscopy. These techniques provide redundant spectroscopic information with respect to the rotational energy levels over a similar range of *J* and *K*_*a*_ values. As described above, infrared spectroscopy provides unambiguous vibrational state assignments due to the vibrational energy. Rotational spectroscopy provides more precise measurements of the transition frequencies and provides access to vibrationally excited-state transitions whose intensity is based upon observation temperature and not the vibrational transition dipole. Analysis of the vibrationally excited states in this work required access to data sets from both frequency regions.

## Supplementary Material





## Notes

Data distribution plots, spectroscopic constants from alternate least-squares fits, Loomis–Wood plots, resonance plots, and energy level diagrams (PDF)
Computational output files and least-squares fitting files (ZIP)
