# Supplementary material for: Millimeter-Wave and High-Resolution Infrared Spectroscopy of the Ground and 14 Vibrationally Excited States Lying Below 1300 cm–1 of Pyrazole
Source: J Phys Chem A. 2025 Oct 29;129(44):10213–27. doi: 10.1021/acs.jpca.5c06339 (PMC12598870; doi:10.1021/acs.jpca.5c06339)
Supplement: Supplementary file 1 [file jp5c06339_si_001.pdf]

Supporting Information

Millimeter-Wave and High-Resolution Infrared Spectroscopy of the Ground and Fourteen Vibrationally Excited States Lying Below 1300 cm^{-1} of Pyrazole

Brian J. Esselman[1,*], Maria A. Zdanovskaia[1],
Jeff G. Crouse[2], Doyeon Kim[2], Brant E. Billinghurst[3],
Dennis W. Tokaryk[2], R. Claude Woods[1], Robert J. McMahon[1]

[1] *Department of Chemistry, University of Wisconsin−Madison, 1101 University Avenue, Madison, Wisconsin 53706-1322, United States*
[2] *Department of Physics, University of New Brunswick, P.O. Box 4400, Fredericton, New Brunswick E3B 5A3, Canada*
[3] *Canadian Light Source. Inc., University of Saskatchewan, Saskatoon, SK S7N 2V3, Canada*

* corresponding author
E-mail address: brian.esselman@wisc.edu (B.J. Esselman).

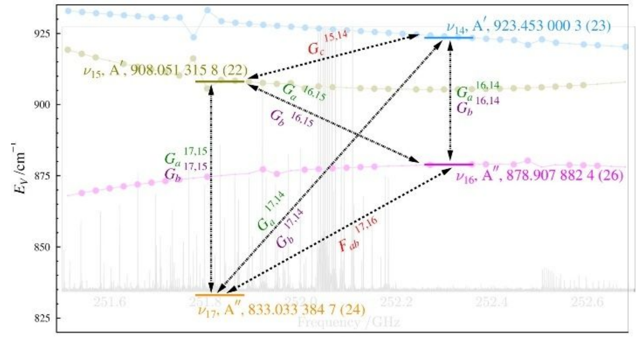

Table of Contents	Page
**Figures S1 – S32.** Data distribution plots for pyrazole vibrationally excited states	S3 - S34
**Figure S33.** Loomis-Wood plot focused on a *c*-type P-branch series (K_c = 9) of ν_{20} ← g.s. of pyrazole	S35
**Figure S34.** Resonance progression plot for ν_{14}	S36
**Figure S35.** Nominal interstate diagram	S37
**Figure S36.** Loomis-Wood plot of an *a*-type R-branch series (K_a = 5) of ν_{12} ← g.s. of pyrazole	S38
**Figure S37.** Energy diagram representing the coupling interactions of $2\nu_{21}$, ν_{12}, and ν_{13}	S38
**Figure S38.** Energy level diagram showing the observed IR bands in this work	S38
**Table S1.** Spectroscopic constants for the ground vibrational state of pyrazole (S reduction, III^r representation). (Includes B3LYP computed values)	S40
**Table S2.** Spectroscopic constants for the ground and ν_{21} vibrational states of pyrazole with Φ_{JK} of ν_{21} held constant and allowed to fit (A reduction, I^r representation)	S41
**Table S3.** Extrapolated and fit spectroscopic constants (A Reduction, I^r Representation) for assigned combination states of pyrazole.	S41
**Table S4.** Computed infrared intensities.	S43
**References**	S43
Least-squares fitting output file for ground vibrational state (pyrazole_gs.res)	Separate file
Least-squares fitting output file for higher-energy vibrational states (.res files)	Separate file
CCSD(T)/cc-pCVTZ anharmonic outputs for pyrazole	Separate file
B3LYP/ 6-311+G(2d,p) anharmonic output for pyrazole	Separate file

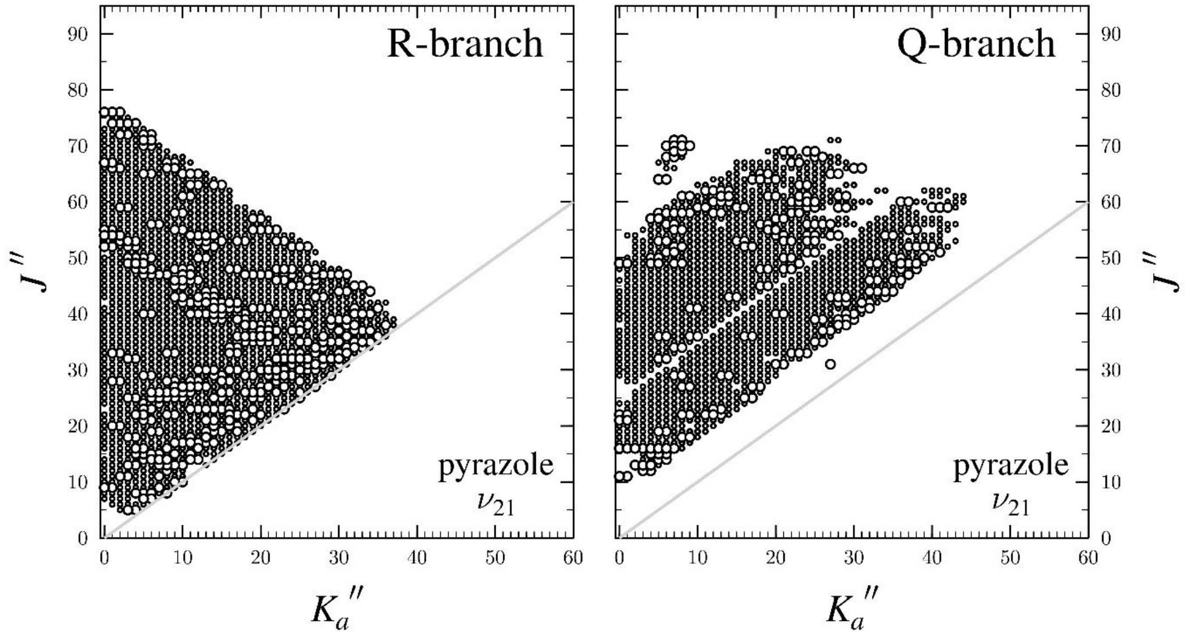

Figure S1. Data distribution plot for the least-squares fit of millimeter-wave spectroscopic data for the normal isotopologue of pyrazole, ν_{21}. The size of the outlined circle is proportional to the value of $|(f_{obs.} - f_{calc.})/\delta f|$, where δf is the frequency measurement uncertainty, and no measurements have a quotient value larger than 3.

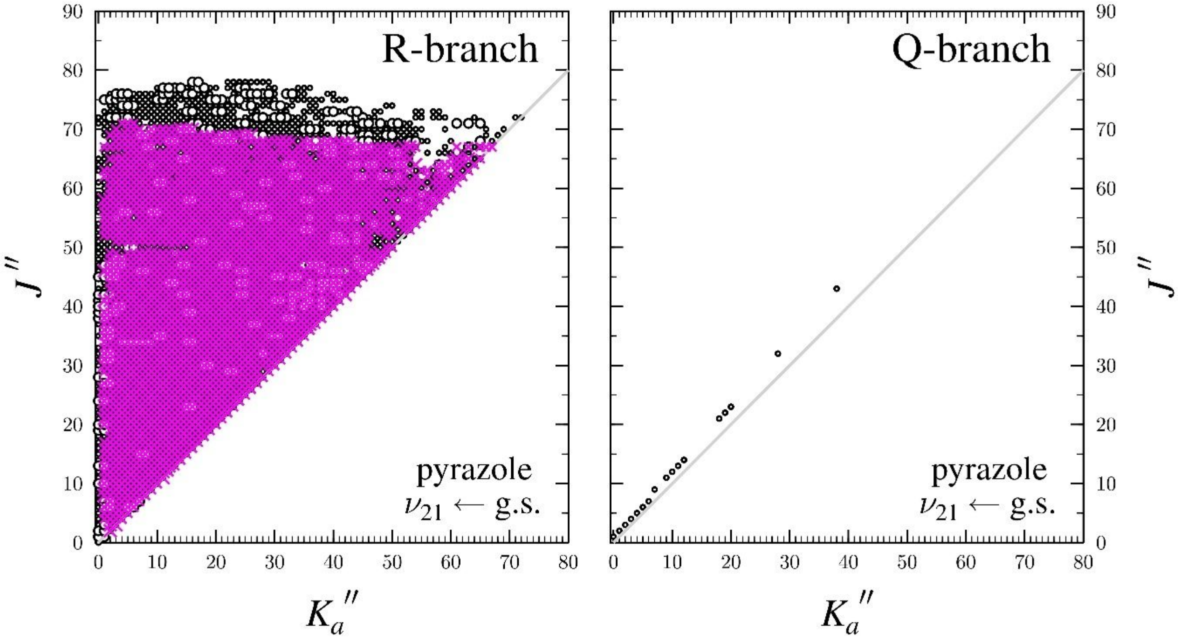

Figure S2. Data distribution plot for the least-squares fit of high-resolution infrared spectroscopic data for the normal isotopologue of pyrazole, $n_{21} \leftarrow$ g.s. Circles indicate R- and Q-branch transitions, while magenta crosses indicate P-branch transitions. The size of the symbol is proportional to the value of $|(f_{obs.} - f_{calc.})/\delta f|$, where δf is the frequency measurement uncertainty, and no measurements have a quotient value larger than 3.

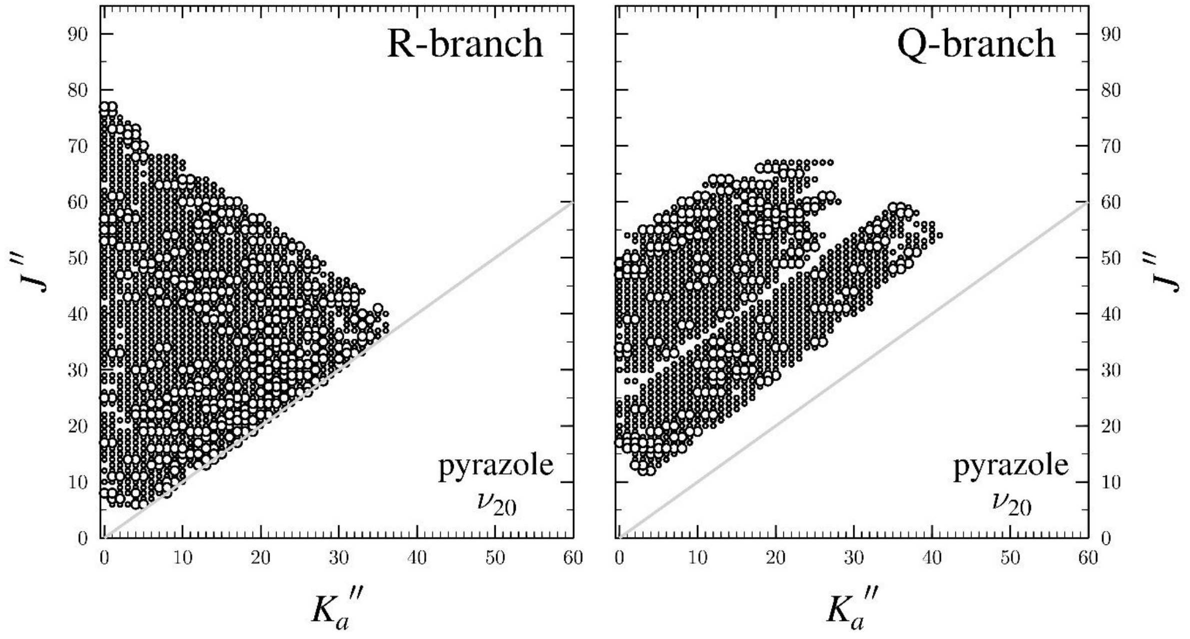

Figure S3. Data distribution plot for the least-squares fit of millimeter-wave spectroscopic data for the normal isotopologue of pyrazole, ν_{20}. The size of the outlined circle is proportional to the value of $|(f_{obs.} - f_{calc.})/\delta f|$, where δf is the frequency measurement uncertainty, and no measurements have a quotient value larger than 3.

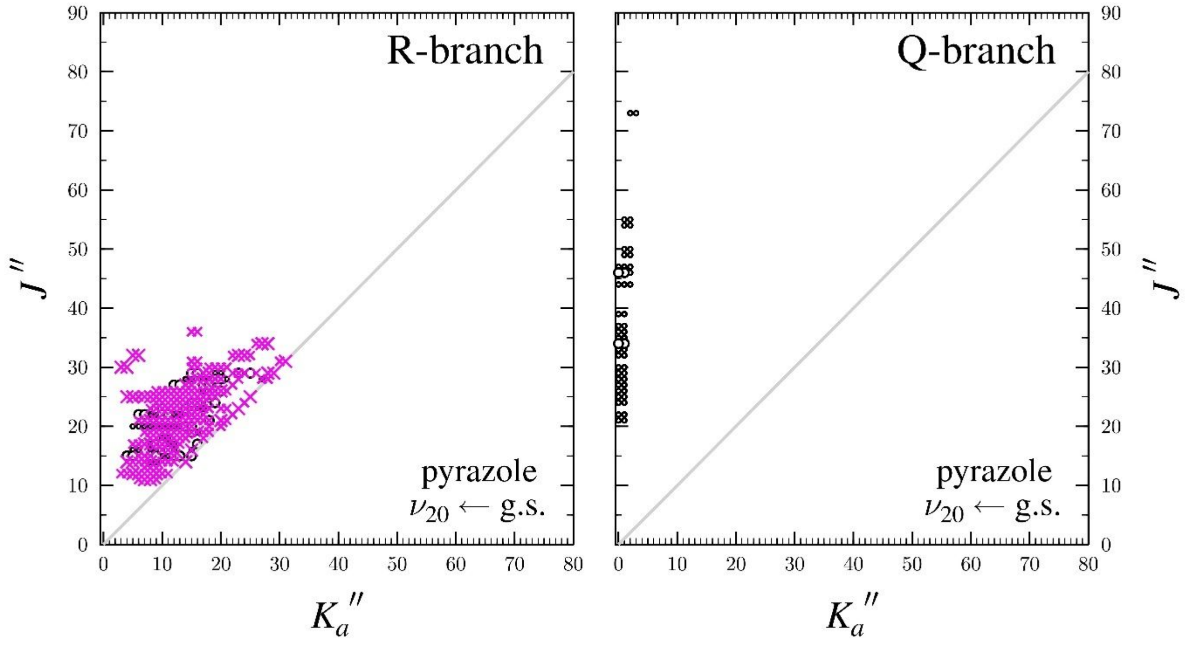

Figure S4. Data distribution plot for the least-squares fit of high-resolution infrared spectroscopic data for the normal isotopologue of pyrazole, $\nu_{20} \leftarrow$ g.s. Circles indicate R- and Q-branch transitions, while magenta crosses indicate P-branch transitions. The size of the symbol is proportional to the value of $|(f_{obs.} - f_{calc.})/\delta f|$, where δf is the frequency measurement uncertainty, and no measurements have a quotient value larger than 3.

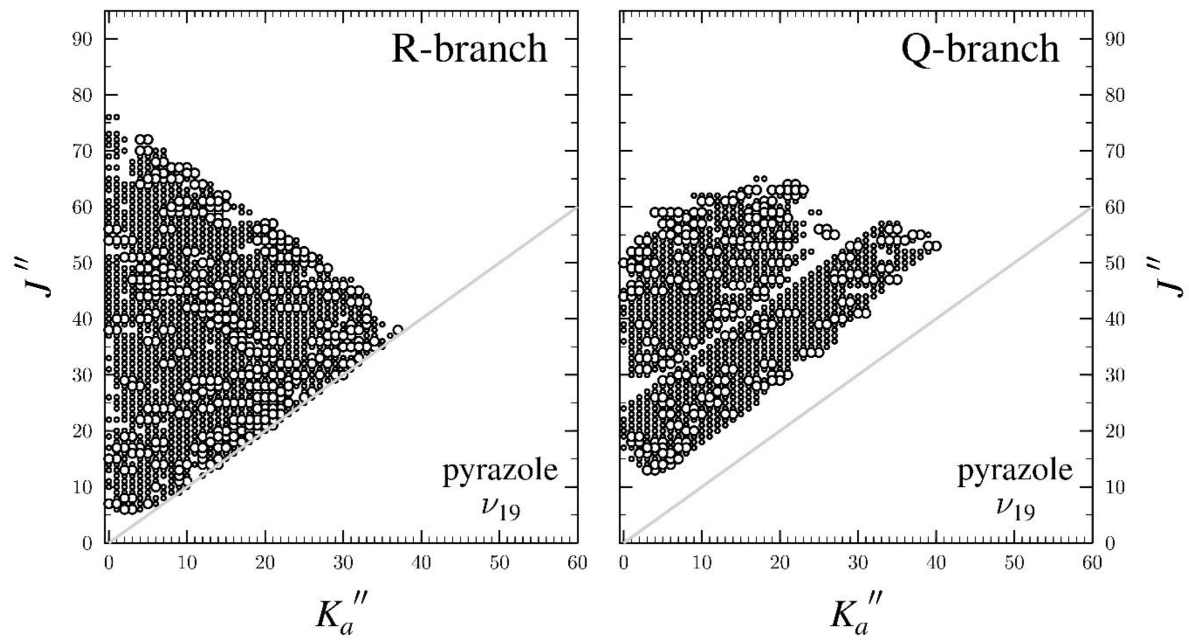

Figure S5. Data distribution plot for the least-squares fit of millimeter-wave spectroscopic data for the normal isotopologue of pyrazole, ν_{19}. The size of the outlined circle is proportional to the value of $|(f_{obs.} - f_{calc.})/\delta f|$, where δf is the frequency measurement uncertainty, and no measurements have a quotient value larger than 3.

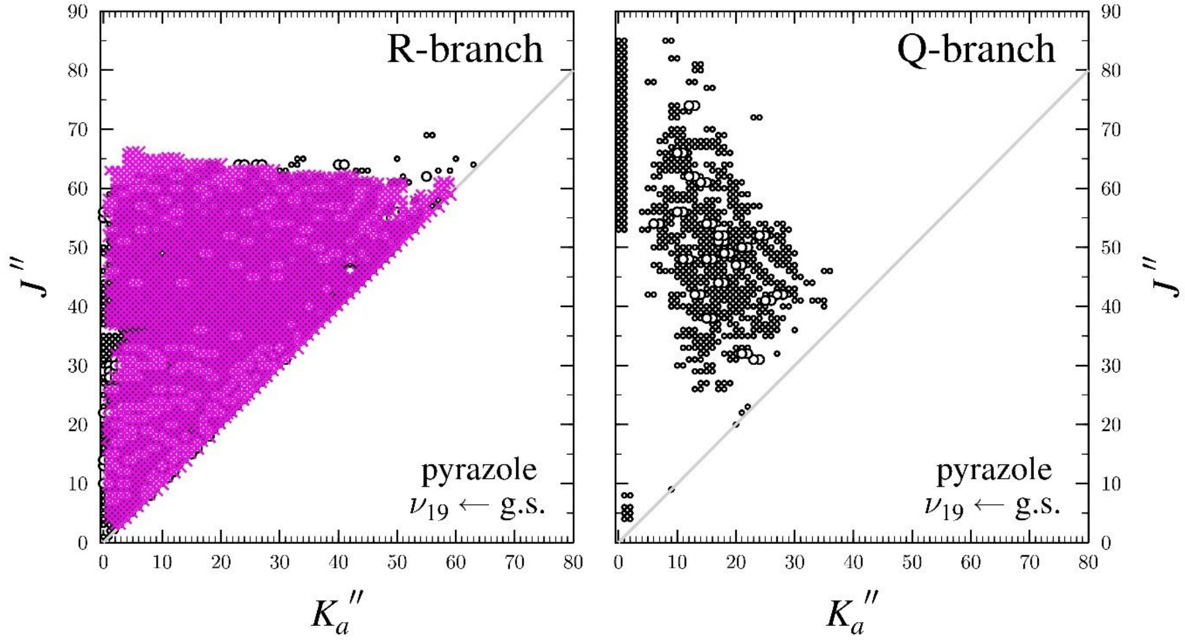

Figure S6. Data distribution plot for the least-squares fit of high-resolution infrared spectroscopic data for the normal isotopologue of pyrazole, $\nu_{19} \leftarrow$ g.s. Circles indicate R- and Q-branch transitions, while magenta crosses indicate P-branch transitions. The size of the symbol is proportional to the value of $|(f_{obs.} - f_{calc.})/\delta f|$, where δf is the frequency measurement uncertainty, and no measurements have a quotient value larger than 3.

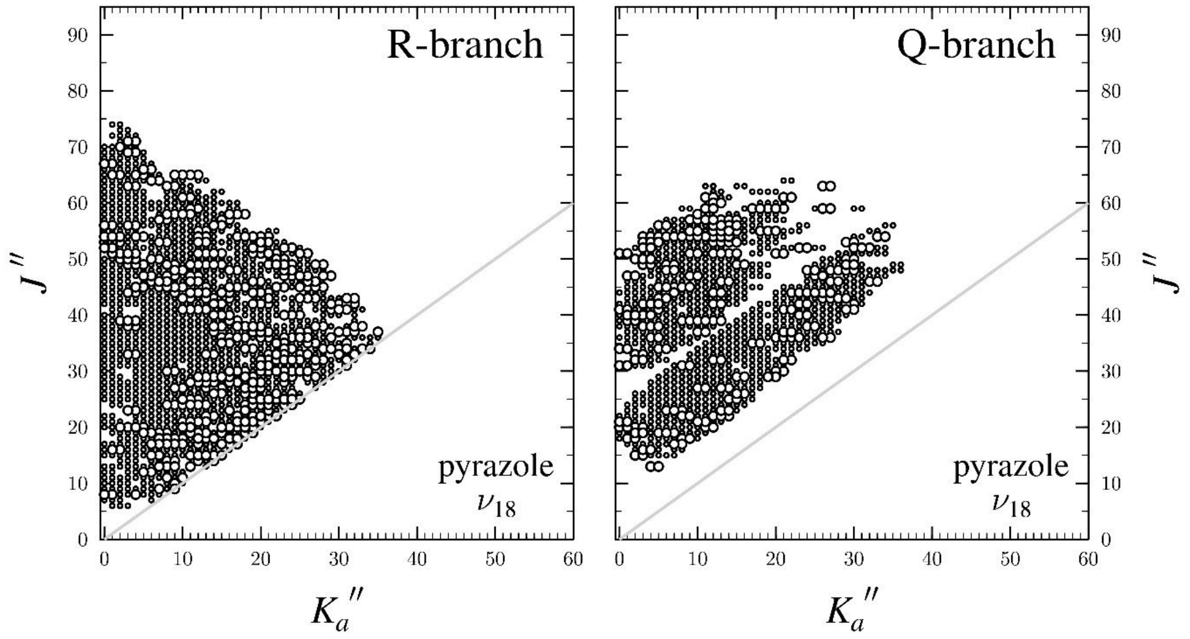

Figure S7. Data distribution plot for the least-squares fit of millimeter-wave spectroscopic data for the normal isotopologue of pyrazole, ν_{18}. The size of the outlined circle is proportional to the value of $|(f_{obs.} - f_{calc.})/\delta f|$, where δf is the frequency measurement uncertainty, and no measurements have a quotient value larger than 3.

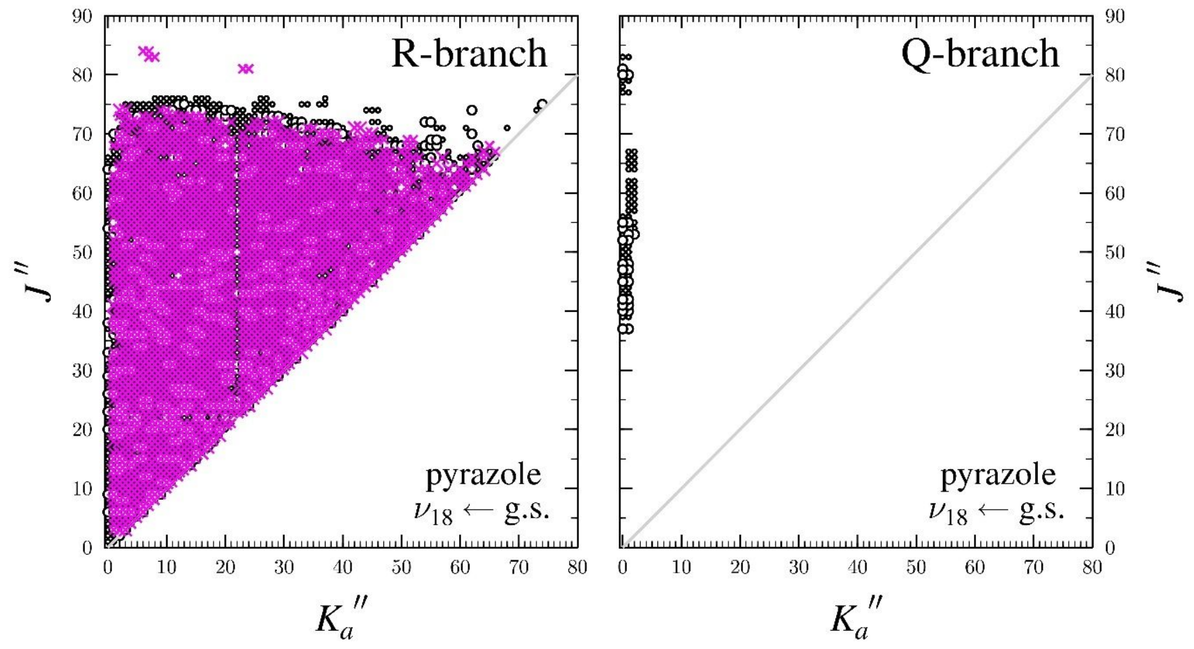

Figure S8. Data distribution plot for the least-squares fit of high-resolution infrared spectroscopic data for the normal isotopologue of pyrazole, $\nu_{18} \leftarrow$ g.s. Circles indicate R- and Q-branch transitions, while magenta crosses indicate P-branch transitions. The size of the symbol is proportional to the value of $|(f_{obs.} - f_{calc.})/\delta f|$, where δf is the frequency measurement uncertainty, and no measurements have a quotient value larger than 3.

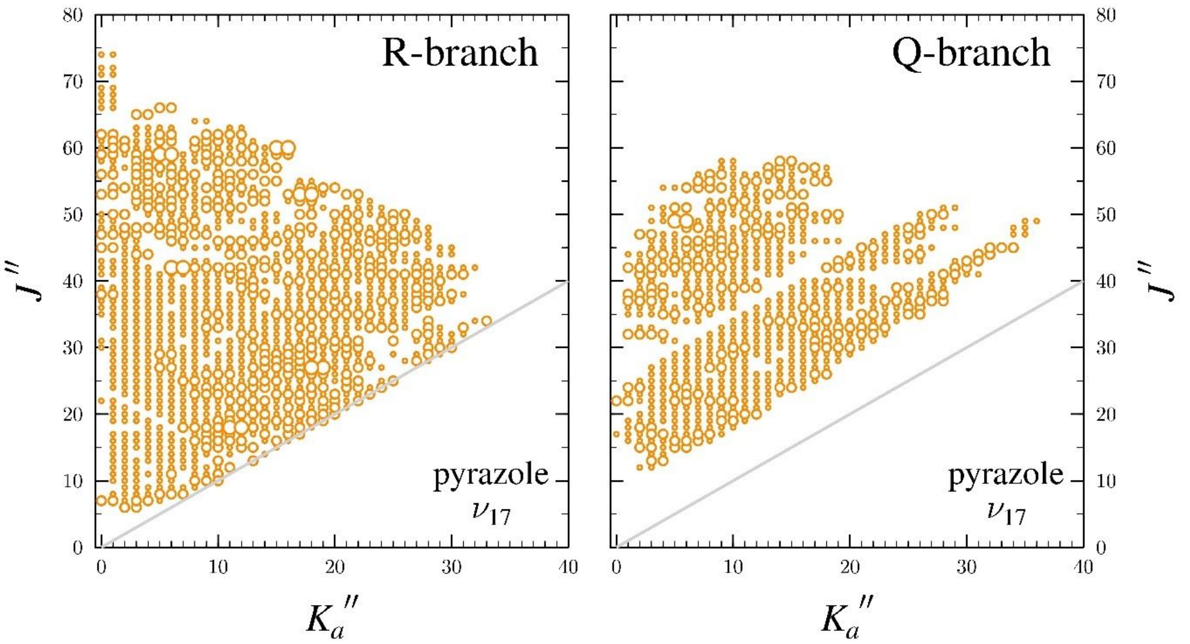

Figure S9. Data distribution plot for the least-squares fit of millimeter-wave spectroscopic data for the normal isotopologue of pyrazole, ν_{17}. The size of the outlined circle is proportional to the value of $|(f_{obs.} - f_{calc.})/\delta f|$, where δf is the frequency measurement uncertainty, and no measurements have a quotient value larger than 3.

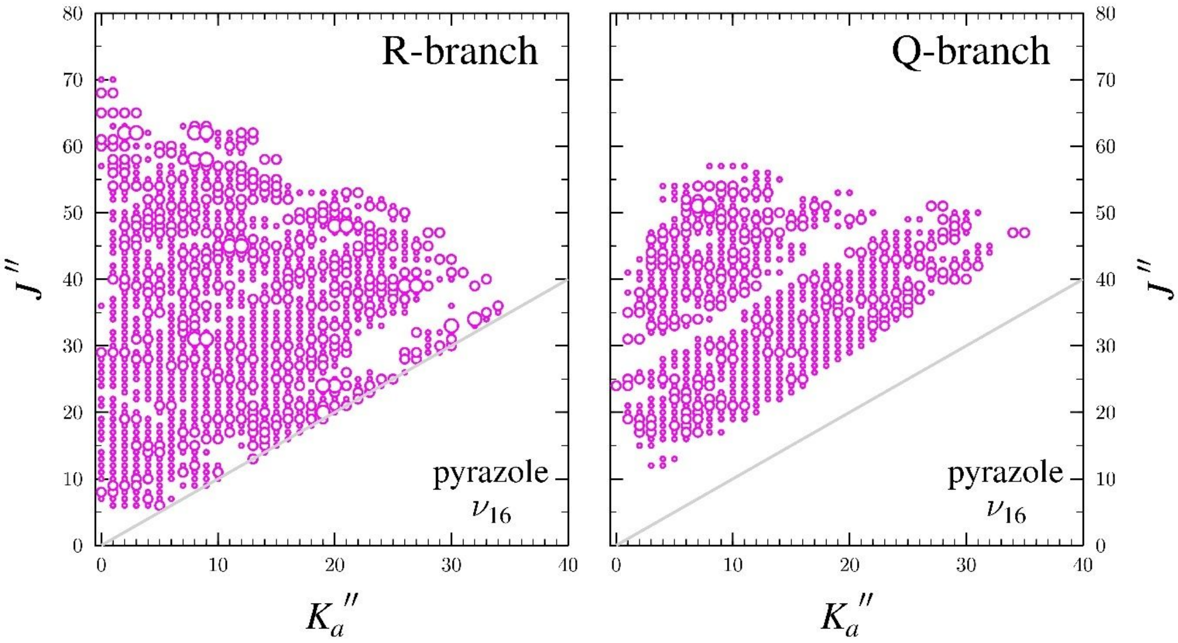

Figure S10. Data distribution plot for the least-squares fit of millimeter-wave spectroscopic data for the normal isotopologue of pyrazole, ν_{16}. The size of the outlined circle is proportional to the value of $|(f_{obs.} - f_{calc.})/\delta f|$, where δf is the frequency measurement uncertainty, and no measurements have a quotient value larger than 3.

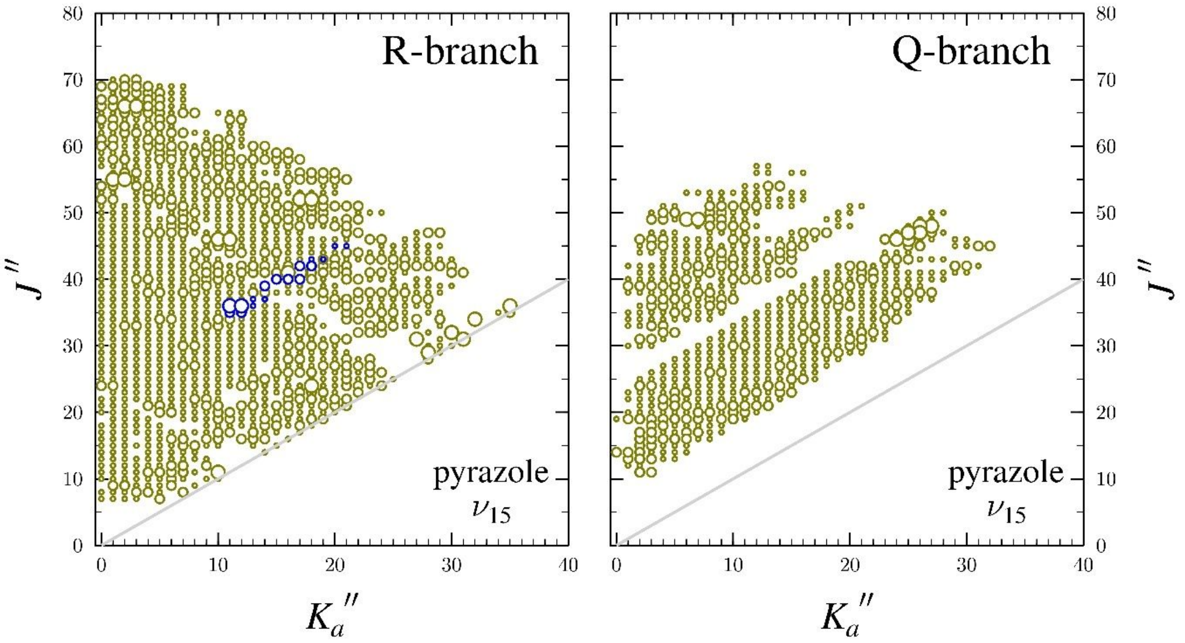

Figure S11. Data distribution plot for the least-squares fit of millimeter-wave spectroscopic data for the normal isotopologue of pyrazole, ν_{15}. Blue circles indicate nominal interstate transitions to ν_{14}. The size of the outlined circle is proportional to the value of $|(f_{obs.} - f_{calc.})/\delta f|$, where δf is the frequency measurement uncertainty, and no measurements have a quotient value larger than 3.

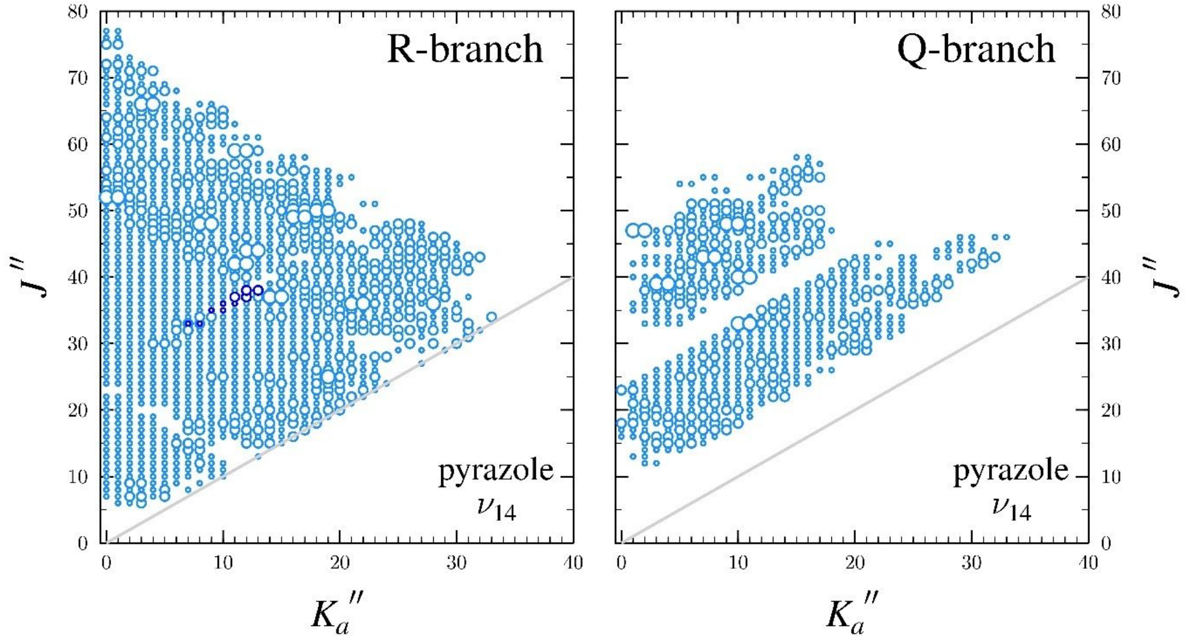

Figure S12. Data distribution plot for the least-squares fit of millimeter-wave spectroscopic data for the normal isotopologue of pyrazole, ν_{14}. Blue circles indicate nominal interstate transitions to ν_{15}. The size of the outlined circle is proportional to the value of $|(f_{obs.} - f_{calc.})/\delta f|$, where δf is the frequency measurement uncertainty, and no measurements have a quotient value larger than 3.

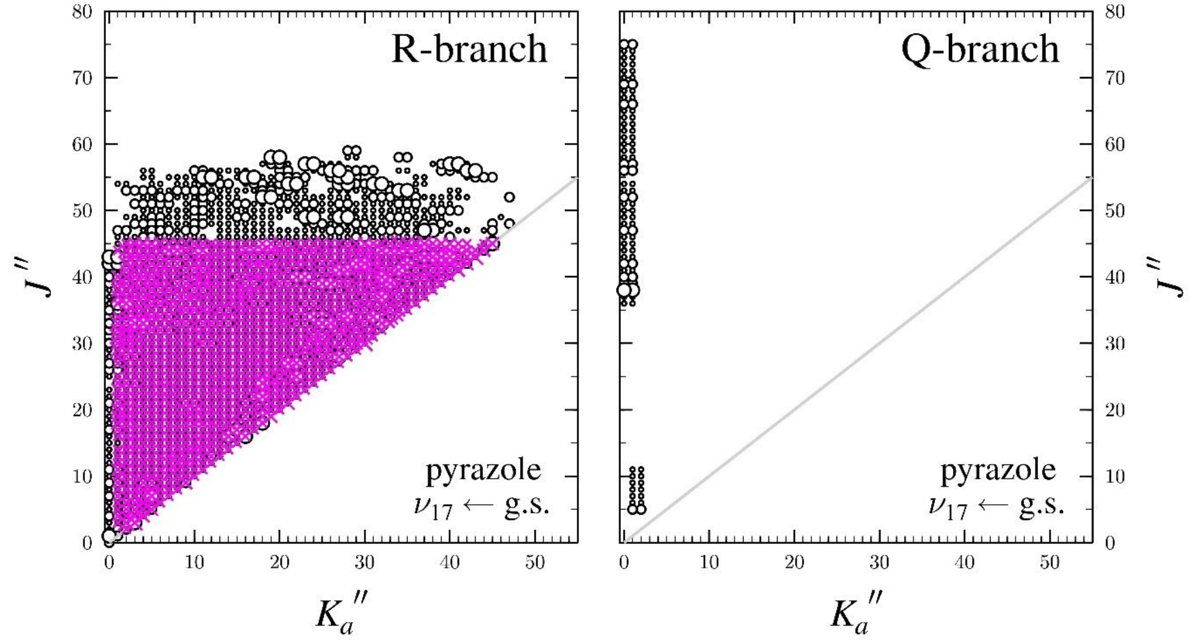

Figure S13. Data distribution plot for the least-squares fit of high-resolution infrared spectroscopic data for the normal isotopologue of pyrazole, $\nu_{17} \leftarrow$ g.s. Circles indicate R- and Q-branch transitions, while magenta crosses indicate P-branch transitions. The size of the symbol is proportional to the value of $|(f_{obs.} - f_{calc.})/\delta f|$, where δf is the frequency measurement uncertainty, and no measurements have a quotient value larger than 3.

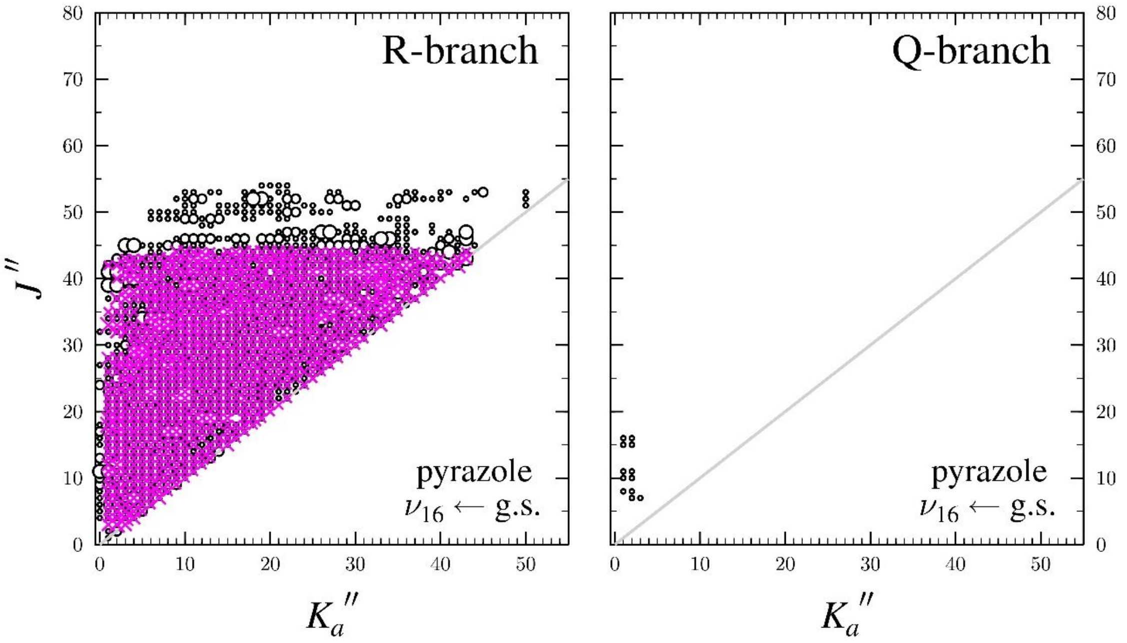

Figure S14. Data distribution plot for the least-squares fit of high-resolution infrared spectroscopic data for the normal isotopologue of pyrazole, $\nu_{16} \leftarrow$ g.s. Circles indicate R- and Q-branch transitions, while magenta crosses indicate P-branch transitions. The size of the symbol is proportional to the value of $|(f_{obs.} - f_{calc.})/\delta f|$, where δf is the frequency measurement uncertainty, and no measurements have a quotient value larger than 3.

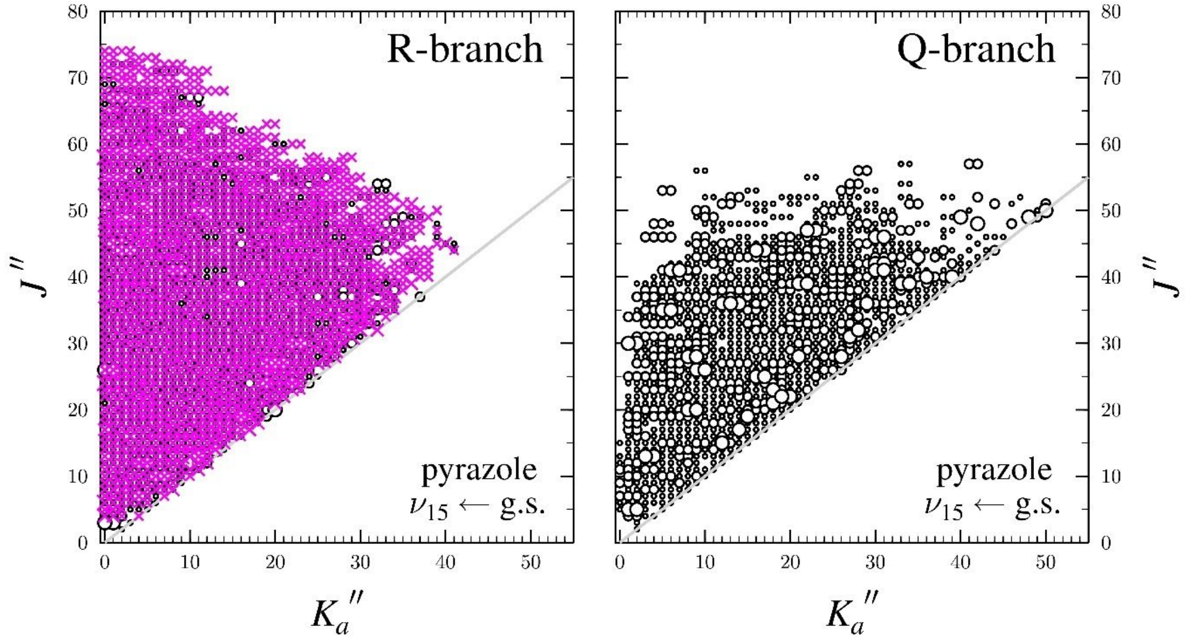

Figure S15. Data distribution plot for the least-squares fit of high-resolution infrared spectroscopic data for the normal isotopologue of pyrazole, $\nu_{15} \leftarrow$ g.s. Circles indicate R- and Q-branch transitions, while magenta crosses indicate P-branch transitions. The size of the symbol is proportional to the value of $|(f_{obs.} - f_{calc.})/\delta f|$, where δf is the frequency measurement uncertainty, and no measurements have a quotient value larger than 3.

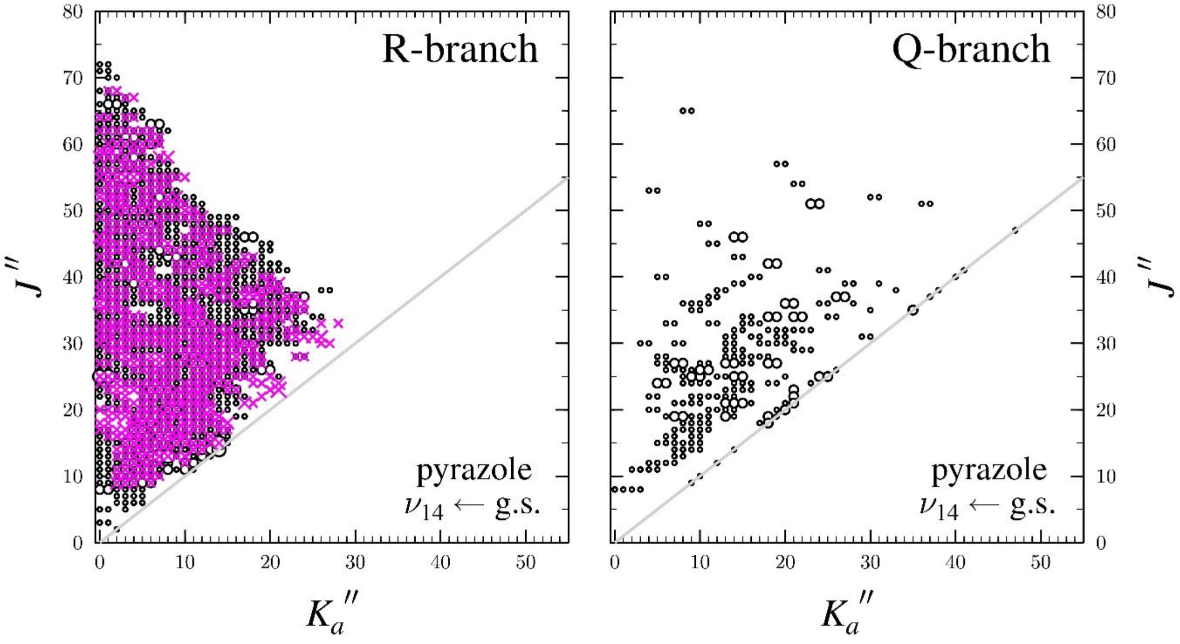

Figure S16. Data distribution plot for the least-squares fit of high-resolution infrared spectroscopic data for the normal isotopologue of pyrazole, $\nu_{14} \leftarrow$ g.s. Circles indicate R- and Q-branch transitions, while magenta crosses indicate P-branch transitions. The size of the symbol is proportional to the value of $|(f_{obs.} - f_{calc.})/\delta f|$, where δf is the frequency measurement uncertainty, and no measurements have a quotient value larger than 3.

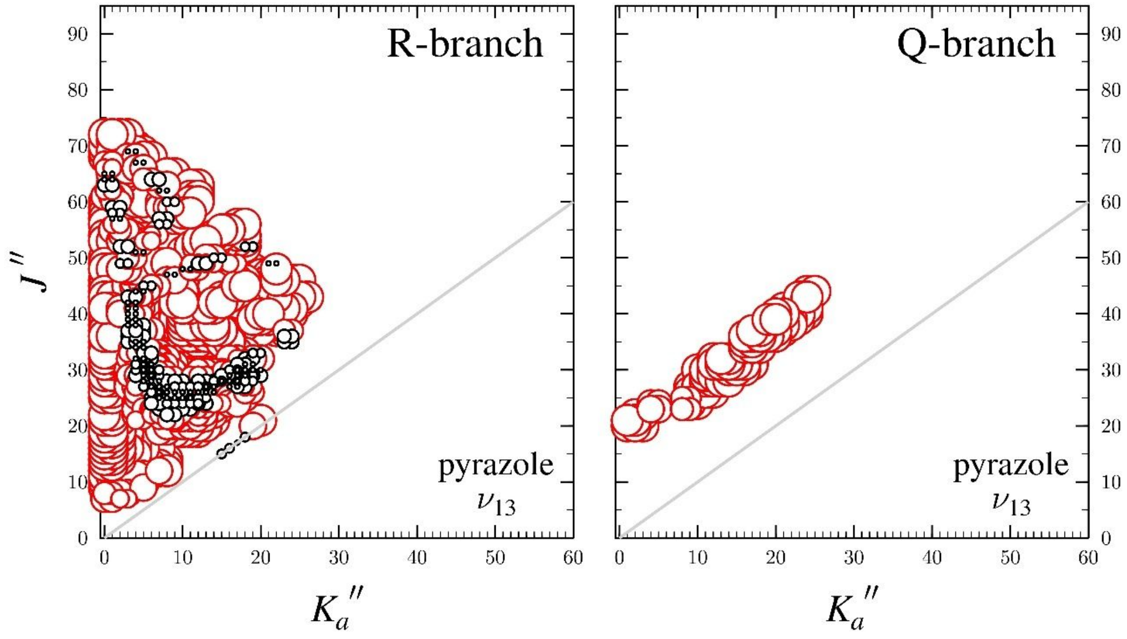

Figure S17. Data distribution plot for the fitted millimeter-wave spectroscopic data for the normal isotopologue of pyrazole, ν_{13}. The size of the outlined circle is proportional to the value of $|(f_{obs.} - f_{calc.})/\delta f|$, where δf is the frequency measurement uncertainty up to a quotient value of 6. Data points with of $|(f_{obs.} - f_{calc.})/\delta f| \geq 6$ use the same size of symbol for legibility. Data points with quotient values larger than 3 are shown in red.

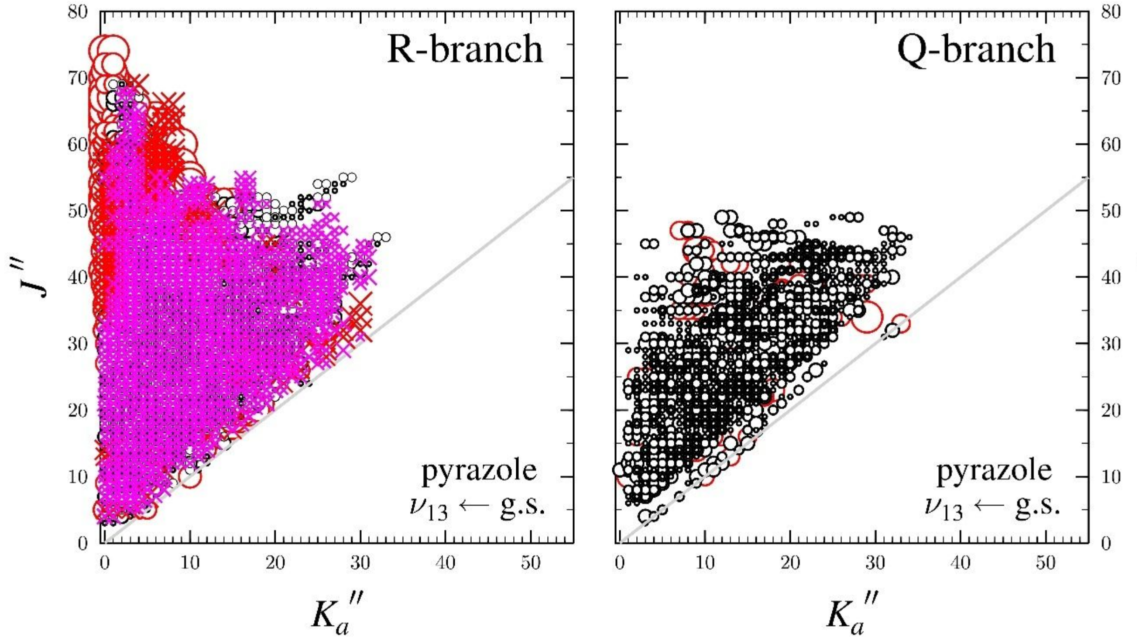

Figure S18. Data distribution plot for the least-squares fit of high-resolution infrared spectroscopic data for the normal isotopologue of pyrazole, $\nu_{13} \leftarrow$ g.s. Circles indicate R- and Q-branch transitions, while magenta crosses indicate P-branch transitions. The size of the outlined circle is proportional to the value of $|(f_{obs.} - f_{calc.})/\delta f|$, where δf is the frequency measurement uncertainty up to a quotient value of 6. Data points with of $|(f_{obs.} - f_{calc.})/\delta f| \geq 6$ use the same size of symbol for legibility. Data points with quotient values larger than 3 are shown in red.

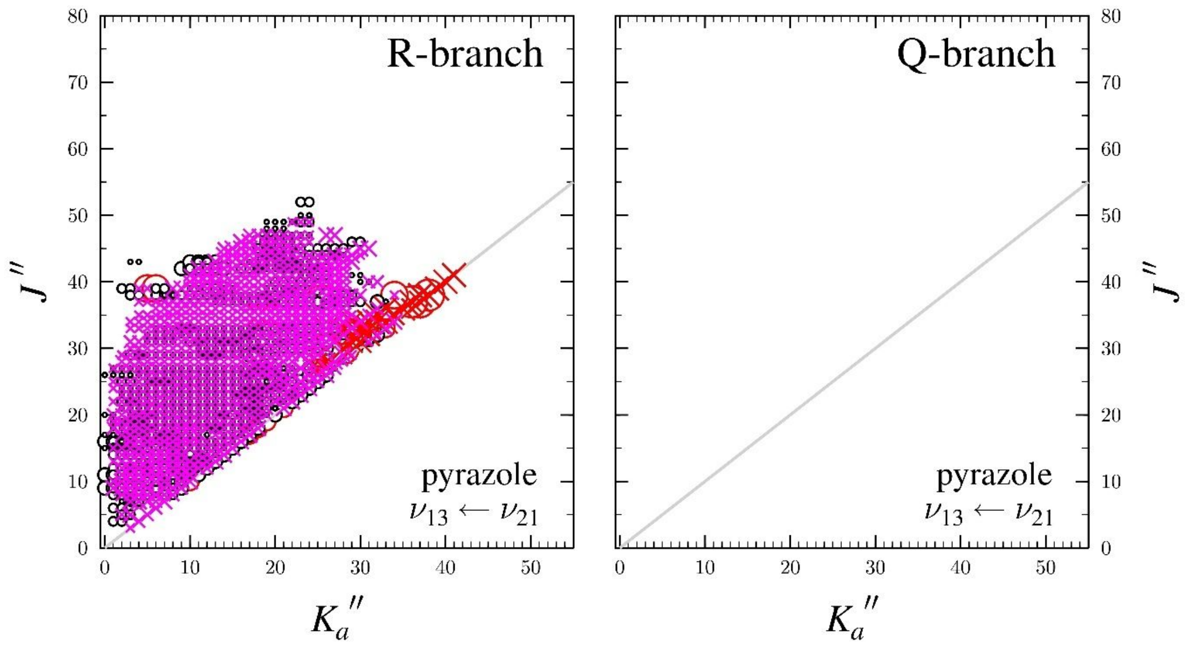

Figure S19. Data distribution plot for the fitted high-resolution infrared spectroscopic data for the normal isotopologue of pyrazole, $\nu_{13} \leftarrow \nu_{21}$. Circles indicate R- and Q-branch transitions, while magenta crosses indicate P-branch transitions. The size of the outlined circle is proportional to the value of $|(f_{obs.} - f_{calc.})/\delta f|$, where δf is the frequency measurement uncertainty up to a quotient value of 6. Data points with of $|(f_{obs.} - f_{calc.})/\delta f| \geq 6$ use the same size of symbol for legibility. Data points with quotient values larger than 3 are shown in red.

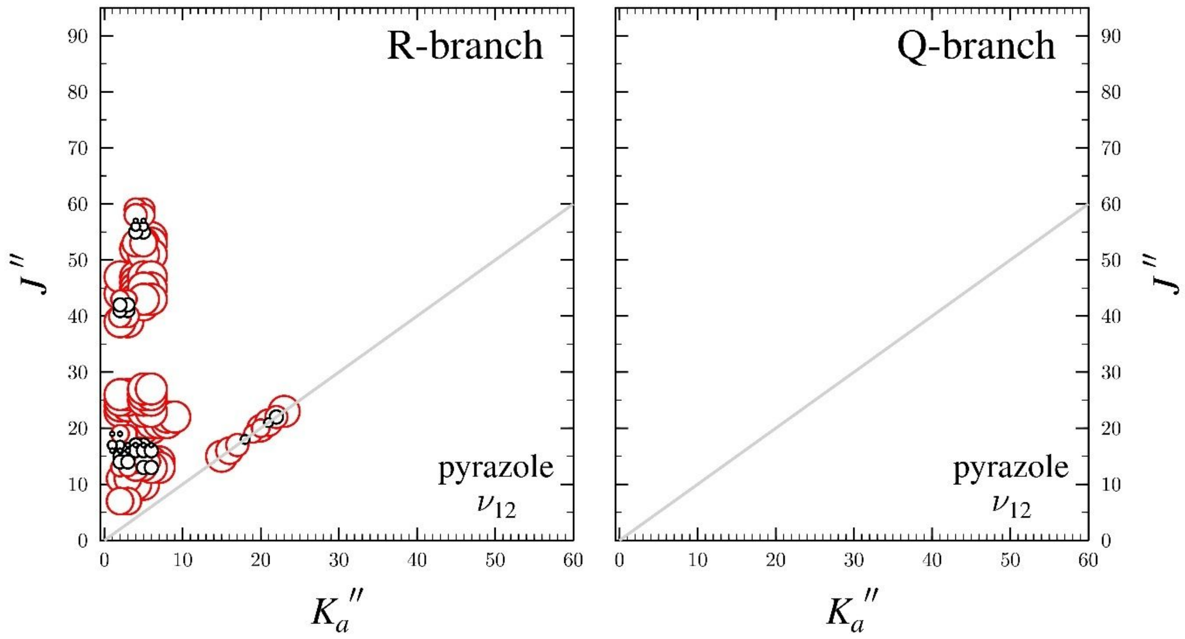

Figure S20. Data distribution plot for the fitted millimeter-wave spectroscopic data for the normal isotopologue of pyrazole, ν_{12}. The size of the outlined circle is proportional to the value of $|(f_{obs.} - f_{calc.})/\delta f|$, where δf is the frequency measurement uncertainty up to a quotient value of 6. Data points with of $|(f_{obs.} - f_{calc.})/\delta f| \geq 6$ use the same size of symbol for legibility. Data points with quotient values larger than 3 are shown in red.

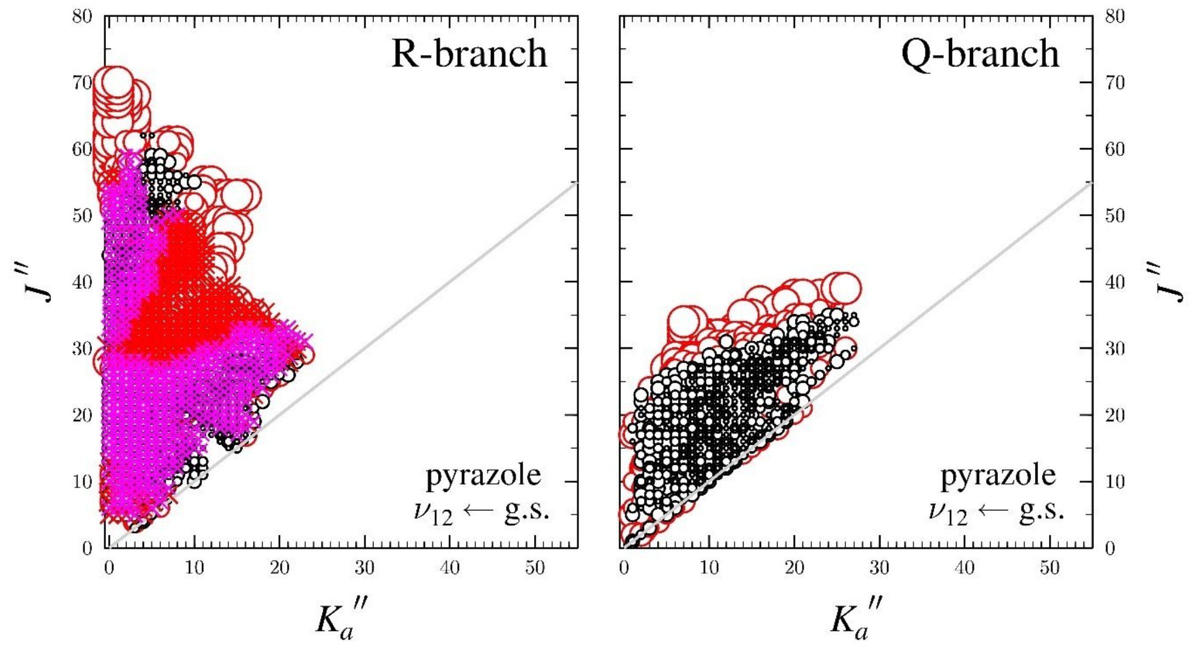

Figure S21. Data distribution plot for the least-squares fit of high-resolution infrared spectroscopic data for the normal isotopologue of pyrazole, $\nu_{12} \leftarrow$ g.s. Circles indicate R- and Q-branch transitions, while magenta crosses indicate P-branch transitions. The size of the outlined circle is proportional to the value of $|(f_{\text{obs.}} - f_{\text{calc.}})/\delta f|$, where δf is the frequency measurement uncertainty up to a quotient value of 6. Data points with of $|(f_{\text{obs.}} - f_{\text{calc.}})/\delta f| \geq 6$ use the same size of symbol for legibility. Data points with quotient values larger than 3 are shown in red.

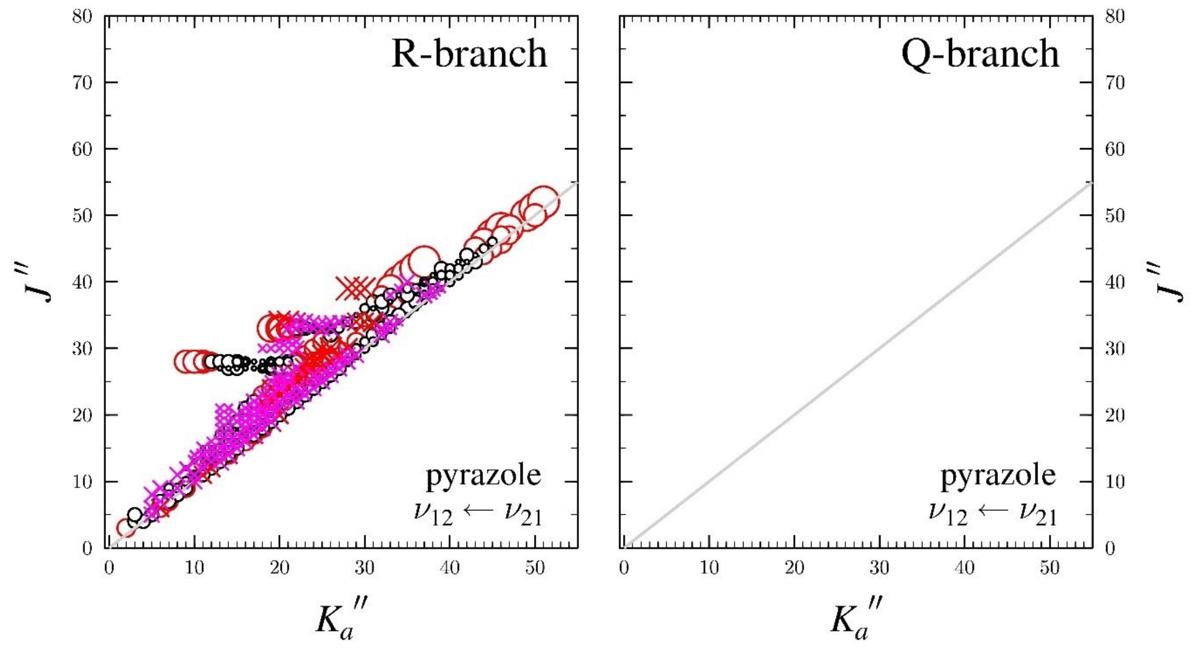

Figure S22. Data distribution plot for the fitted high-resolution infrared spectroscopic data for the normal isotopologue of pyrazole, $\nu_{12} \leftarrow \nu_{21}$. Circles indicate R- and Q-branch transitions, while magenta crosses indicate P-branch transitions. The size of the outlined circle is proportional to the value of $|(f_{obs.} - f_{calc.})/\delta f|$, where δf is the frequency measurement uncertainty up to a quotient value of 6. Data points with of $|(f_{obs.} - f_{calc.})/\delta f| \geq 6$ use the same size of symbol for legibility. Data points with quotient values larger than 3 are shown in red.

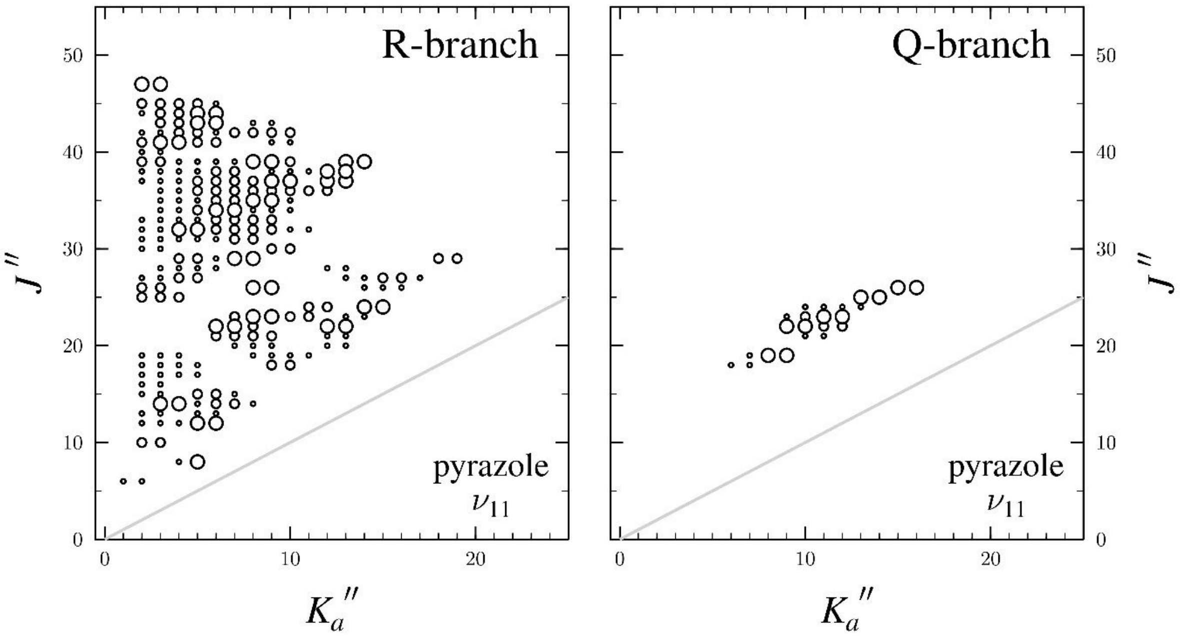

Figure S23. Data distribution plot for the fitted millimeter-wave spectroscopic data for the normal isotopologue of pyrazole, ν_{11}. The size of the outlined circle is proportional to the value of $|(f_{obs.} - f_{calc.})/\delta f|$, where δf is the frequency measurement uncertainty, and no measurements have a quotient value larger than 3.

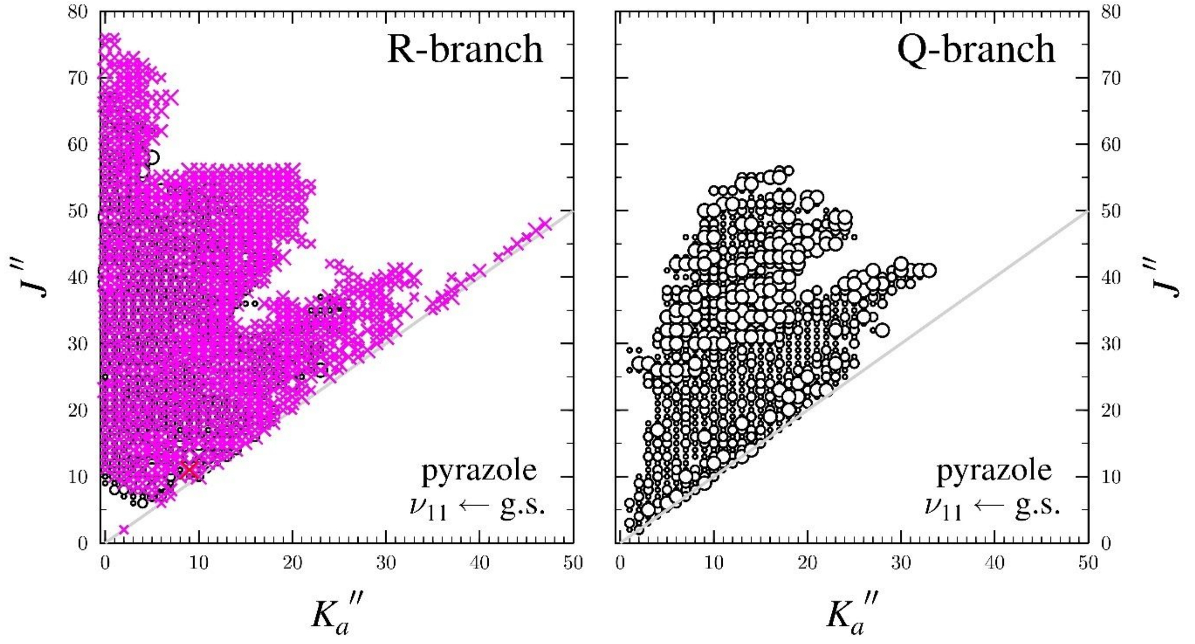

Figure S24. Data distribution plot for the least-squares fit of high-resolution infrared spectroscopic data for the normal isotopologue of pyrazole, $\nu_{11} \leftarrow$ g.s. Circles indicate R- and Q-branch transitions, while magenta crosses indicate P-branch transitions. The size of the symbol is proportional to the value of $|(f_{obs.} - f_{calc.})/\delta f|$, where δf is the frequency measurement uncertainty, and data points with quotient values larger than 3 are shown in red.

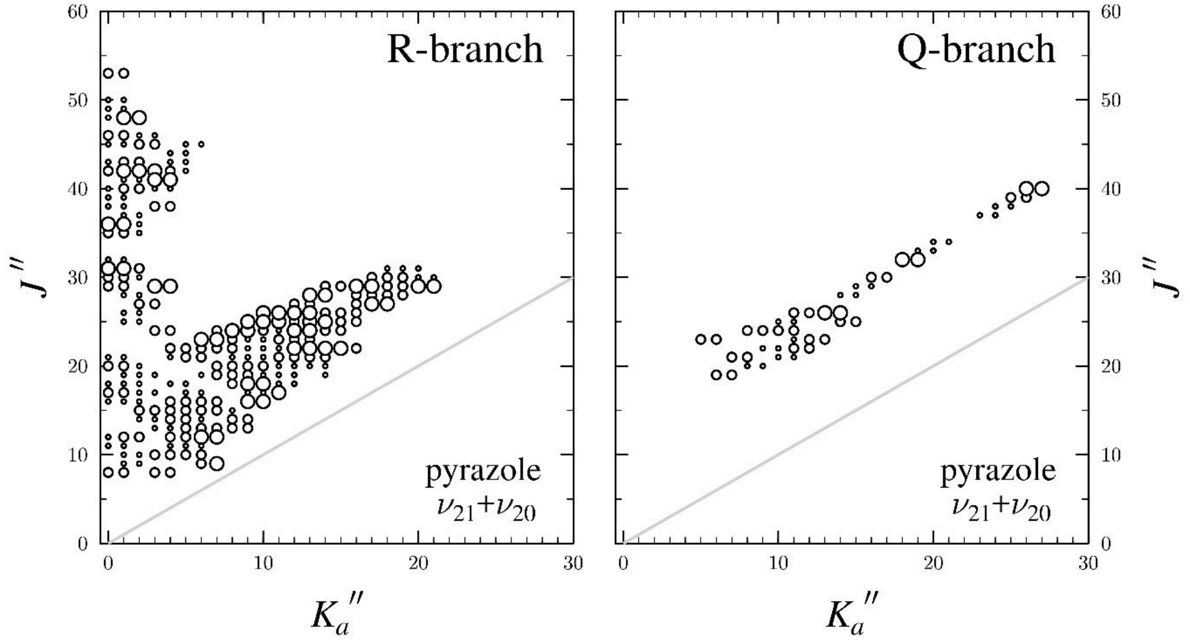

Figure S25. Data distribution plot for the fitted millimeter-wave spectroscopic data for the normal isotopologue of pyrazole, $\nu_{21}+\nu_{20}$. The size of the outlined circle is proportional to the value of $|(f_{obs.} - f_{calc.})/\delta f|$, where δf is the frequency measurement uncertainty, and no measurements have a quotient value larger than 3.

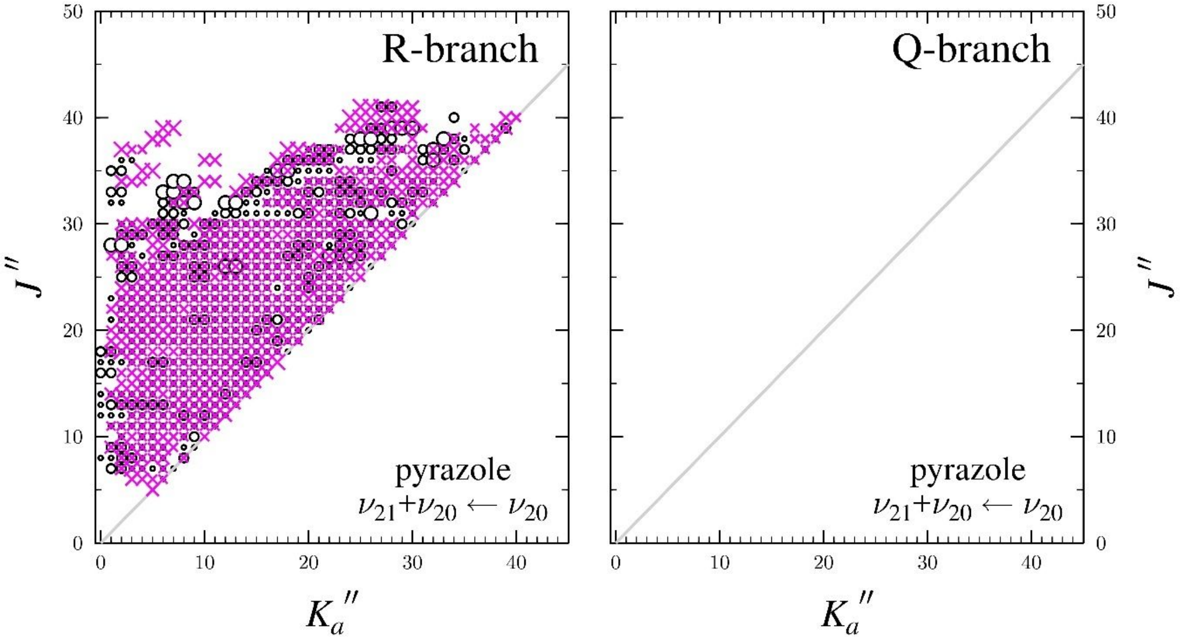

Figure S26. Data distribution plot for the fitted high-resolution infrared spectroscopic data for the normal isotopologue of pyrazole, $\nu_{21}+\nu_{20} \leftarrow \nu_{20}$. Circles indicate R- and Q-branch transitions, while magenta crosses indicate P-branch transitions. The size of the symbol is proportional to the value of $|(f_{obs.} - f_{calc.})/\delta f|$, where δf is the frequency measurement uncertainty, and no measurements have a quotient value larger than 3

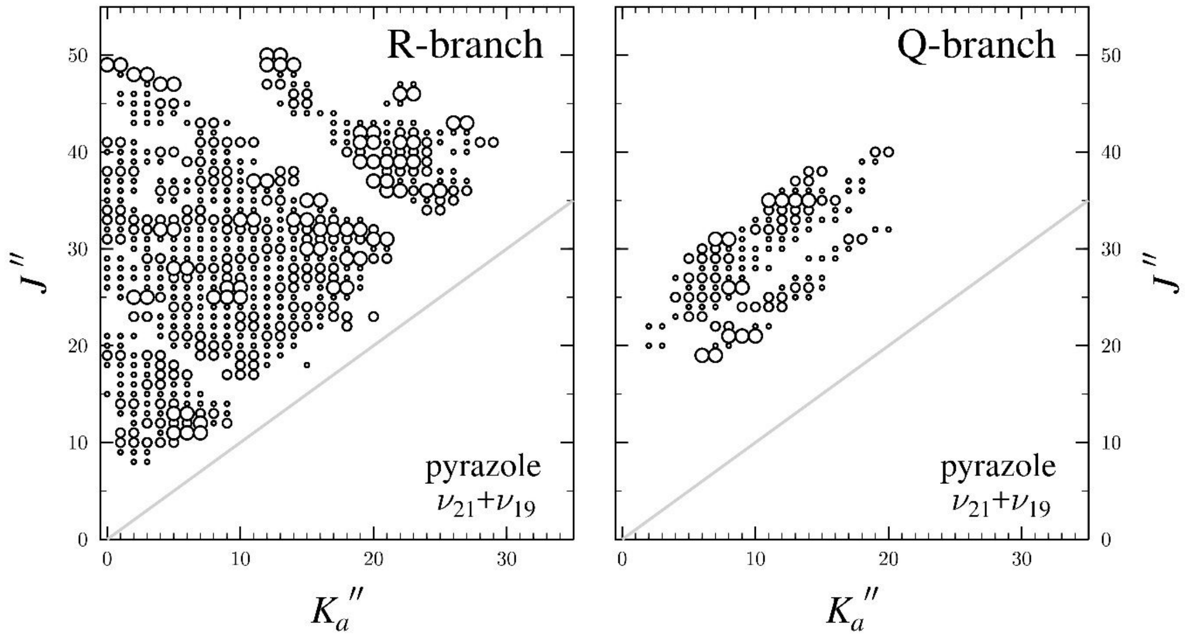

Figure S27. Data distribution plot for the fitted millimeter-wave spectroscopic data for the normal isotopologue of pyrazole, $\nu_{21}+\nu_{19}$. The size of the outlined circle is proportional to the value of $|(f_{obs.}-f_{calc.})/\delta f|$, where δf is the frequency measurement uncertainty, and no measurements have a quotient value larger than 3.

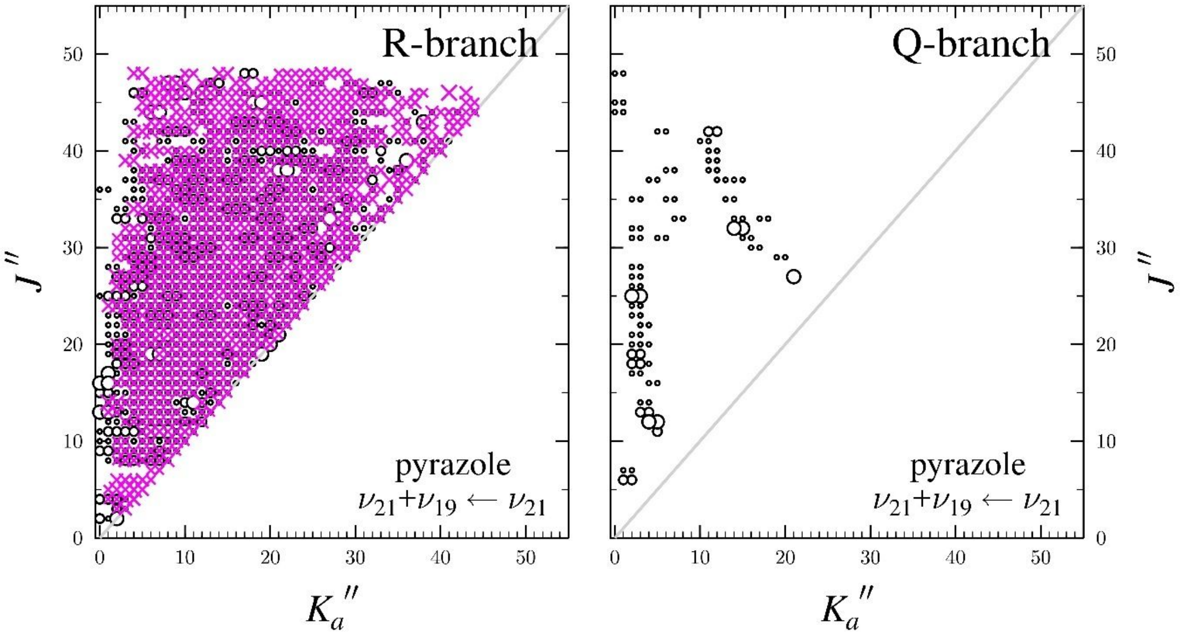

Figure S28. Data distribution plot for the fitted high-resolution infrared spectroscopic data for the normal isotopologue of pyrazole, $\nu_{21}+\nu_{19} \leftarrow \nu_{21}$. Circles indicate R- and Q-branch transitions, while magenta crosses indicate P-branch transitions. The size of the symbol is proportional to the value of $|(f_{obs.} - f_{calc.})/\delta f|$, where δf is the frequency measurement uncertainty, and no measurements have a quotient value larger than 3.

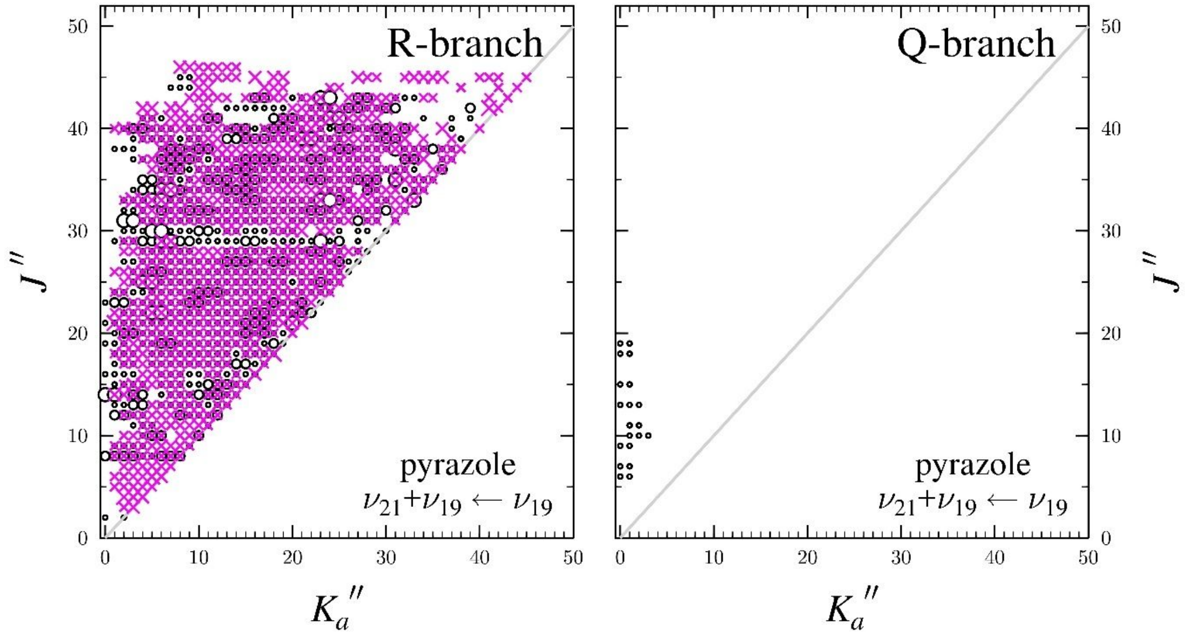

Figure S29. Data distribution plot for the fitted high-resolution infrared spectroscopic data for the normal isotopologue of pyrazole, $\nu_{21}+ \nu_{19} \leftarrow \nu_{19}$. Circles indicate R- and Q-branch transitions, while magenta crosses indicate P-branch transitions. The size of the symbol is proportional to the value of $|(f_{obs.} - f_{calc.})/\delta f|$, where δf is the frequency measurement uncertainty, and no measurements have a quotient value larger than 3.

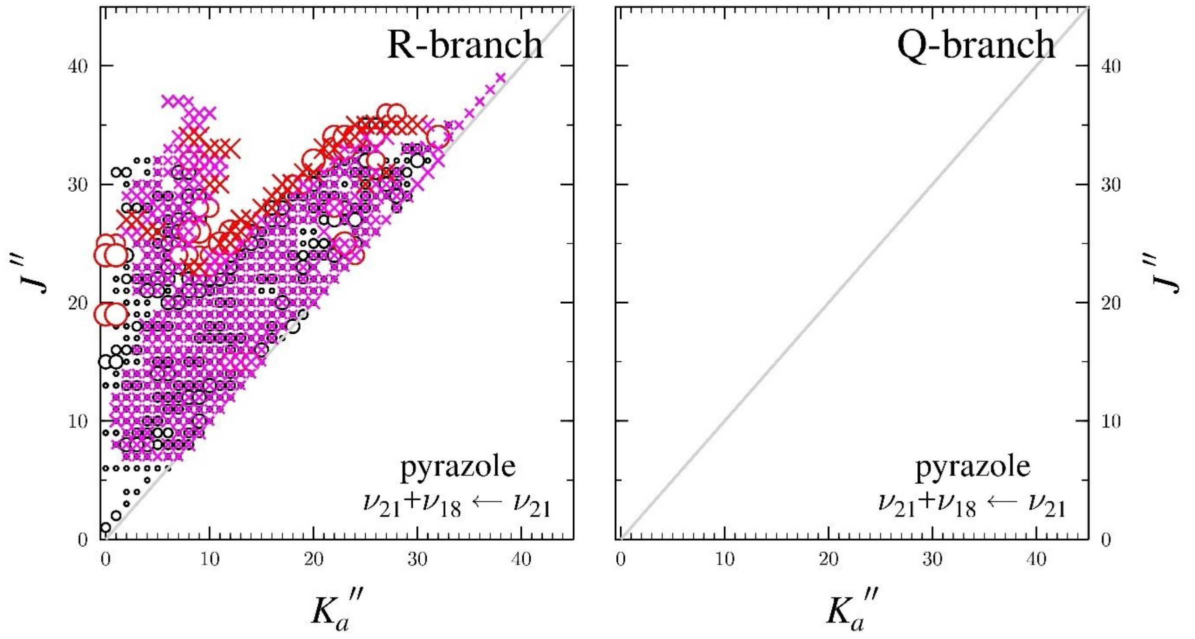

Figure S30. Data distribution plot for the fitted high-resolution infrared spectroscopic data for the normal isotopologue of pyrazole, $\nu_{21}+ \nu_{18} \leftarrow \nu_{21}$. Circles indicate R- and Q-branch transitions, while magenta crosses indicate P-branch transitions. The size of the symbol is proportional to the value of $|(f_{obs.} - f_{calc.})/\delta f|$, where δf is the frequency measurement uncertainty, and data points with quotient values larger than 3 are shown in red.

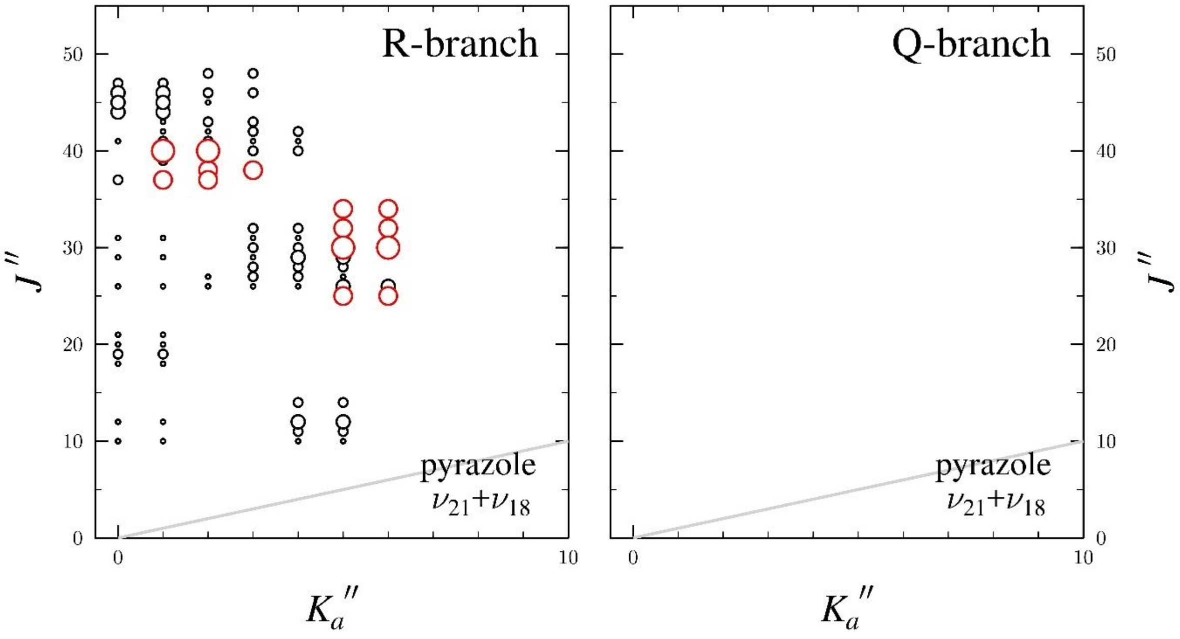

Figure S31. Data distribution plot for the fitted millimeter-wave spectroscopic data for the normal isotopologue of pyrazole, $\nu_{21}+\nu_{18}$. The size of the outlined circle is proportional to the value of $|(f_{obs.} - f_{calc.})/\delta f|$, where δf is the frequency measurement uncertainty, and data points with quotient values larger than 3 are shown in red.

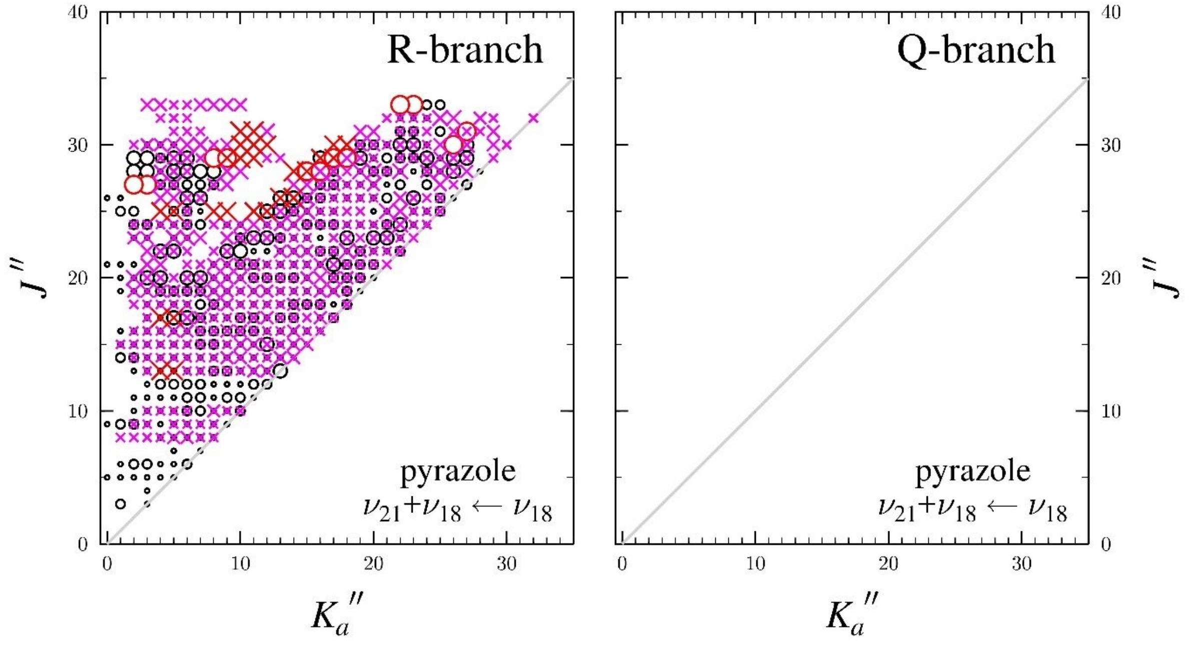

Figure S32. Data distribution plot for the fitted high-resolution infrared spectroscopic data for the normal isotopologue of pyrazole, $\nu_{21}+ \nu_{18} \leftarrow \nu_{18}$. Circles indicate R- and Q-branch transitions, while magenta crosses indicate P-branch transitions. The size of the symbol is proportional to the value of $|(f_{obs.} - f_{calc.})/\delta f|$, where δf is the frequency measurement uncertainty, and data points with quotient values larger than 3 are shown in red.

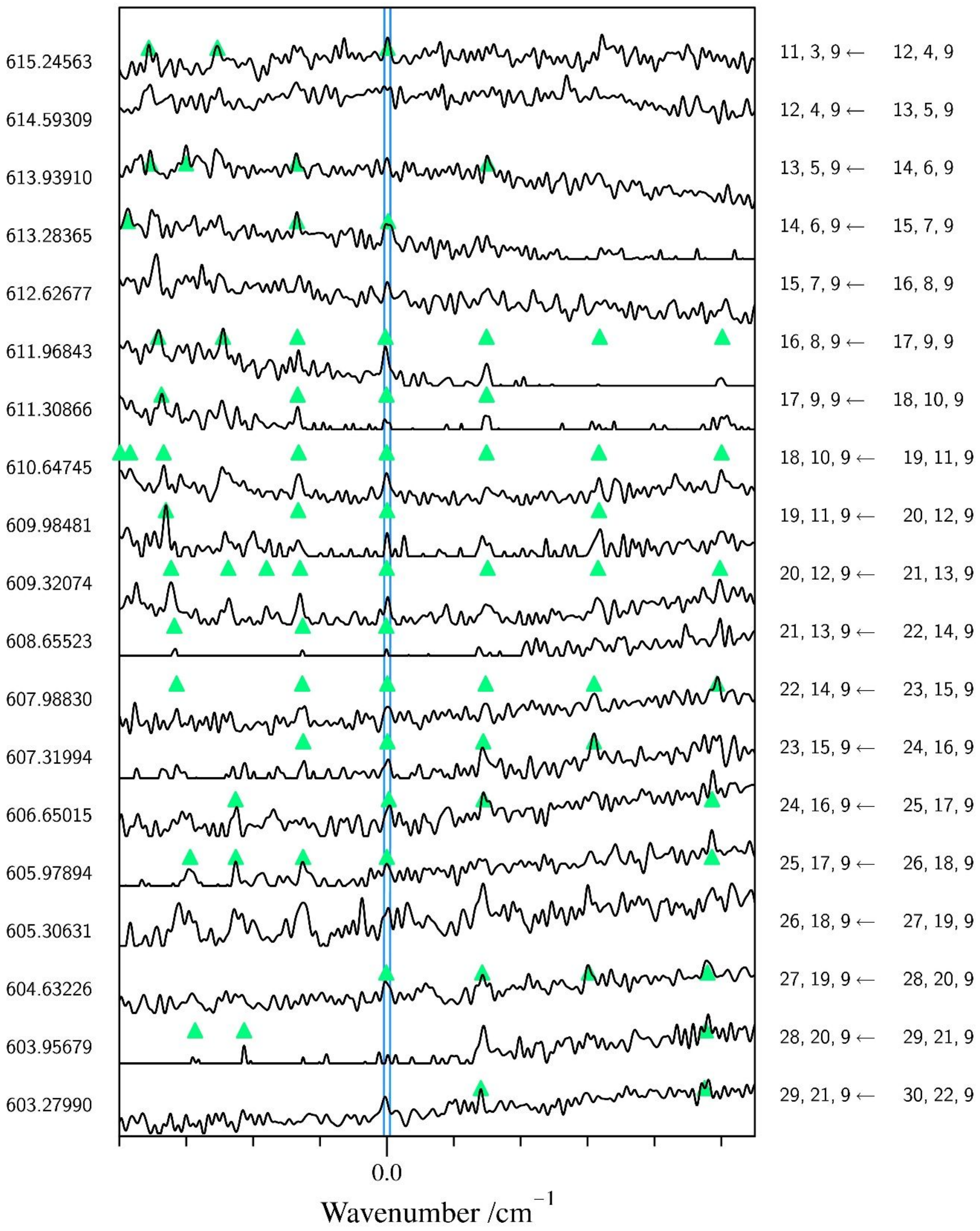

Figure S33. Loomis-Wood plot focused on a *c*-type P-branch series ($K_c = 9$) of $\nu_{20} \leftarrow$ g.s. of pyrazole with other series visible. Measured transitions are marked by green triangles.

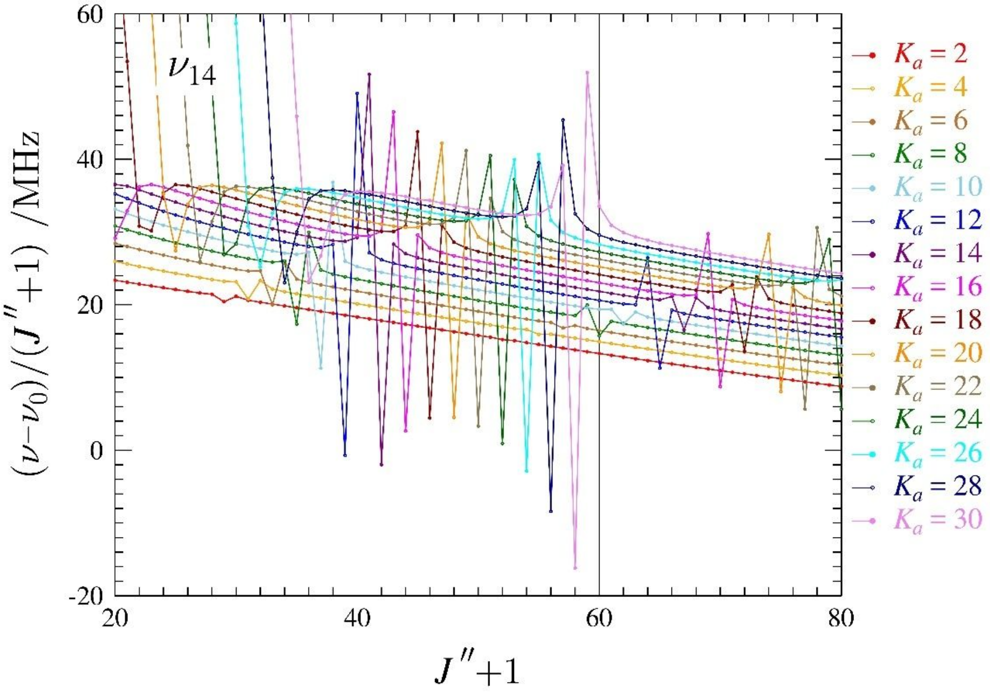

Figure S34. Superimposed resonance plots of ν_{14} for ${}^{a}R_{0,1}$ even-K_a^+ series from 2 to 30 for pyrazole. Measured transitions are omitted for clarity, but they are indistinguishable from the plotted values on this scale. The plotted values are frequency differences between excited-state transitions and their ground-state counterparts ($\nu - \nu_0$), scaled by (J'' + 1).

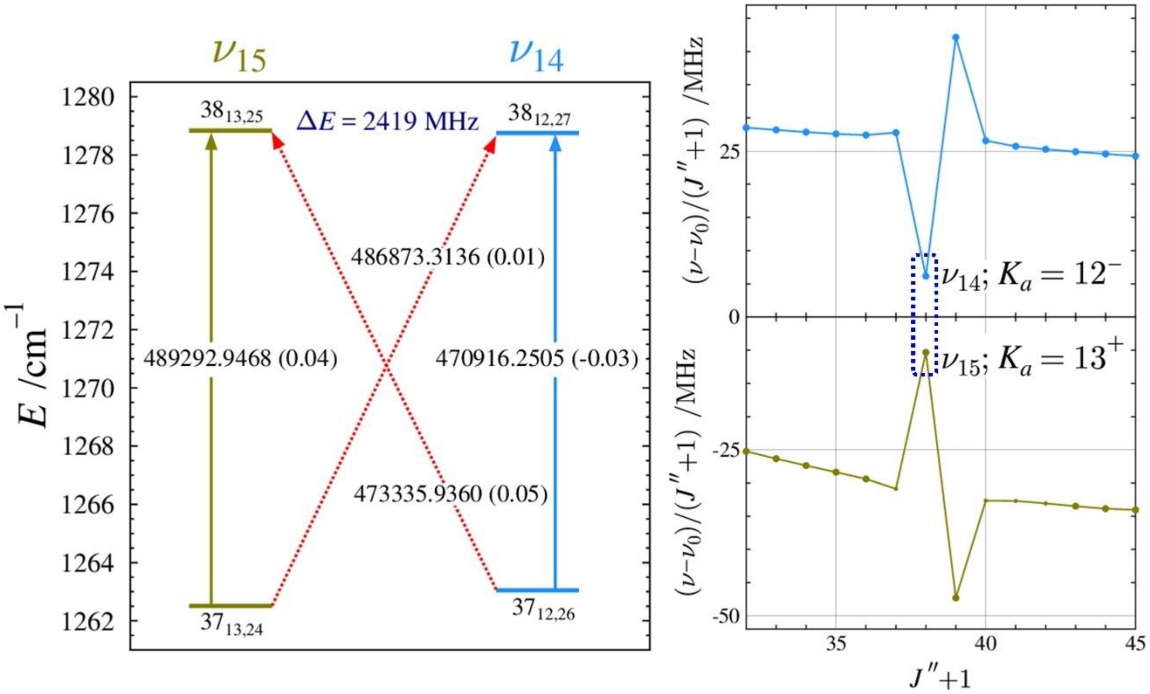

Figure S35. Energy diagram (left) depicting a representative matched pair of nominal interstate transitions between the ν_{15} (olive) and ν_{14} (light blue) vibrationally excited states of pyrazole. Standard $^{a}R_{0,1}$ transitions within vibrational states are denoted by vertical arrows. The diagonal, dashed arrows indicate nominal interstate transitions that are formally forbidden but allowed as a result of rotational energy-level mixing. Values printed on each of the arrows are the corresponding transition frequency (in MHz) with the corresponding *obs*. – *calc*. value in parentheses. The marked energy separation is between the two strongly interacting rotational energy levels. Resonance plots (right) of the K_a series of ν_{14} and ν_{15} show the corresponding resonant intrastate transitions.

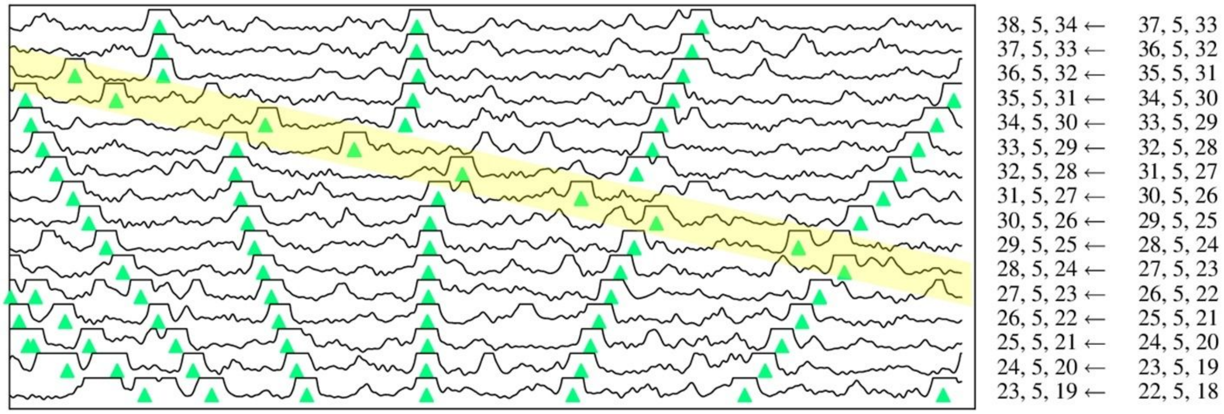

Figure S36. Loomis-Wood plot of an *a*-type R-branch series ($K_a = 5$) of $\nu_{12} \leftarrow$ g.s. of pyrazole. Measured transitions are marked by green triangles. Resonances (a divergence in each depicted series) are visible, highlighted in yellow.

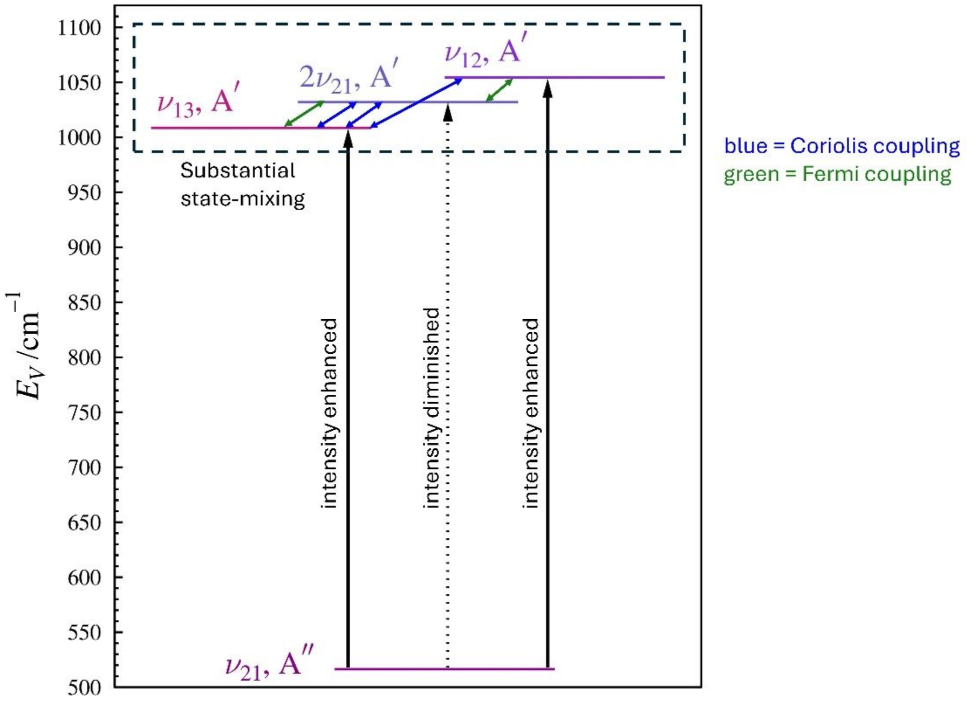

Figure S37. Energy diagram representing the coupling interactions of $2\nu_{21}$, ν_{12}, and ν_{13} that results in intensity borrowing to the $\nu_{12}-\nu_{21}$ and $\nu_{13}-\nu_{21}$ difference bands.

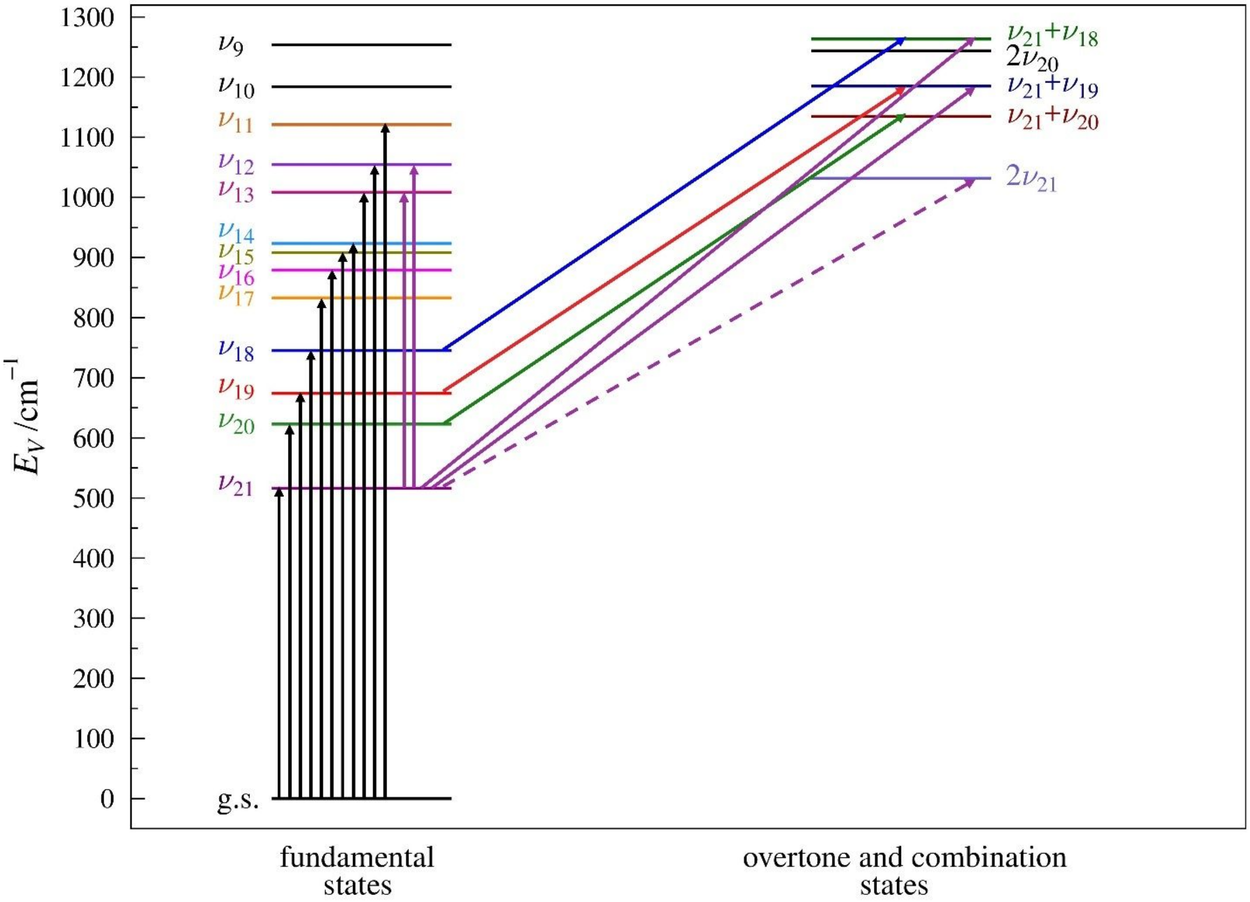

Figure S38. Energy level diagram showing the observed IR bands of pyrazole in this work (solid arrows). Arrow colors match the color of the lower energy vibrational state. The dashed arrow indicates the missing band of $2\nu_{21}-\nu_{21}$.

Table S1. Spectroscopic constants for the ground vibrational state of pyrazole (S reduction, III^r representation).

	B3LYP [a]	This work [b]
$A_0^{(S)}$ (MHz)	9581	9618.775 628 (40)
$B_0^{(S)}$ (MHz)	9394	9412.541 470 (39)
$C_0^{(S)}$ (MHz)	4741	4755.849 468 (43)
D_J (kHz)	3.23	3.276 232 (20)
D_{JK} (kHz)	−5.09	−5.164 096 (33)
D_K (kHz)	2.20	2.237 725 (20)
d_1 (kHz)	0.066 9	0.056 349 8 (53)
d_2 (kHz)	0.037 1	0.036 525 7 (19)
H_J (Hz)	0.001 37	0.001 377 7 (30)
H_{JK} (Hz)	−0.005 63	−0.005 731 3 (67)
H_{KJ} (Hz)	0.007 15	0.007 322 8 (67)
H_K (Hz)	−0.002 89	−0.002 978 6 (30)
h_1 (Hz)	0.000 011 8	[0.000 005 5] [c]
h_2 (Hz)	−0.000 084 1	[−0.000 049 7] [c]
h_3 (Hz)	0.000 002 57	0.000 006 38 (89)
N_{lines} rot [d]		4456
σ_{fit} (MHz)		0.037
κ [e]		0.915 181
Δ_i (uÅ^2) [f]		0.031 743 (1)

[a] Evaluated with the 6-311+G(2d,p) basis set. Rotational constants (B_0) are determined from their equilibrium (B_e) values corrected for vibration-rotation interaction. [b] Includes transitions from previous works. [1-3] [c] Value held constant at the corresponding computed value.
[d] Number of independent transitions. [e] $\kappa = (2B - A - C)/(A - C)$ calculated from B_0 values using PLANM. [f] Inertial defect ($\Delta_i = I_c - I_a - I_b$) calculated from B_0 values using PLANM.

Table S2. Spectroscopic constants for the ground and ν_{21} vibrational states of pyrazole with Φ_{JK} of ν_{21} held constant and allowed to fit (A reduction, I^r representation)

	**ground state**	**ν_{21} (A″)**	**ν_{21} (A″)**
$A_v^{(A)}$ (MHz)	9618.773 480 (53)	9602.905 114 (67)	9602.905 131 (74)
$B_v^{(A)}$ (MHz)	9412.543 971 (48)	9387.850 520 (56)	9387.850 499 (68)
$C_v^{(A)}$ (MHz)	4755.849 164 (43)	4754.105 442 (43)	4754.105 443 (43)
Δ_J (kHz)	1.832 963 (25)	1.839 073 (26)	1.839 058 (37)
Δ_{JK} (kHz)	−0.615 627 (93)	−0.616 521 (69)	−0.616 47 (12)
Δ_K (kHz)	1.873 249 (86)	1.858 520 (71)	1.858 497 (83)
δ_J (kHz)	0.741 551 (10)	0.743 077 (11)	0.743 069 (18)
δ_K (kHz)	1.276 153 (27)	1.272 347 (24)	1.272 346 (24)
Φ_J (Hz)	0.000 658 4 (53)	0.000 734 1 (43)	0.000 729 5 (96)
Φ_{JK} (Hz)	0.000 266 (40)	[0.] [a]	0.000 018 (34)
Φ_{KJ} (Hz)	−0.005 582 (52)	−0.005 577 (55)	−0.005 578 (55)
Φ_K (Hz)	0.006 135 (44)	0.006 234 (58)	0.006 224 (62)
ϕ_J (Hz)	0.000 333 6 (25)	0.000 3703 (21)	0.000 3680 (48)
ϕ_{JK} (Hz)	0.000 564 (14)	[0.000 564] [a]	[0.000 564] [a]
ϕ_K (Hz)	0.002 394 (15)	0.002 432 (22)	0.002 436 (23)
ν (cm^{-1})		516.345 927 3 (13)	516.345 927 3 (13)
N_{lines} (rot) [b]	4456	3247	3247
N_{lines} (IR) [b]		4894	4894
σ_{fit} rot (MHz)	0.037	0.033	0.033
σ_{fit} IR (MHz)		2.1	2.1
κ [c]	0.915 183	0.911 296	0.911 296
Δ_i (uÅ^2) [d]	0.031 753 (1)	−0.157 322 (1)	−0.157 322 (1)

[a] Value held constant at zero (see discussion of constants and fitting) or the corresponding ground-state value. [b] Number of independent transitions.
[c] $\kappa = (2B - A - C)/(A - C)$ [d] Inertial defect ($\Delta_i = I_c - I_a - I_b$)

Table S3. Extracted and fit spectroscopic constants (A Reduction, I^r Representation) for assigned combination states of pyrazole. [a]

	$\nu_{21}+\nu_{20}$ (A′)		$\nu_{21}+\nu_{19}$ (A′)		$\nu_{21}+\nu_{18}$ (A′)	
	Extrapolate	Effective	Extrapolated	Effective	Extrapolated	Effective
$A_v^{(A)}$ (MHz)	9583.6	9586.272 1 (22)	9578.8	9580.581 7 (11)	9583.7	9585.898 3 (35)
$B_v^{(A)}$ (MHz)	9362.0	9366.895 6 (21)	9357.1	9359.017 6 (11)	9370.7	9373.423 0 (40)
$C_v^{(A)}$ (MHz)	4755.9	4756.117 39 (23)	4755.1	4755.019 26 (12)	4756.3	4755.345 25 (49)
Δ_J (kHz)	1.822	1.778 3 (10)	1.793	1.782 72 (42)	1.819	1.610 5 (25)
Δ_{JK} (kHz)	−0.564	−0.611 6 (50)	−0.417	−0.414 8 (20)	−0.605	−0.165 (11)
Δ_K (kHz)	1.809	1.773 5 (40)	1.648	1.634 3 (19)	1.792	1.382 (10)
δ_J (kHz)	0.734	0.710 56 (51)	0.719	0.714 41 (21)	0.732	0.636 5 (12)
δ_K (kHz)	1.222	1.190 3 (12)	1.223	1.212 85 (54)	1.206	0.968 7 (29)
Φ_J (Hz)	0.001 16	[0.001 16]	0.000 783	[0.000 783]	0.000 825	[0.000 825]
Φ_{JK} (Hz)	−0.001 71	[−0.001 71]	−0.000 653	[−0.000 653]	−0.000 770	[−0.000 770]
Φ_{KJ} (Hz)	−0.004 66	[−0.004 66]	−0.003 76	[−0.003 76]	−0.005 68	[−0.005 68]
Φ_K (Hz)	0.005 64	[0.005 64]	0.004 25	[0.004 25]	0.006 36	[0.006 36]
ϕ_J (Hz)	0.000 578	[0.000 578]	0.000 387	[0.000 387]	0.000 4122	[0.000 412]
ϕ_{JK} (Hz)	0.000 564	[0.000 564]	0.000 564	[0.000 564]	0.000 564	[0.000 564]
ϕ_K (Hz)	0.002 43	[0.002 43]	0.001 40	[0.001 40]	0.001 84	[0.001 84]
ν (cm^{-1})		1134.850 562 2 (60)		1185.053 561 0 (37)		1263.475 638 (12)
N_{lines} (rot) [b]		196		494		53
N_{lines} (IR) [b]		1081		1521 / 1328 [c]		800 / 649 [c]
σ_{fit} rot (MHz)		0.072		0.056		0.091
σ_{fit} IR (MHz)		3.5		3.6 / 3.2 [c]		6.1 / 6.1 [c]
k [d]	0.908 196	0.909 164	0.908 079	0.908 171	0.911 754	0.912 029
Δ_i (uÅ^2) [e]	−0.452 093	−0.414 037 (18)	−0.488 908	−0.466 226 (9)	−0.410 361	−0.361 267 (32)

[a] Extrapolated values are calculated using unrounded values of constants in PAR files.
[b] Number of independent transitions.
[c] The first value relates to transitions between the combination state and ν_{21}; the second value relates to transitions between the combination state and other fundamental state.
[d] $k = (2B - A - C)/(A - C)$
[e] Inertial defect ($\Delta_i = I_c - I_a - I_b$)

Table S4. Computed anharmonic vibrational frequencies and infrared intensities.

	CCSD(T) [a]		B3LYP [b]	
	Frequency (cm^{-1})	Intensity (km/mol)	Frequency (cm^{-1})	Intensity (km/mol)
ν_{21}	504.4	53.4	512.5	61.5
ν_{20}	622.4	0.3	625.8	0.3
ν_{19}	674.6	18.2	673.7	7.4
ν_{18}	744.5	85.8	739.0	104.0
ν_{17}	828.9	6.3	823.0	9.0
ν_{16}	875.1	6.0	883.3	2.7
ν_{15}	904.5	9.2	914.1	9.9
ν_{14}	919.7	4.3	929.4	3.8
ν_{13}	1044.7 [c]	3.3	1032.5	11.2
$2\nu_{21}$	996.8	90.9	1038.0	71.4
ν_{12}	1037.6 [c]	5.8	1035.6	7.6
ν_{11}	1159.0 [c]	0.4	1111.4	19.6
$\nu_{21}+\nu_{20}$	1084.9	2476.2 [d]	1135.1	0.5
ν_{10}	1151.9 [c]	**** [d]	1145.1	0.3
$\nu_{21}+\nu_{19}$	1174.8	0.9	1184.0	0.6
$\nu_{21}+\nu_{18}$	1249.2	0.3	1253.5	0.0

[a] Evaluated with the cc-pCVTZ basis set. [b] Evaluated with the 6-311G+(2d,p) basis set. [c] State order changes from harmonic to anharmonic frequency value. State labels based upon harmonic energy ordering. [d] Unreasonable anharmonic intensities due to untreated resonances.

References

(1) Blackman, G. L.; Brown, R. D.; Burden, F. R. The quadrupole hyperfine structure of the microwave spectrum of pyrazole. *J. Mol. Spectrosc.* **1970**, *36*, 528-540. DOI: https://doi.org/10.1016/0022-2852(70)90226-2.

(2) Wlodarczak, G.; Demaison, J.; van Eijck, B. P.; Zhao, M.; Boggs, J. E. Ab initio and experimental quartic centrifugal distortion constants of acetone, pyrazole, and γ-pyrone. *J. Chem. Phys.* **1991**, *94*, 6698-6707. DOI: 10.1063/1.460246.

(3) Kirchhoff, W. H. The Microwave Spectrum and Dipole Moment of Pyrazole. *J. Am. Chem. Soc.* **1967**, *89*, 1312-1316. DOI: 10.1021/ja00982a006.
